# Assessing the clinical, humanistic, and economic impact of early cancer diagnosis: a systematic literature review

**DOI:** 10.3389/fonc.2025.1546447

**Published:** 2025-03-19

**Authors:** Raquel Aguiar-Ibáñez, Yves Paul Vincent Mbous, Sugandh Sharma, Evanka Chawla

**Affiliations:** ^1^ Merck Canada Inc., Kirkland, QC, Canada; ^2^ Merck & Co., Inc., Rahway, NJ, United States; ^3^ Parexel International, Mohali/Chandigarh, India

**Keywords:** early diagnosis, benefits, survival, healthcare cost, healthcare resource utilization, health related quality of life

## Abstract

**Introduction:**

There is a clear consensus among healthcare providers on the advantages of early cancer detection and treatment. However, no in-depth review has yet fully presented the clinical, humanistic, and economic benefits of early cancer diagnosis compared to late detection across a broad range of tumor types.

**Methods:**

A systematic literature review was conducted to determine the clinical, humanistic, and economic benefits of early cancer diagnosis, as opposed to late diagnosis, as reported in non-interventional studies conducted worldwide. Searches were conducted using electronic databases (MEDLINE and Embase), conference repositories and grey literature. Observational studies in adults diagnosed with bladder cancer, gastric cancer, head and neck cancer (HNC), melanoma, non-small cell lung cancer (NSCLC), renal-cell carcinoma (RCC), and triple negative breast cancer (TNBC) were eligible for inclusion if they reported survival, health-related quality of life (HRQoL), healthcare resource utilization and/or costs, according to stage at diagnosis. Identified records were screened and extracted by two independent reviewers, and discrepancies were resolved by a third reviewer. The quality of studies was assessed using the Newcastle-Ottawa scale and the Larg and Moss adapted checklist.

**Results:**

Of the 3,159 records identified, 103 studies were included in this review. The general trend showed worse clinical, humanistic, and economic outcomes when patients were diagnosed at a later stage compared to an earlier stage. Patients diagnosed at an earlier stage, had on average, substantially higher survival rates and lower mortality rates across all cancer types and incurred lower resource utilization and costs (with available evidence for patients with NSCLC, TNBC, and HNC), compared to those diagnosed at a more advanced/later stage. Limited evidence on the humanistic burden suggested that with a more advanced stage at diagnosis, patients with bladder cancer experienced reduced HRQoL.

**Conclusion:**

Early cancer diagnosis (i.e., cancer diagnosed at earlier stages or with lower grades) was associated with longer survival, improved quality of life and lower healthcare costs and resource utilization compared to diagnosis of cancer at later stages or higher grades, as reported by overall survival (OS) and HRQoL outcomes. These findings emphasize the importance of screening and early detection of cancer to improve outcomes among patients diagnosed with cancer.

## 1 Introduction

Cancer represents a major public health concern, with over 20 million cases worldwide in 2022, increasing to an expected 35 million cases by 2050 (1). In the United States (US) alone, there will be over 2 million new cancer cases diagnosed by the end of 2024 (2). Thus, it is expected that the cancer burden will continue to exert a substantial clinical, humanistic, and financial burden on patients, their caregivers, their communities and health systems (3). One of the proposed strategies to lessen cancer burden is to diagnose the disease as early as possible, as it is generally easier to treat cancer when it is localized as opposed to when it has spread, and results in better patient outcomes (4–6). The focus of early cancer diagnosis is to identify disease among symptomatic individuals at its earliest stages (preferably onset), to swiftly and effectively streamline treatment. The World Health Organization (WHO) states that some of the benefits of early diagnosis may include: 1) a reduction in stage of disease at diagnosis and 2) with no treatment delay, a reduction in mortality that is evident 3 to 5 years post-diagnosis (7).

Although there is limited evidence available directly comparing outcomes between patients diagnosed at earlier versus at more advanced stages, or presenting the outcomes in a comprehensive manner for different tumor types, available publications suggest that early cancer diagnosis is not only associated with improved survival, but importantly, improved experiences of care, lower treatment morbidity, and improved quality of life compared to late cancer diagnosis (2, 8). In terms of survival, 90% of patients diagnosed with early-stage breast cancer, 90% of those diagnosed with early-stage ovarian cancer, and 70% of patients diagnosed with early-stage lung cancer will survive 5 years post-diagnosis, compared to 15%, 5% and 9% of those diagnosed with late stage breast, ovarian and lung cancer, respectively (6). Beyond the clinical benefits, early diagnosis is also associated with reduced cost and may help to alleviate the substantial economic burden associated with cancer at a healthcare system level (9). In the US, it was estimated that if all cases of melanoma, breast, lung and colorectal cancers were diagnosed at stages I or II, the national cost-savings would range from $1.56 to $3.47 billion dollars (10). Therefore, accelerating the diagnosis of symptomatic cancer seems to offer better outcomes for patients, healthcare systems and society overall.

The epidemiology of cancer by stage at the time of diagnosis varies across cancer types, and despite advances in diagnostic tools, the majority of patients diagnosed with certain cancer types (for example, lung, pancreatic and ovarian cancers) are still detected at an advanced stage, leading to poor prognoses and outcomes (11, 12). Moreover, once diagnosed, even a 4-week delay in initiating treatment substantially increases the mortality risk of patients, independent of the treatment they eventually receive (surgery, radiotherapy, or systemic therapy), which highlights the importance of minimizing delays related to cancer diagnosis and treatment (13).

Implementation of evidence-based prevention strategies including the identification and minimization of risk factors may assist in the reduction of cancer burden. With the availability of different modalities of cancer treatment, early diagnosis is essential to facilitate timely access to appropriate treatment regimens for patients, particularly with the increasing availability of neoadjuvant and adjuvant therapy options (14, 15). Current diagnosis policies are shifting towards supporting earlier identification and treatment of patients with cancer, including initiatives such as the Healthy People 2030 and the Cancer Moonshot, as well as fundraising efforts to support diagnostic blood and biopsy testing (16–18). However, one of the biggest obstacles to further investment remains the largely unknown effects of early diagnosis across different tumor types (19). Evidence on mortality, health-related quality of life (HRQoL) and financial burden are generally not sufficiently reported across different stages of disease. Moreover, not all patients with cancer benefit equally from early diagnosis. A patient is most likely to benefit from early cancer detection if: 1) their tumor type is common, 2) their cancer-related signs and symptoms are easily recognizable, and 3) effective current therapy is available and administered timeously after diagnosis.

To strengthen early diagnosis adoption and policies, an accurate and up-to-date understanding of its impact is necessary. Whilst there is a growing body of evidence around the impact of diagnosing cancer in an early stage of the disease versus later stages, to the best of our knowledge, there has been no systematic literature review (SLR) published to date presenting a broad, pan-tumor overview of the benefits of early diagnosis. Existing literature reviews on this topic are sparse, and of limited scope, typically focusing on a single cancer classification or specific malignant conditions (20–27). Evidence characterizing the benefits of early cancer diagnosis remains inconsistent and fragmented, as there is a notable absence of comprehensive studies focusing specifically on the comparative benefits of early versus late diagnosis across multiple tumor types.

This study aims to summarize the literature on the benefits associated with cancer diagnoses at earlier stage, compared to later stages of disease, from clinical, humanistic, and economic/financial perspectives across bladder cancer, gastric cancer, head and neck cancer (HNC), melanoma, non-small cell lung cancer (NSCLC), renal cell carcinoma (RCC), and triple negative breast cancer (TNBC).

## 2 Methods

### 2.1 Data sources and search strategy

A systematic review was conducted following the guidelines in the Cochrane Handbook for Systematic Reviews and the Centre for Reviews and Dissemination (28, 29) and was reported according to the Preferred Reporting Items for Systematic Reviews and Meta-Analyses (PRISMA) (30). Comprehensive literature searches (searched from database inception until 30^th^ May 2022) were conducted using MEDLINE^®^, Embase^®^, and PubMed (only to identify in-process, and “Ahead of Print” citations) search engines. These searches were supplemented by grey literature screening from conference proceedings (2018 and 2022, inclusive) and from other sources including citation indexes, clinical and literature databases, and reference harvesting (2018 and 2022, inclusive). A systematic search was designed for each of the electronic databases searched; the search terms used included keywords and medical subject headings (MeSH terms) (**Supplementary Appendix S1**).

### 2.2 Eligibility criteria for study selection

Observational studies were included if they evaluated adults (≥18 years) diagnosed with one of the following cancers: bladder cancer, gastric cancer, HNC, NSCLC, melanoma, RCC, or TNBC and reported any of the outcomes of interest according to stage of disease at the time of diagnosis. These seven cancer types were selected since these are tumor types for which novel therapies (such as immunotherapies) have been approved or are being investigated to prevent recurrence and extend survival; these are, therefore, tumor types that can benefit from a better understanding of what an early diagnosis means for patients with these cancer types (31–44).

Studies were included if outcomes for patients with early stage and/or late stage cancer at the time of diagnosis were reported by disease stage. Patients could be eligible or not for chemotherapy, could present with comorbidities and may have been treated with or without surgical resection. The main outcomes of interest included: overall survival (OS), mortality, humanistic burden and financial impact. These outcomes had to be reported by stage at the time of diagnosis. A detailed summary of the predefined PICOTS criteria is provided in **Supplementary Appendix S2**.

### 2.3 Screening, selection, and data extraction

To identify relevant studies for inclusion, screening of titles and abstracts, followed by reviews of full-text articles, were undertaken by two independent reviewers. A third independent reviewer was involved to resolve any discrepancies. Data from the included studies were extracted into a pre-defined extraction form. The data extraction was conducted by two reviewers, and subsequently validated by a third reviewer.

### 2.4 Quality assessment

Quality assessment was conducted using the Newcastle-Ottawa Scale (observational studies) and the adapted Larg and Moss checklist (cost-of-illness studies) (45, 46). The Newcastle–Ottawa scale assesses studies based on three domains: the selection of the study groups, the comparability of the groups, and the ascertainment of the outcome of interest. With a total maximum score of nine, three threshold ranges are used to stratify identified studies in three levels: high quality (scores from 7-9), medium quality (scores from 4-6), and low quality (scores from 0-3) (46).

### 2.5 Data analysis and definitions

Identified studies reported various clinical (survival) outcomes such as OS, cancer-specific survival (CSS), disease-specific survival (DSS), disease-free survival (DFS), event-free survival (EFS), progression-free survival (PFS), mortality and others. In order to homogenize the presentation of results in this article, survival outcomes were limited to OS (median, and rate) and mortality. OS was chosen as it is the preferred measure of health technology bodies and regulators to assess the impact of treatment on patients’ clinical outcomes. Rates were defined as the proportion or percentage of patients who survived until a specific timepoint. Eligible studies measured OS from the time of diagnosis until death or end of study follow-up. Any study which measured OS from any other event (e.g. post-recurrence) was excluded. Only the data points that corresponded to a particular stage at diagnosis were considered for reporting purposes. In addition to reporting results by stage (I to IV) and substages when available (e.g. IIA-IIC), studies reporting results according to ‘T stage’ (based on the TNM staging system), reflecting tumor sizes, were also extracted. A summary of the definitions of early vs. late-stage cancer found in the identified studies is presented in **Supplementary Appendix S3**. Results were summarized narratively and comparative outcome data between early and advanced/late stages were shown wherever available.

## 3 Results

### 3.1 Study selection and PRISMA flow

**Figure 1:**
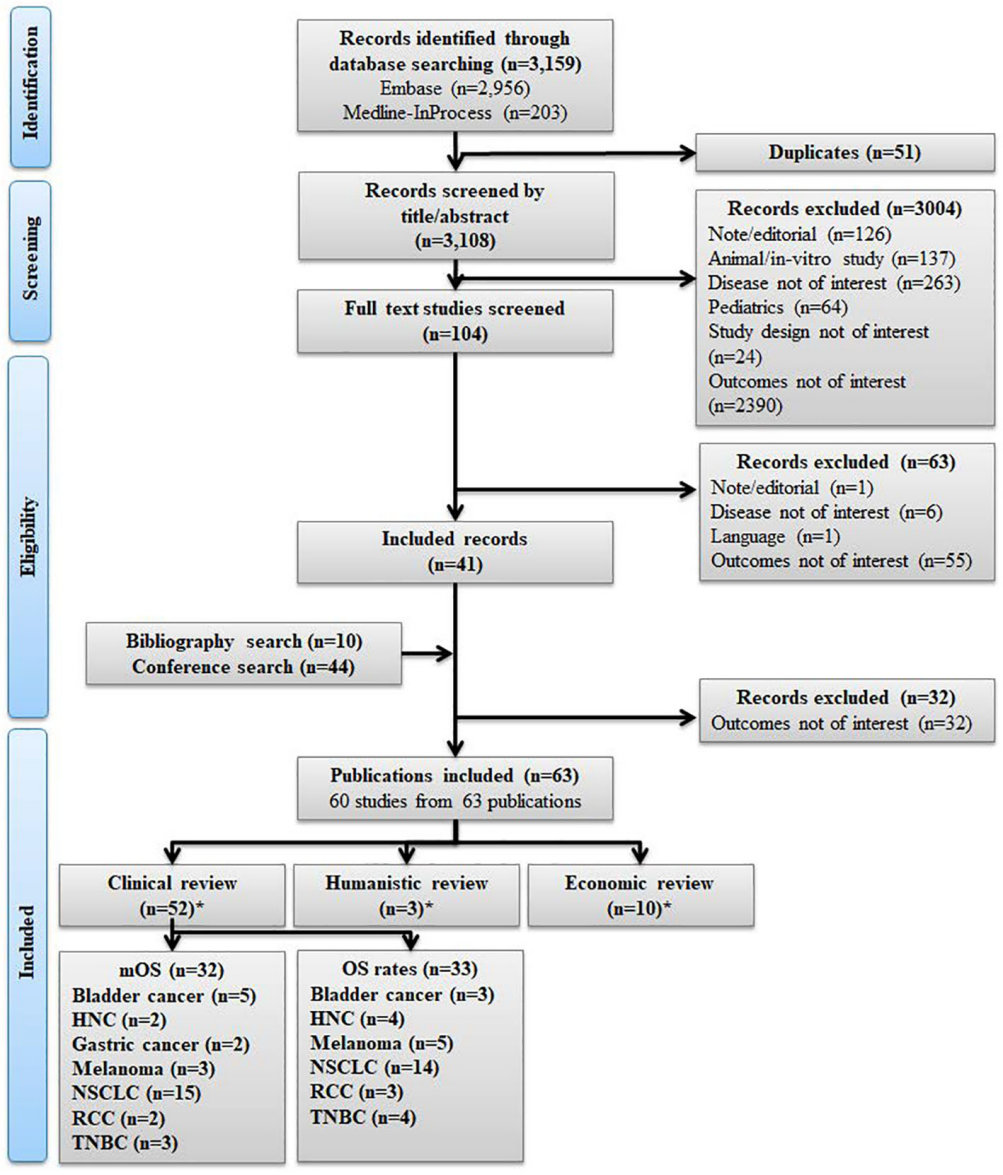
PRISMA flow diagram. *60 unique studies included across the clinical, humanistic, and economic reviews, where 32 unique studies were not included as the outcomes were not of interest; HNC, Head and neck cancer; mOS, Median overall survival; OS, Overall survival; PRISMA, Preferred Reporting Items for Systematic Reviews and Meta-Analyses. NSCLC, Non-small cell lung cancer; RCC, Renal cell carcinoma; TNBC, Triple negative breast cancer.

A total of 3,159 references were identified (**Figure 1**), of which 52 studies met the inclusion/exclusion criteria for the clinical review (**Supplementary Appendix S4**), three studies for the humanistic review (**Supplementary Appendix S5**), and 10 studies for the economic review (**Supplementary Appendix S6**).

#### 3.1.1 Summary of included studies

The majority of the 60 studies included in the clinical, humanistic, and economic review reported on NSCLC (26 studies, 43.3%), followed by HNC (7 studies, 11.7%) melanoma (7 studies, 11.7%), bladder cancer (8 studies, 13.3%), TNBC (7 studies, 11.7%), gastric cancer (2 studies, 3.3%) and RCC (3 studies, 5%).

Most studies were conducted in the US (27 studies, 45%), followed by Europe (16 studies, 26.7%), Brazil (4 studies, 6.7%), Iran (4 studies, 6.7%), Canada (2 studies, 3.3%), and multiple countries (2 studies, 3.3%). Other countries included (with one study conducted in each country) were: India, Taiwan, Uruguay, and Vietnam (**Supplementary Appendix S7**). One study did not report the country of analysis (47).

Most identified studies did not have an overarching classification or grouping to report early- versus late-stages of cancer. While a total of 27 studies provided definitions for early- and late-stage cancers (48–74), the remaining 76 studies included stage specific data but did not classify stages as early- or late-stage. In 10 studies, early stage comprised of stages I-II, while stages III-IV constituted advanced stage (51–53, 55–58, 64, 66, 74). Among the remaining studies, four studies defined stages I-IIIA in early stage and stages IIIB-IV in advanced stage, while three studies classified stages I to stage III under early stage. The studies that categorized specific sub-stages or the entirety of stage III as part of the early stage were primarily conducted in patients diagnosed either with NSCLC (six studies) (63, 65, 67–70) or TNBC (three studies) (71–73). Furthermore, one study defined early stage as “*in situ* carcinoma” and “localized” stages, while advanced stage was defined as “regional to lymph nodes”, “regional by direct extension”, and “distant” stages (49). The evidence from early and late stages was consolidated and presented after clearly indicating the classification of early and late stages and defining the criteria for each stage. Where defined, the terms “early” and “late” have been utilized in context of the identified studies summarized in **Supplementary Appendix S3**.

### 3.2 Clinical outcomes

A total of 52 studies reported survival outcomes, with 33 reporting information specific to OS (NSCLC: 23 studies (63, 64, 66, 67, 70, 75–92); HNC: 6 studies (54, 93–97); bladder cancer: 6 studies (48, 98–102); TNBC: 6 studies (74, 103–107); melanoma: 6 studies (47, 59, 60, 108–110); gastric cancer: 2 studies (111, 112); and RCC: 3 studies (113–115)).

In this section, studies that reported either median OS (mOS) and/or stage-specific 5-year OS rates, measured from diagnosis, are presented. A description of the studies reporting mOS or OS rates (measured at any time point reported, e.g., 1-year, 3-year, etc.) can be found in the **Supplementary Appendices S8, S9**, respectively.

Across all selected tumors, a general trend was observed for increased OS in patients diagnosed with earlier-stage disease (and less severe subgroup stages) compared to patients diagnosed at later stages (**Supplementary Appendix S8**). Further details of the findings per tumor type are reported below.

**Figure 2:**
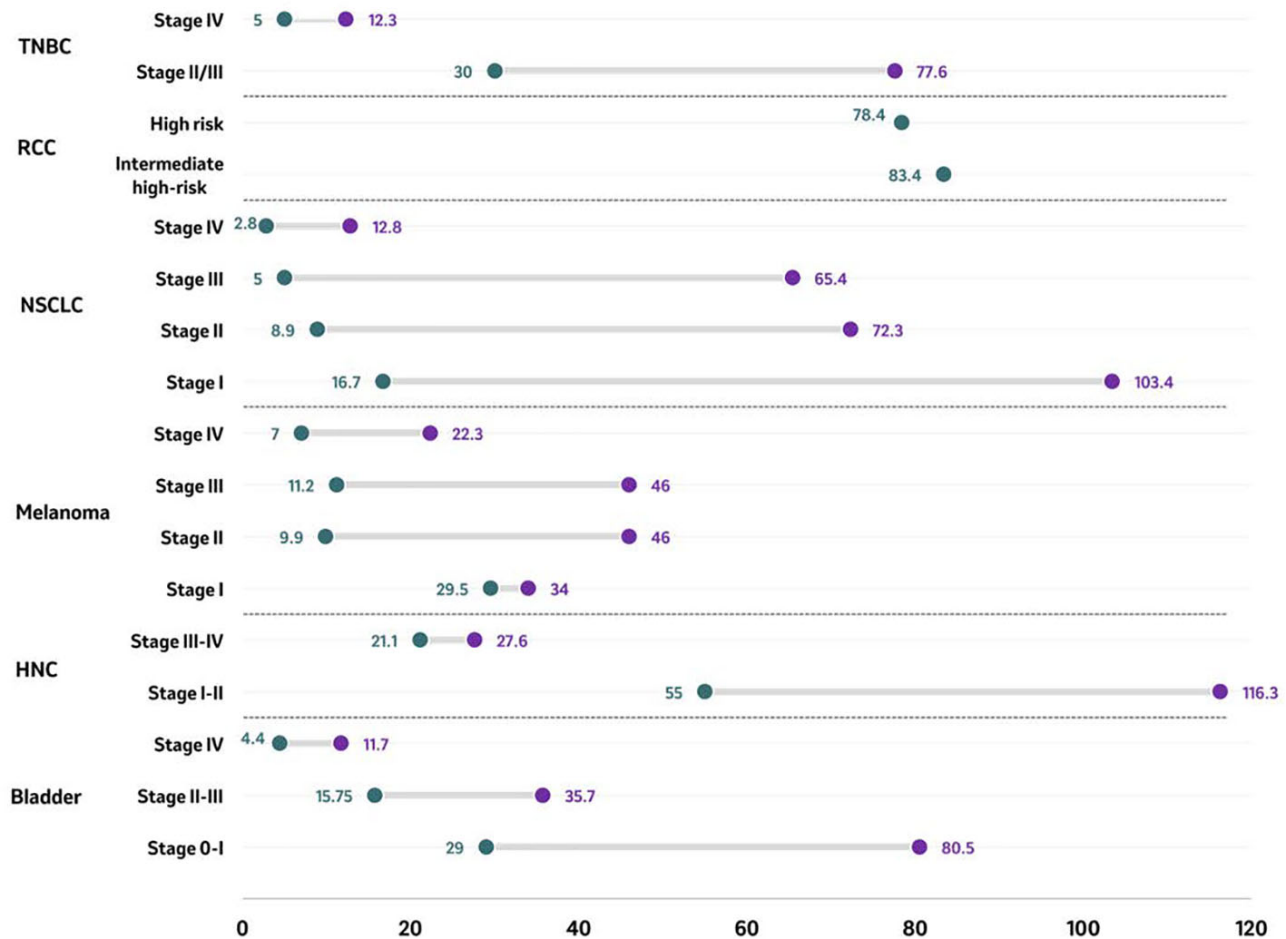
Median overall survival (in months) by tumor type according to stage at diagnosis. Median overall survival of reported stages in bladder cancer, HNC, melanoma, NSCLC, RCC, and TNBC are presented as ranges in months with the purple circle representing the upper end of the range and the green circle representing the lowest end of the range. For RCC, single data point per stage has been identified and reported. Source: Bladder ( (48, 98–101)); HNC ( (95, 96)); melanoma ( (59, 60, 108)); NSCLC ( (64, 70, 81–83, 85–92)); RCC ( (114, 115)); TNBC ( (74, 103, 104)). Studies included are those from which outcomes could be extracted. For studies including assessing mOR for patients with RCC (n=2) (114, 115), OS results were based on risk stratification rather than cancer staging, and no study on RCC reported stage-wise distribution of OS. HNC, Head and neck cancer; NSCLC, Non-small cell lung cancer; RCC, Renal cell carcinoma; TNBC, Triple-negative breast cancer.

Median overall survival (mOS) was reported across six tumor types, with most evidence identified for NSCLC [15 studies (64, 70, 79, 81–92)], followed by bladder cancer [5 studies (48, 98–101)], melanoma [3 studies (59, 60, 108)], TNBC [3 studies (74, 103, 104)], HNC [2 studies (95, 96)], and RCC [2 studies (114, 115)]. Overall, the longest mOS was observed in patients diagnosed with stage I-II HNC (116.3 months), while the shortest mOS was reported for patients diagnosed at stage IV NSCLC (2.8 months). Across tumor types in studies reporting multiple stages, mOS decreased with increasing stage at diagnosis (**Figure 2**). In patients diagnosed with stage II/III TNBC, mOS ranged between 30.0 months to 77.6 months compared with 5.0 months to 12.3 months in patients diagnosed with stage IV TNBC (74, 103, 104). Median OS was 5 months longer in patients diagnosed with intermediate-high risk RCC compared to those diagnosed with high risk RCC (83.4 months vs. 78.4 months, respectively) (114, 115). In patients diagnosed with NSCLC, the mOS ranged from 16.7 months to 103.4 months in patients diagnosed at stage I compared to a range of 2.8 months to 12.8 months in those diagnosed at stage IV (64, 70, 81–83, 85–92). Amongst all melanoma patients, the shortest mOS was reported for patients diagnosed with the most advanced stage, stage IV (5.1 months to 22.3 months), whereas the longest mOS was found in patients diagnosed in stage I (29.5 months to 34 months) (59, 60). In patients diagnosed with HNC, mOS was higher in patients diagnosed with localized disease (stage I-II: 55,0 months to 116.3 months) compared to those diagnosed at advanced disease (stage III-IV: 21.1 months to 27.6 months) (95, 96). In studies including patients diagnosed with bladder cancer, survival was longer for patients diagnosed at earlier stages (stage 0 and III) than for patients diagnosed at late stage (IV). The mOS for patients diagnosed in stage 0-I bladder cancer ranged from 29.0 months to 80.5 months compared with a range from 4.4 months to 11.7 months for patients diagnosed at stage IV bladder cancer (48, 98–101).

**Figure 3:**
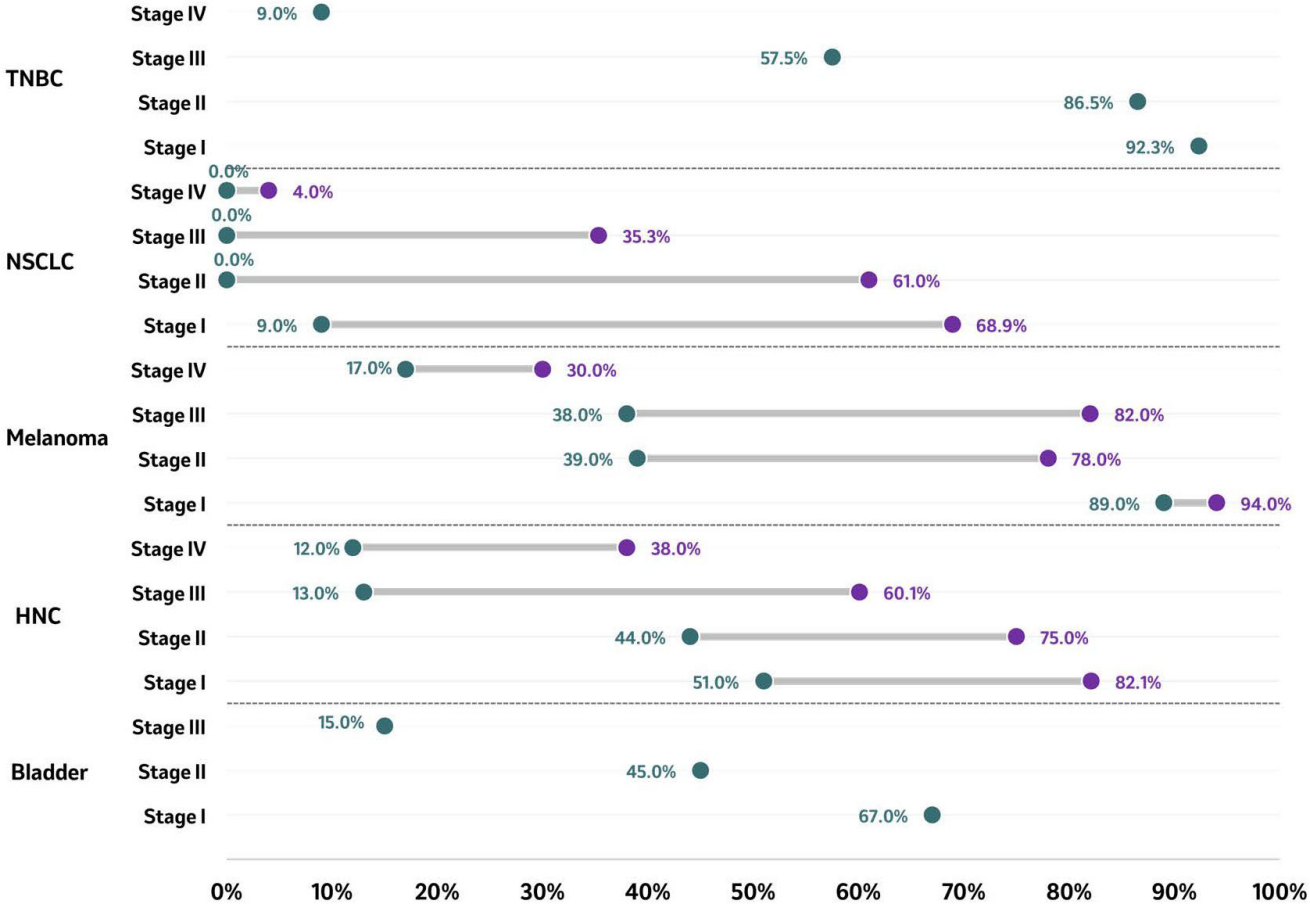
5-year OS rate by tumor type according to stage at diagnosis. Five-year survival rate of reported stages in bladder cancer, HNC, melanoma, NSCLC, RCC, and TNBC are presented as ranges in months, with the purple circle representing the higher end of the range and the green circle representing the lowest end of the range. For bladder cancer, a single data point per stage has been identified and reported. Source: Bladder ( (102)); HNC ( (54, 93, 94, 116)); melanoma ( (108)); NSCLC ( (63, 67, 70, 75, 76, 80, 89)); TNBC ( (74, 106, 107)). Studies included are those from which outcomes could be extracted. HNC. Head and neck cancer; NSCLC, Non-small cell lung cancer; TNBC, Triple-negative breast cancer.

The 5-year OS rate by stage was available across five tumor types, with the highest number of publications identified for NSCLC [8 studies (63, 67, 70, 75, 76, 78, 80, 89, 91)], followed by HNC [3 studies (54, 93, 94, 116)], TNBC [1 study (74, 105–107)], bladder cancer [1 study (102)], melanoma [1 study (108)] and RCC [2 studies (113, 114)]. Overall, the highest 5-year OS rate was observed in stage I melanoma (94%), and the lowest rate was reported in stage IV NSCLC (4%) (**Figure 3**) (63, 67, 70, 75, 76, 80, 89, 108). Across tumor types, the OS rate decreased with the progression of the disease stage at diagnosis. Patients diagnosed with TNBC reported a 5-year OS rate ranging from 92.3% in patients diagnosed at stage I to 9.0% in patients diagnosed at stage IV (74, 105–107). NSCLC 5-year OS upper end rates ranged between 69% in patients diagnosed with stage I and 4% among those diagnosed with stage IV. In patients diagnosed with melanoma stage I, the five-year OS rates were higher (89% - 94%) compared to patients diagnosed with stage IV melanoma (17% - 30%) (108). A similar pattern was observed in HNC, where the OS rate decreased from a range of 51% to 82% in patients diagnosed with stage I HNC to a range of 12% to 38% in those diagnosed at stage IV (54, 93, 94, 116). Similarly, the five-year OS rates in bladder cancer patients diagnosed at stage I, stage II, and stage III, were 67%, 45% and 15%, respectively (102), confirming the consistent downward trend identified across all tumor types with reported 5-year OS rates.

#### 3.2.1 Bladder cancer

Of the six studies reporting OS in bladder cancer, mOS was provided across five studies (48, 98–101), whereas OS rates 5 years post-diagnosis were provided in one study (102). The consensus on the extracted evidence showed a considerably higher mOS and OS rates for patients diagnosed at earlier stages compared to those diagnosed at more advanced/later stages (**Figures 2**, **3**).

In a study including patients diagnosed with urothelial (UC) and non-urothelial carcinoma (non-UC), survival was longer for earlier stages (stages 0&I, and II&III, respectively) than for late stage (IV). The mOS for patients diagnosed in early stage ranged from 35.7 months to 80.5 months in UC and from 15.8 months to 29.0 months in non-UC (98). In patients diagnosed at stage IV, the mOS was 8.6 months and 7 months in UC and non-UC, respectively (98). Four studies reported a mOS ranging from 4.4 months to 11.7 months in patients diagnosed with metastatic stages (**Figure 2**) (48, 99–101).

For OS rates (**Figure 3**), a similar trend was observed for mOS, where compared to those patients diagnosed early, patients diagnosed at a later stage experienced a decrease in OS rate. The five-year OS rates in patients diagnosed at stage I, stage II, and stage III, were 67%, 45% and 15%, respectively (p<0.001) (102). On the other hand, patients diagnosed at metastatic stages had shorter OS rates of 40.4% and 23.6%, at one and two-years post-diagnosis, respectively (101).

#### 3.2.2 Gastric cancer

Of the two included studies that reported OS in gastric cancer, two reported mOS (111, 112), and none reported OS rate 5 years post-diagnosis. Across these studies, in general, early diagnosis in gastric cancer led to better OS outcomes. In patients with gastric cancer, the mOS was 37.0 months in those without metastasis at the time of diagnosis compared to 14.0 months among patients diagnosed with metastatic gastric cancer (112). The shorter mOS observed amongst patients diagnosed with metastatic disease was highlighted in another study, where the mOS was 5.5 months among patients diagnosed with potentially curable gastric or gastroesophageal junction adenocarcinoma following diagnosis of interval metastases (111).

#### 3.2.3 Head and neck cancer

Of the six included studies that reported mOS and OS rates, two reported mOS (95, 96), whereas three included OS rates 5 years post-diagnosis (54, 93, 94). These studies reported improved OS outcomes among patients diagnosed at an earlier stage compared to those diagnosed at later stages.

A significantly higher mOS of 84.1 months was reported for patients diagnosed with laryngeal cancer at localized disease (stage I–II) compared to 24.1 months among those diagnosed with advanced stage laryngeal cancer (stage III-IV) (**Figure 2**) (95). A similar mOS of 22.6 months was reported in another study that included patients diagnosed with stage III or IV, M0 hypopharyngeal squamous cell carcinoma (96).

With respect to OS rates, a trend for improved OS was observed among patients diagnosed with HNC at an earlier stage compared to patients diagnosed at later stages (**Figure 3**). In a study including patients diagnosed with oral cancer, the 5-year OS rates were 83.9%, 82.1%, 72.7%, 60.1% and 38.0% for those diagnosed with stage 0, I, II, III, and IV, respectively (54). Among patients diagnosed with lip cancer, the 5-year OS rates were 81% for those diagnosed in stage I, 75% for those diagnosed in stage II, and 45% for those diagnosed in stage III (93). Among patients diagnosed with oral cancer, the 5-year OS was 51% when diagnosed in stage I, 44% when diagnosed in stage II, 13% when diagnosed in stage III, and 12% when diagnosed in stage IV (94).

#### 3.2.4 Melanoma

Of the six studies that reported OS among patients diagnosed with melanoma according to stage at the time of diagnosis, three provided the mOS (59, 60, 108), and one reported OS rate 5 years post-diagnosis (108). Patients diagnosed with early-stage melanoma survived longer in general than those diagnosed in late stages. Patients diagnosed with melanoma had a mOS of 46 months when diagnosed at stage IIC, 36 months when diagnosed at stage IIIC, and 9 months when diagnosed at stage IV (**Figure 2**) (108). Contrasting results were found in another study with a lower mOS among patients diagnosed at stage IIC (9.9 months) compared to patients diagnosed at stages IIIA, IIIB and IIIC (15.7, 15.5 and 11.2 months, respectively) (59). In patients diagnosed with unresectable stages IIIB/IIIC and IV M1a melanoma, those with stage IIIB/IIIC at diagnosis had a mOS of 24.3 months, compared to 22.3 months when diagnosed at stage M1a, 11.2 months when diagnosed at stage M1b, and 5.1 months when diagnosed at stage M1c (60).

With regards to OS rates, the included study showed improved survival outcomes for patients diagnosed at earlier stages compared to those diagnosed at more advanced stages (**Figure 3**). In a cohort of patients diagnosed with malignant melanoma, the 5-year OS rate was 94% among patients diagnosed with melanoma at stage IA, 90% when diagnosed at stage IB, 78% when diagnosed at stage IIA, 64% when diagnosed at stage IIB, 39% when diagnosed at stage IIC, 79% when diagnosed at stage IIIA, 57% when diagnosed at stage IIIB, 38% when diagnosed at stage IIIC, and 20% when diagnosed at stage IV (108). There is again a similar trend with patients with stage IIC melanoma having worse survival outcomes than those with stage IIIA and IIIB disease, despite the absence of nodal disease (108).

#### 3.2.5 Non-small cell lung cancer

In NSCLC, 23 studies reported data on OS, with 15 reporting mOS (64, 70, 81–83, 85–92) and 8 providing OS rates (63, 70, 75, 76, 78, 80, 89, 91) 5 years post-diagnosis for patients diagnosed with NSCLC by stage at diagnosis. The trend across these studies indicated that patients diagnosed at earlier stages of NSCLC had better survival outcomes than those diagnosed in advanced stages. Longer survival times were also observed in patients with non-squamous NSCLC compared to squamous NSCLC.

Across the included studies, the reported mOS ranged from 16.7 months to 103.4 months for patients diagnosed with NSCLC at stage I, 8.9 months to 72.3 months for those diagnosed at stage II, 5.0 months to 65.4 months for those diagnosed at stage III, 2.8 months to 12.8 months for those diagnosed at stage IV (**Figure 2**) (64, 70, 81–83, 85–92). For non-squamous and squamous histologies, respectively, the mOS was 43.2 months and 23.6 months for patients diagnosed at stage II, 26.7 and 20.4 months when diagnosed at stage IIIA, 12.9 and 12.5 months when diagnosed at stage IIIB, and 7.6 and 6.1 months when diagnosed at stage IV, respectively (82). Another study showed longer mOS for stage I NSCLC patients with non-squamous histology (range mOS: 55.3 months to not reached) compared to those with squamous NSCLC (range mOS: 37.3-51.1 months). In contrast, patients diagnosed with stage IIIA non-squamous NSCLC had shorter mOS, ranging from 9.9 to 24 months (70).

OS rates were also lower among patients diagnosed with NSCLC in advanced stages compared to early stages (**Figure 3**). At 5 years post-diagnosis, OS rates ranged from 9% to 69% for patients diagnosed at stage I, from 0% to 61% in stage II, from 0% to 35% in stage III, and from 0% to 4% for those diagnosed at stage IV (**Figure 3**) (63, 70, 73, 76, 78, 80). At 5 years post-diagnosis, the mortality rate was higher in patients diagnosed at stage II than those diagnosed at stage I (63.8% vs 45%) (76). In patients diagnosed at advanced stages (III-IV), after 13.2 months of follow-up, the death rate ranged from 28% to 54% (76, 78, 117). In terms of tumor size, increased size at the time of diagnosis, such as in T1-T4, showed reduced 5-year OS rates (12.7%-13.5%) compared to T0 sizes (30.5%-35.3%) (75).

#### 3.2.6 Renal cell carcinoma

One of three included studies that were identified in RCC reported the mOS among patients diagnosed in RCC by stage, and two reported the OS rates 5 years post-diagnosis. Longer survival times were observed among patients with RCC diagnosed at an earlier stage compared to those diagnosed at later stages. Among patients diagnosed with non-metastatic stages, the mOS was 83.4 months for patients diagnosed with intermediate-high RCC (pT2N0 high grade, pT3N0), compared to 78.4 months for patients diagnosed with high risk RCC (pT4N0, pTanyN1) (**Figure 2**) (114, 115).

In terms of OS rates, findings showed that the higher the disease grade, the lower the proportion of patients who survived. At 5 years post-diagnosis, the OS rate was 37% for patients diagnosed with T3G4, 65% for patients diagnosed with T3G3, and 77% for patients diagnosed with T3G1-G2 (114, 115). Across stages and in patients of White and Asian race, the 5-year OS ranged from 90.3% to 91.9% in patients diagnosed with localized clear-cell RCC, from 70% to 71.5% in those diagnosed with regional ccRCC, and from 20.3% to 34.1% in patients diagnosed with distant ccRCC (113).

#### 3.2.7 Triple negative breast cancer

Of the six studies that were identified in TNBC, three specifically examined mOS, while one reported OS rates 5 years post-diagnosis. There was a notable decrease in survival in patients with a late-stage diagnosis compared to those diagnosed at an early stage.

In patients who initiated systemic neoadjuvant and/or adjuvant therapy, the median OS was 77.6 months when diagnosed in stage II and ranged from 30.0 months to 37.8 months when diagnosed at stage III (**Figure 2**) (92). In patients diagnosed with TNBC in advanced stages (III or IV), the median OS was 18.0 months (95% CI: 16.0, 20.0) across all patients, yet only 5.0 months (95% CI: 4.0, 7.0) among patients diagnosed with stage IV TNBC (n=416) (74). These findings were consistent with those of another study, which reported a median OS of 7.0 months (95% CI: 6.2, 8.1) in elderly patients (≥66 years) with newly diagnosed metastatic disease (104).

OS rates showed reduced survival as tumor stage advanced (**Figure 3**) (74, 106, 107). At 5 years post-diagnosis, the OS rates were 92.3%, 86.5%, 57.8% and 9.0% in patients diagnosed with stage I, II, III, and IV TNBC, respectively (105).

### 3.3 Humanistic burden

Three studies, including patients diagnosed with bladder, melanoma, and NSCLC, reported HRQoL outcomes for patients diagnosed in either early or advanced stages of cancer (58, 118, 119).

In bladder cancer, a study conducted included patients diagnosed with non-muscle invasive bladder cancer (NMIBC) and muscle invasive bladder cancer (MIBC) showed better quality of life, in terms of higher EORTC QLQ-C30 physical functioning, role functioning, cognitive functioning, emotional functioning and social functioning scales, among patients diagnosed with NMIBC compared to those diagnosed with MIBC. Scores on these domains for patients with NMIBC vs. MIBC were 84 vs. 79, 83 vs. 72, and 76 vs. 75, 84 vs. 81, and 86 vs. 81, respectively. Among patients diagnosed with NMIBC and MIBC, fatigue and insomnia had the highest scores in the EORTC QLQ-C30 symptom scale. Patients diagnosed with NMIBC reported significantly better results for the role functioning domain (83; SD: 28) vs. those diagnosed with MIBC (72; SD: 34; p<0.001) (118). In terms of the physical functioning scale of the EORTC-QLQ-C30 for non-muscle-invasive and muscle-invasive bladder cancer, patients diagnosed with advanced stage disease reported worse HRQoL when compared to those diagnosed at earlier stages. Clinically relevant differences of more than 10 points were observed between patients with invasive and non-invasive tumors, with physical/mental health domain scores of 69/71 for patients diagnosed with pT4 disease and 79/81 for patients diagnosed with non-invasive tumor stage pT1 or below (118).

Two other studies examined the HRQoL among patients with melanoma and NSCLC but were limited in the examination of stage-specific humanistic outcomes and are thus briefly discussed here. Across patients diagnosed with stage I or II melanoma, HRQoL improved over time for emotional functioning and worsened for physical functioning. Among patients diagnosed with stage IV NSCLC, the most relevant cancer-related symptoms experienced included tiredness (84.1%), low well-being (80.7%), low appetite (71.7%), and shortness of breath (67.8%) (58, 119).

### 3.4 Economic burden

Of the 10 included studies reporting outcomes related to the economic burden at different stages of cancer diagnosis, 8 studies reported on the financial impact, and 5 studies reported healthcare resource utilization (HCRU). Eight included studies were conducted in the US, one in Denmark and one in India. Among the included studies, 2 evaluated patients with bladder cancer, 1 focused on patients diagnosed with HNC, 3 investigated patients diagnosed with NSCLC and 4 assessed patients diagnosed with TNBC.

**Figure 4:**
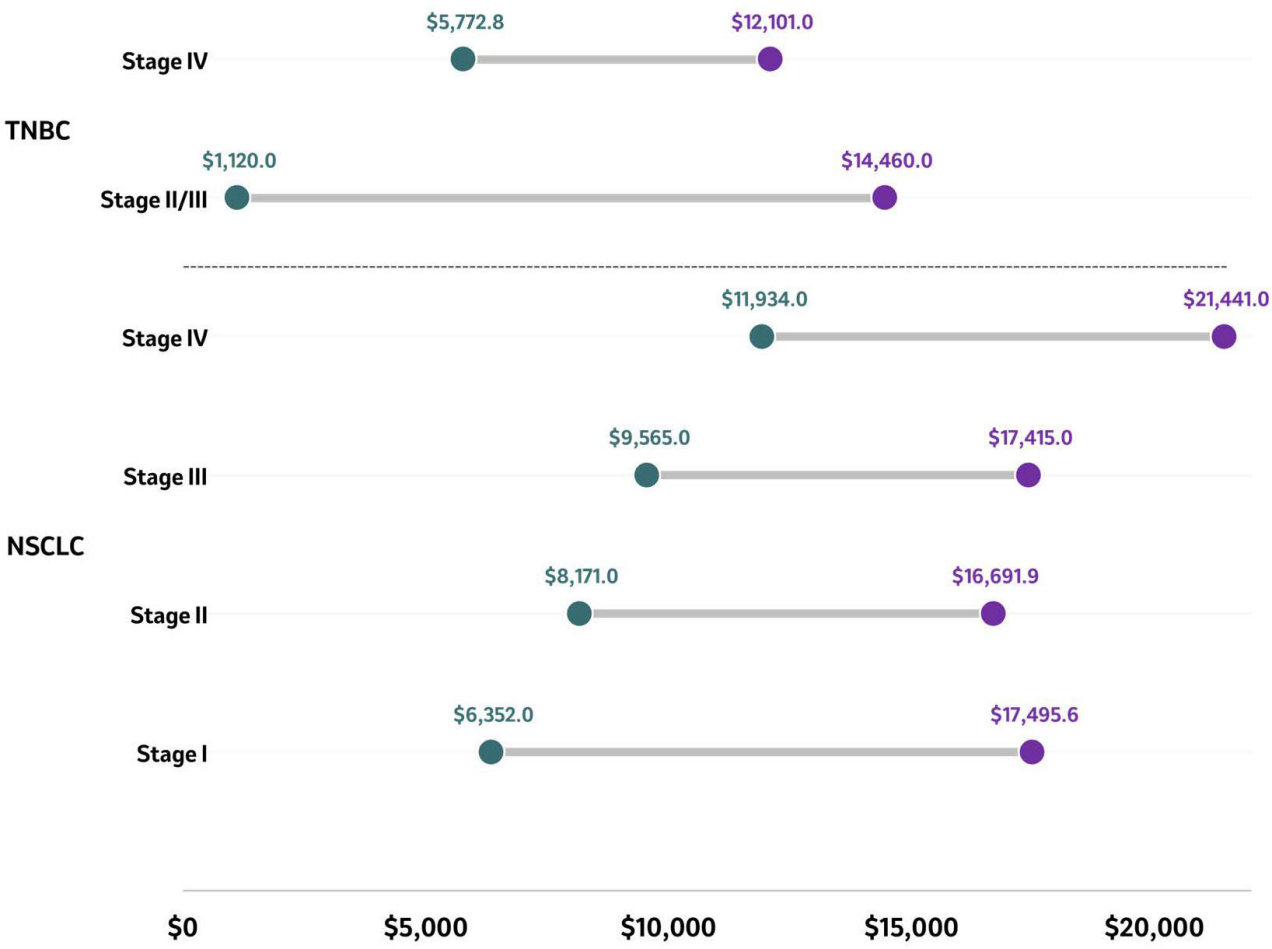
Average PPPM costs in patients with TNBC and NSCLC by stage. Average PPPM costs of reported stages in NSCLC and TNBC are presented as ranges in US$ with the purple circle representing the higher end of the range and the green circle representing the lower end of the range. Source: NSCLC ( (65, 77, 117)); TNBC ( (74, 103, 120)). NSCLC, Non-small cell lung cancer; PPPM, Per patient per month; TNBC, Triple negative breast cancer.

Three studies reported the financial impact of patients diagnosed with TNBC at different stages, with a lower impact reported for those diagnosed in earlier stages compared to more advanced stages (**Figure 4**) (74, 103, 120). Across the studies, in patients diagnosed with early stage TNBC (II/III), the total monthly costs per patient ranged from US$1,120 to US$14,466, and among patients diagnosed with stage IV TNBC, costs ranged from US$5,773 to US$12,101 (74, 103, 120). In early-stage TNBC (II, IIIA, IIIB), outpatient treatment was the main driver of the total treatment cost, while hospitalizations were uncommon, with outpatient costs of adjuvant therapy of US$24,408 and neoadjuvant therapy costs of US$10,620 (103).

Overall, three studies reporting HCRU in patients diagnosed with TNBC were included (71, 74, 104). Among elderly patients diagnosed with metastatic TNBC, and who were treated with chemotherapy, the median interquartile range (IQR) for office visits was 5 (3-8). A total of 66.2% of patients were hospitalized, although this was more prevalent in those who received at least three lines of therapy (104). In patients with stage II-IIIB disease at diagnosis who had received either neoadjuvant with or without adjuvant therapy, 25.8% to 36.1% were hospitalized and the average length of stay ranged from 0.34 to 1.0 days (71). Among elderly patients diagnosed with stage III or IV TNBC, healthcare resource use varied at three different time points (the first 3 months after diagnosis, the last 3 months of life, and the time in between, called intervening), when comparing stage III to stage IV in terms of the average number of hospitalization (0.6 vs. 0.6 in initial quarter, 1.5 vs. 1.3 in intervening period, and 0.9 vs. 1.1 in last quarter), outpatient visits (5.4 vs. 5.1 in the initial quarter, 23.8 vs. 20.6 in the intervening period, and 2.4 vs. 2.6 in the last quarter), and hospice claims (0.0 vs. 0.1 in initial quarter, 0.3 vs. 1.1 in intervening quarter, and 0.5 vs. 0.6 in the last quarter) (74).

Three studies were included that reported the financial impact of patients diagnosed with NSCLC at different stages (**Figure 4**) (65, 77, 117). Across studies, the per patient per month (PPPM) cost was highest among patients diagnosed with stage IV ($21,441) and lowest among those diagnosed with stage I ($6,352) disease (65, 77, 117). A study conducted in the US and evaluating adult patients diagnosed with stage IB to IIIA NSCLC (n=609) was performed using the Vector Oncology Data Warehouse electronic medical record and billing data collected between 2007 and 2014 (65). During adjuvant treatment, the total monthly median cost per patient was US$17,389.75 (IQR: US$8,815.61, US$23,360.85). During adjuvant treatment, the median total cost was US$17,495.64 for stage IB compared to US$19,178.60 for stage IIA/II, while patients diagnosed at later stages had a median total cost of US$17,784.05 for stage IIB and US$13,659.36 for stage IIIA (65). A study conducted in the US based on a proprietary oncology registry linked to health insurance claims from a large US health insurance company assessed the costs of treating adult patients diagnosed with NSCLC between 2007 and 2011 based on the stage at diagnosis (n=1,210) (117). The PPPM mean total health care costs and utilization after lung cancer diagnosis were US$7,239 at stage I, US$9,484 at stage II, US$11,193 at stage IIIA, US$17,415 at stage IIIB, and US$21,441 at stage IV. The PPPM average total health care costs and utilization were the highest among patients diagnosed with stage IV (US$21,441) and the lowest among those diagnosed with stage I ($7,239) disease (**Figure 4**) (117). Similar findings were reported in a study conducted in the US based on SEER-Medicare data that included patients diagnosed with stage I-IV NSCLC between 2006 and 2015 (77). In both treated and untreated patients, the stage at diagnosis was associated with increased healthcare expenditures. The predicted mean healthcare expenditure per month increased continuously with advancing stage at diagnosis. The mean monthly expenditure was US$6,352, US$7,731, US$8,171, US$9,396, US$9,565, US$10,614, and US$11,934 among patients diagnosed with NSCLC at stages IA, IB, IIA, IIB, IIIA, IIIB, and IV, respectively (p<0.001 across stages IB to IV vs. stage IA) (**Figure 4**) (77).

Two studies reported HCRU in patients diagnosed with NSCLC (65, 117). In a study assessing patients aged ≥18 years diagnosed with stage IB to IIIA NSCLC (n=609), HCRU (office visits and incidence/duration of hospitalization) did not differ significantly across groups diagnosed at different disease stages. The average number of hospitalizations per patient during adjuvant therapy was 0.38 for stage IB, 0.06 for stage IIA/II, 0.23 for stage IIB and 0.31 for stage IIIA. However, the duration of hospitalization did not significantly differ across groups diagnosed at different stages of disease (p=0.128) (65). Another study in which HRCU rates were compared at early and late diagnosed stages, found lower rates of HCRU after diagnosis (117). The percentage of patients with stage I NSCLC requiring an office or outpatient visit was respectively 29.7% and 18.2% compared to 48.8% and 33.9% for patients diagnosed with stage IV NSCLC (117).

**Figure 5:**
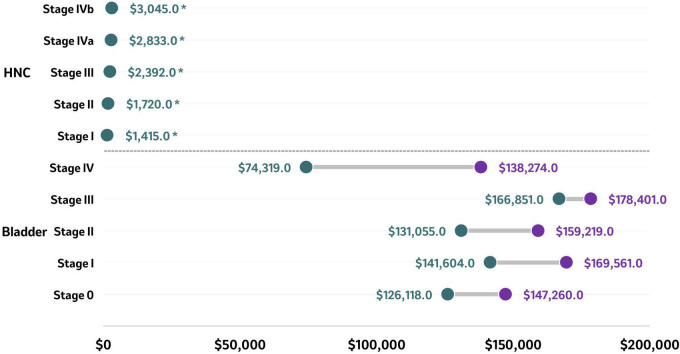
Total costs at 6 months (HNC) and lifetime costs (bladder) according to stage at diagnosis. Total costs of reported stages in bladder cancer (lifetime costs) and HNC (at 6 months) are presented as ranges in US$ with the purple circle representing the higher end of the range and the green circle representing the lower end of the range. Source (55, 121):. *Study conducted in India hence the low total cost values. HNC, Head and neck cancer.

A study conducted at public hospitals in India reported the treatment costs between 2019 and 2020 for patients diagnosed with HNC (oral cancer) (n=100) (55). The unit cost of treating patients with oral cancer diagnosed in advanced stages was US$3,045 (stage IVb), twice that of the cost of treating patients diagnosed with oral cancer in early stages (US$1,415 for stage I), demonstrating that the more advanced the cancer stage, the higher the cost (**Figure 5**). This finding was observed across all cost categories, including capital costs, which were five times higher for patients diagnosed in stage IVB (US$686) compared to stage I (US$140), while personnel costs and variable costs were almost twice as high in patients diagnosed with more advanced stages compared to those diagnosed in earlier stages (personnel costs: US$891 in stage I vs. US$1,545 in stage IVB); variable costs: US$384 in stage I vs US$815 in stage IVB) (55). From a HCRU perspective, surgery constituted 30% of the overall costs, followed by inpatient costs (19%) and diagnostics (17%) among patients diagnosed with early stage HNC. In patients diagnosed in advanced stage HNC and treated with radiotherapy (RT), surgery constituted 26% of overall costs, whereas RT and inpatient services amounted to 30%, and 15% of the overall costs. When treated using chemoradiotherapy, the adjuvant treatment, surgery and diagnostics (17%) made up the bulk of the overall costs (55).

Only one study reported the financial impact of bladder cancer per stage at the time of diagnosis (121). Based on SEER-Medicare database records between 2004 and 2013, the reported mean lifetime costs of managing patients ≥66 years newly diagnosed with urothelial carcinoma (n=15,558) were higher among patients diagnosed with bladder cancer stage III compared to patients diagnosed at earlier stages (**Figure 5**) (121). Lifetime costs were US$126,118 - $147,260 for stage 0, US$141,604 - $169,561 for stage I, US$131,055 - $159,219 for stage II, US$166,851 - $178,401 for stage III, and US$74,319 - $138,274 for stage IV. Hospitalizations unrelated to cystectomy contributed 48% to 53% of these lifetime costs, averaging US$73,903 at stage 0, US$73,249 at stage I, US$72,709 at stage II, US$100,356 at stage III, and US$59,494 at stage IV. Cystectomy contributed 2% to 13% of the lifetime costs, averaging US$3,356, US$7,011, US$11,855, US$25,509, and US$11,693 for stages 0, I, II, III, and IV, respectively. Urothelial carcinoma-related office visits contributed 8% to 15% of the lifetime costs, totaling US$11,717 at stage 0, US$14,611 at stage I, US$19,882 at stage II, US$21,480 at stage III, and US$17,820 at stage IV (121).

A Danish study reported HCRU in patients diagnosed with stage IV urothelial carcinoma between 2013 and 2017 (n=620). The mean number of hospital admissions, outpatient visits, and emergency department visits per patient per year (PPPY) was 7.6 (SD: 7.3), 26.6 (SD: 24.4), and 3 (SD: 3.5), respectively (101).

### 3.5 Risk of bias

According to the quality assessment based on the Newcastle-Ottawa scale, the majority of observational studies included in this SLR had a total score of 4 to 6, indicating the studies are of medium quality (n=48) and have a high risk of bias. None of the studies had a total score ≥7 and high quality, while 11 were low quality studies, with a total score of 1 to 3. One study was excluded from assessment due to insufficient data available from the conference abstract. When stratified by tumor type, 42% and 50% of studies identified under melanoma and gastric cancer were of low quality. The main weaknesses of the studies that resulted in lower quality scores were related to selection of non-exposed cohort (n=11), in particular the non-exposed cohort was either drawn from a source other than the exposed cohort or the source for non-exposed cohort was not described. All studies scored low on the comparability domain as the cohorts included were not comparable on the basis of design or the analysis was not controlled for confounders (n=11). Another weakness across the included studies was the lack of follow-up details among the cohorts in the publication (n=10).

Only one cost of illness study was identified in the SLR. According to the Larg and Moss checklist, the overall quality of the study was good. The main weakness of this study was that healthcare resources were not valuated, and there was unclarity on the plausibility of occurrence of a counterfactual population, as well as on the approach for valuing production losses, point estimates, and key assumptions for the sensitivity analyses.

The results of the quality assessments are presented in **Supplementary Appendix S10**.

## 4 Discussion

The findings of this SLR indicated that diagnosing cancer at an earlier stage was associated with improved long-term OS, improved HRQoL and reduced healthcare utilization and associated costs. Whilst there has been a growing body of evidence supporting the early diagnosis of cancer, literature reviews conducted to date have been limited or have had a narrow focus, to only include single cancer sites or specific malignant conditions. This study provides a detailed overview of the broad reaching implications associated with early cancer diagnosis across seven different tumor types, outlining the clinical, humanistic, and economic benefits associated with an early diagnosis when compared to late diagnosis. As such, the approach adopted, accounting for a broad range of tumor types along with the range of outcomes summarized, represents a novel and valuable addition to the current literature.

### 4.1 Clinical benefits associated with early diagnosis

The majority of the identified studies, across the tumor types included in the SLR, indicated that cancer patients diagnosed with late-stage disease were reported to have worse mOS and five-year survival rates when compared to patients diagnosed at an earlier stage. The largest decrease in mOS was observed for NSCLC, decreasing by a factor of 8.1 from 103.4 months at the highest end of the range at stage I, to 12.8 months at stage IV (**Figure 2**). A similar trend was identified when comparing the highest end of the mOS range between early and late-stage diagnosis, with a decrease by a factor of 6.9, 4.2, 2.1, 1.1, and 6.3 for bladder cancer, HNC, melanoma, RCC, and TNBC, respectively. The same trend was also observed for 5-year survival rates which decreased between early and late stage diagnosis by a factor of 4.5, 2.1, 3.1, 17.2, and 1.1 in bladder cancer, HNC, melanoma, NSCLC, and TNBC, respectively (**Figure 3**). This is consistent with the evidence found in the literature and reinforces the need to implement early diagnosis practices.

In bladder cancer, a trend analysis of SEER data from 148,315 patients diagnosed between 1973 and 2009 showed that stage-specific five-year survival rates were higher in patients diagnosed at localized and regional stages compared to distant stages (20). The five-year relative survival rates ranged from 82.7%-91.5% in patients diagnosed with localized stage cancer. In patients diagnosed with regional stage cancer survival rates ranged from 38.2% to 50.1%, and in patients diagnosed with distant stage disease it was 10.2%. In cutaneous melanoma, a systematic review found a similar result, in which OS decreased with increasing stage with a 5-year OS of 95%–100% in stage I, 65%–92.8% in stage II, 41%–71% in stage III, and 9%–28% in stage IV (21). In HNC (tonsil, tongue, and oral cavity cancers), the 5-year relative survival rates (between 2002-2006) decreased in patients diagnosed with later stage disease. In patient diagnosed with local stage OS ranged from 82.8%-85.9%, in patient diagnosed with locally advanced disease OS ranged from 49.8% to 73.0%, and in those diagnosed with distant stages, OS ranged from 29.5% to 41.5% (22).

The existing body of evidence supports a consistent pattern regarding the relationship between cancer stage at diagnosis and patient survival. This clear trend across multiple tumor types, demonstrates that an earlier cancer diagnosis can positively influence survival outcomes.

### 4.2 Humanistic benefits associated with early diagnosis

The SLR identified limited data relating to HRQoL and the humanistic outcomes associated with a diagnosis of cancer at an earlier or later stage, with the evidence restricted to three studies that included patients with melanoma, NSCLC and bladder cancer. Within these studies, analyses of HRQoL by stage of cancer at diagnosis were minimal, with only one comparison between NMIBC and MIBC in terms of quality of life being available.

No evidence pertaining to HRQoL according to the cancer stage at the time of diagnosis was identified for TNBC, RCC, gastric cancer, and HNC. The humanistic burden among patients with TNBC has been described in a previous SLR which reported the quality of life of patients with invasive or metastatic TNBC, or early-stage androgen receptor-positive TNBC, following different treatment options. However, findings were not categorized according to cancer stage at diagnosis (73). In a Canadian study, consisting of 29 interviews with patients suffering from advanced melanoma (stage III and IV), patients noted greatly diminished overall functioning and quality of life by the time they reached advanced stage disease (24).

The humanistic impact of cancer stage at the time of diagnosis remains poorly characterized with scarce evidence. Further investigation into the humanistic benefits of early diagnosis across various cancer types is needed, particularly for those tumor types where evidence is lacking.

### 4.3 Economic benefits associated with early diagnosis

Limited evidence was identified for outcomes relating to healthcare utilization and costs associated with stage of cancer at the time of diagnosis. 10 studies were identified that assessed patients with bladder cancer, HNC, NSCLC and TNBC. In these studies, the results indicated that overall, patients diagnosed at an earlier stage incurred lower healthcare resource utilization and costs compared to patients diagnosed at a later, advanced, or metastatic stage.

The economic burden associated with cancer is substantial and has been well documented with estimates varying, depending on the tumor type and stage at diagnosis. The factors contributing to increasing costs in advanced stage cancer at the time of diagnosis were predominantly: higher management costs (77), the need for subsequent lines of treatment (121), greater number of monthly claims of hospice care (74), hospitalizations (104), greater number of office and outpatient visits (74, 117), and subsequent recurrence and related hospitalizations (65).

A previously published SLR evaluating the cost burden associated with advanced NSCLC in Europe and the influence of disease stage concluded that, despite the relative paucity of data on the financial burden incurred directly by patients and caregivers, the financial burden of advanced NSCLC was considerable, with both direct and indirect costs increasing as disease progresses (26). Furthermore, in an SLR focused on patients with TNBC, healthcare costs and resource utilization were shown to increase significantly with disease recurrence, progression, and increased cancer stage, as well as line of therapy (73). Whilst these previously published findings are generally consistent with those of this SLR, the body of evidence is limited, and no study has comprehensively characterized the economic burden associated with different tumor types and stage of cancer at diagnosis or evaluated the benefits of early diagnosis. Earlier cancer diagnosis may not only improve patient outcomes, but may also help offset healthcare costs associated with more advanced stages of disease (27). The economic benefits associated with early diagnosis remain an essential area for further research. In particular, investigations focusing on cancer types beyond bladder cancer, HNC, NSCLC, and TNBC would be valuable to confirm this study’s findings and the economic benefits of early diagnosis.

### 4.4 Implications for clinical practice and research

To maximize the benefits associated with early diagnosis, regular screening for early identification of cancer, and early treatment once a patient has been diagnosed with cancer, should be prioritized by policy makers, healthcare providers and clinicians alike, by means of a cohesive and multidisciplinary approach among all stakeholders, aiming to increase awareness among patients around the importance and implications of early diagnosis.

Further research into early detection methods and the use of diagnostic biomarkers to improve early diagnosis among cancer patients remains a key priority. Intensive efforts into biomarker discovery and validation are ongoing, hoping to provide more sensitive and specific diagnostic testing and screening, such as multi-cancer early detection tests (122). At the same time imaging technology is evolving rapidly, with AI integration for enhanced early detection that may represent a key opportunity to improve the efficiency of early cancer detection (123). Advances in molecular profiling are also being leveraged to provide deeper insights into genetic and epigenetic changes in early-stage cancers. Meanwhile, liquid biopsy research is advancing non-invasive detection methods using bodily fluids, which may have implications for future screening programs, facilitating early detection. Each of these areas of research represent an opportunity to improve the precision and timeliness of cancer diagnosis for patients, resulting in improved patient outcomes in the future.

### 4.5 Strengths and limitations

The major strength of this SLR was to provide a pan-tumor, holistic account of the real-world benefits of early diagnosis and the impact on the clinical, humanistic, and economic outcomes. This study provides a comprehensive summary of all evidence available on the listed databases, from conception up to the year 2022, and is to the best of our knowledge the only SLR presenting a broad overview of the benefits of early diagnosis across multiple tumor types. A main limitation was the curtailment of survival metrics to assess overall survival with other metrics that included; disease specific survival, relative survival, actuarial survival, or cumulative survival. However, their prevalence across relevant studies was low. This would have introduced a certain degree of heterogeneity which would have rendered the synthesis and interpretation of results difficult. Moreover, the interpretation of results may be influenced by the varying definitions of early and late-stage disease across different studies, mainly affecting the comparability of outcomes. Therefore, when interpreting these results, readers should consider the specific definitions used in each study and exercise caution when making broad generalizations across the field. It should be noted that the interpretation of the study findings may be subject to ecological fallacy. The overall trend shows that diagnosing in earlier stages leads to improved outcomes, but this may overlook individual variability in survival outcomes. In individual cases, late diagnosed patients with particular idiosyncrasies (cancer type, individual health status, and treatment response) may have better outcomes. Likewise, the availability and access to treatment may entail different benefit trends between early and late diagnosed patients. Another limitation of this SLR was the inclusion of a substantial number of conference abstracts as part of the grey literature search. While these abstracts were carefully selected to identify the latest evidence not yet available in peer-reviewed journals, and were only included if they met predefined PICOS criteria, they have not undergone the rigorous peer-review process of studies published in peer-reviewed journals, which may introduce potential bias or incomplete information into our findings.

## 5 Conclusion

The findings of this study suggest that patients diagnosed at an early stage with bladder cancer, gastric cancer, HNC, melanoma, NSCLC, RCC, and TNBC generally experienced improved OS, whilst patients diagnosed at a later stage had lower survival rates. Early diagnosis of cancer was also associated with lower healthcare resource utilization and costs compared to late-stage diagnosis, particularly for NSCLC, TNBC, and HNC. Notably, advanced stages of cancer were linked to higher inpatient and end-of-life treatment costs. Although evidence related to HRQoL was scarce and limited to bladder cancer, melanoma, and NSCLC, the findings indicated that patients diagnosed at a more advanced stage have worse HRQoL compared to those diagnosed at earlier, less invasive stages. In conclusion, early detection of cancer plays a vital role in improving clinical and humanistic outcomes and reducing the economic burden associated with a cancer diagnosis. Additional studies investigating the humanistic and economic benefits of early diagnosis across multiple tumor types are encouraged to complement the current body of evidence.

## Data Availability

The original contributions presented in the study are included in the article/**Supplementary Material**. Further inquiries can be directed to the corresponding author.
