# Supplementary material for: Assessing the clinical, humanistic, and economic impact of early cancer diagnosis: a systematic literature review
Source: Front Oncol. 2025 Mar 19;15:1546447. doi: 10.3389/fonc.2025.1546447 (PMC11962897; doi:10.3389/fonc.2025.1546447)
Supplement: Supplementary file 1 [file DataSheet1.pdf]

Supplementary Material

1 Search strategy

Table 1. Search strategy for Embase (conducted on 20th May 2022)

No.	Query	Results
**1**	'melanoma'/exp OR 'melanoma' OR 'melano*'	350589
**2**	'triple negative breast cancer'/exp OR (('breast cancer' OR 'breast tumor' OR 'breast tumour' OR 'breast neoplasm' OR 'breast carcinoma') NEAR/3 ('triple negative' OR 'triple-negative'))	33527
**3**	'non small cell lung cancer'/exp OR 'nsclc' OR 'non-small cell lung cancer' OR 'non-small-cell lung cancer' OR 'non-small':ab,ti OR 'non small':ab,ti OR 'nonsmall':ab,ti OR 'non-small cell':ab,ti OR 'non small cell':ab,ti OR 'nonsmallcell':ab,ti OR 'non-small-cell':ab,ti	211833
**4**	'renal cell carcinoma'/exp OR (renal*:ab,ti AND (carcinoma*:ab,ti OR cancer*:ab,ti OR neoplasm*:ab,ti OR adeno*:ab,ti OR pyelocarcinoma*:ab,ti OR oncocytoma:ab,ti)) OR 'rcc':ab,ti	155309
**5**	'stomach cancer'/exp OR 'gastric cancer'	164199
**6**	'head and neck cancer'/exp OR 'hnscc':ab,ti OR 'hn scc':ab,ti OR 'scchn':ab,ti OR 'scc hn':ab,ti OR 'hnsc':ab,ti OR 'hnc':ab,ti	224160
**7**	'bladder cancer'/exp OR (((bladder OR urothelial OR 'transitional cell') NEAR/3 (cancer OR cancers OR tumor OR tumors OR tumour OR tumours OR carcinoma OR carcinomas OR neoplasm OR neoplasms)):ab,ti) OR 'transitional cell carcinoma'/exp OR 'bladder tumor'/exp OR 'urinary bladder neoplasms'	135990
**8**	#1 OR #2 OR #3 OR #4 OR #5 OR #6 OR #7	1170442
**9**	'early diagnosis'/exp OR (('early' OR 'earlier' OR 'earliest' OR 'earl*') NEAR/3 ('detect*' OR 'diagnos*' OR 'identif*' OR 'presentation')) OR 'early detection' OR 'early identification' OR 'early presentation' OR 'early diagnos*' OR 'early detection cancer' OR 'late diagnos*' OR 'late presentation' OR 'late detection' OR ('time' NEAR/4 ('diagnos*' OR 'detect*')) OR ('delay' NEAR/4 ('diagnos*' OR 'detect*')) OR ('interval' NEAR/4 ('diagnos*' OR 'detect*'))	673969
**10**	'effectiveness':ab,ti OR 'mortality':ab,ti OR 'survival':ab,ti OR 'financ*':ab,ti OR 'econom*':ab,ti OR 'quality of life':ab,ti OR 'economics':ab,ti OR 'economic aspect':ab,ti OR 'cost':ab,ti OR 'health care cost':ab,ti OR 'drug cost':ab,ti OR 'hospital cost':ab,ti OR 'socioeconomics':ab,ti OR 'health economics':ab,ti OR 'pharmacoeconomics':ab,ti OR 'fee':ab,ti OR 'budget':ab,ti OR 'hospital finance':ab,ti OR 'financial management':ab,ti OR 'health care financing':ab,ti OR 'low cost':ab,ti OR 'high cost':ab,ti OR ((health*care NEXT/1 cost*):ab,ti) OR (('health care' NEXT/1 cost*):ab,ti) OR fiscal:ab,ti OR funding:ab,ti OR financial:ab,ti OR finance:ab,ti OR ((cost NEXT/1 estimate*):ab,ti) OR 'cost	9013496

No.	Query	Results
	variable':ab,ti OR ((unit NEXT/1 cost*):ab,ti) OR economic*:ab,ti OR pharmacoeconomic*:ab,ti OR price*:ab,ti OR pricing:ab,ti OR ((health*care NEXT/1 (utilisation OR utilization)):ab,ti) OR (('health care' NEXT/1 (utilisation OR utilization)):ab,ti) OR ((resource NEXT/1 (utilisation OR utilization OR use)):ab,ti) OR ((cost* NEAR/3 (treat* OR therap*)):ab,ti) OR (((direct OR indirect) NEAR/2 cost*):ab,ti) OR 'health related quality of life':ab,ti OR 'health-related quality of life':ab,ti OR 'hrqol':ab,ti OR 'hrql':ab,ti OR 'qol':ab,ti OR 'life quality':ab,ti OR 'health-related qol':ab,ti OR 'quality adjusted life':ab,ti OR 'quality-adjust-life':ab,ti OR 'qaly':ab,ti OR 'qald':ab,ti OR 'qale':ab,ti OR 'qtime':ab,ti OR 'quality adjusted life year':ab,ti OR 'life year*':ab,ti OR 'hql':ab,ti OR 'hqol':ab,ti OR 'h qol':ab,ti OR 'hr qol':ab,ti OR (('quality' NEAR/3 'life'):ab,ti) OR 'qols':ab,ti OR 'quality of life scale':ab,ti OR ((('instrument' OR 'instruments') NEAR/3 ('quality of life' OR 'qol')):ab,ti) OR 'quality of wellbeing':ab,ti OR 'response rate':ab,ti OR 'expense':ab,ti OR 'incidence':ab,ti OR 'prevalence':ab,ti OR 'burden':ab,ti OR 'efficacy':ab,ti OR 'death':ab,ti OR 'cure':ab,ti OR 'impact':ti OR 'reduce':ti OR 'benefit':ti OR 'positive':ti OR 'reduction':ti OR 'decrease':ti OR 'increase':ti OR 'improvement':ti OR 'delay':ti OR 'delayed':ti OR 'favourable':ti OR 'favorable':ti OR 'advantage':ti OR 'convenience':ti OR 'improves':ti	
**11**	'cohort study':ab,ti OR 'retrospective study':ab,ti OR 'cohort analysis':ab,ti OR 'longitudinal study':ab,ti OR 'prospective study':ab,ti OR 'observational study':ab,ti OR ((cohort NEXT/1 stud*):ab,ti) OR ((cohort NEXT/1 analy*):ab,ti) OR 'register':ab,ti OR 'registry':ab,ti OR (('database' NEAR/2 'study'):ab,ti) OR (('real' NEXT/1 'world'):ab,ti) OR (('healthcare' NEXT/1 'record'):ab,ti) OR 'pragmatic trial':ab,ti OR 'real-world clinical trial':ab,ti OR 'pragmatic clinical trial':ab,ti OR 'real-world':ab,ti OR 'real world':ab,ti OR 'database':ab,ti OR 'real-life':ab,ti OR 'real life':ab,ti OR 'database study':ab,ti	2058312
**12**	#8 AND #9 AND #10 AND #11	5802
**13**	#12 AND ([conference abstract]/lim OR [conference paper]/lim OR [conference review]/lim OR [editorial]/lim OR [letter]/lim OR [note]/lim)	2641
**14**	#12 AND [animals]/lim NOT ([humans]/lim AND [animals]/lim)	20
**15**	#13 OR #14	2647
**16**	#12 NOT #15	3155
**17**	#12 NOT #15 AND [english]/lim	2956

Table 2. Search strategy for Medline-In Process (conducted on 20th May 2022)

No.	Query	Results
**1**	"melanoma" [MeSH Terms] OR "melano*"	253659
**2**	"triple negative breast neoplasms" [MeSH Terms] OR (("breast cancer" OR "breast tumor" OR "breast tumour" OR "breast neoplasm" OR "breast carcinoma") AND ("triple negative" OR "triple-negative"))	19113

No.	Query	Results
**3**	"Carcinoma, Non-Small-Cell Lung" [MeSH Terms] OR "nsclc" OR "non-small cell lung cancer" OR "non-small-cell lung cancer" OR "non-small" [Title/Abstract] OR "non small" [Title/Abstract] OR "nonsmall" [Title/Abstract] OR "non-small cell" [Title/Abstract] OR "non small cell" [Title/Abstract] OR "nonsmallcell" [Title/Abstract] OR "non-small-cell" [Title/Abstract]	95871
**4**	"Carcinoma, Renal Cell" [MeSH Terms] OR (renal* [Title/Abstract] AND (carcinoma* [Title/Abstract] OR cancer* [Title/Abstract] OR neoplasm* [Title/Abstract] OR adeno* [Title/Abstract] OR pyelocarcinoma* [Title/Abstract] OR oncocytoma [Title/Abstract])) OR "rcc" [Title/Abstract]	100620
**5**	"Stomach Neoplasms" [MeSH Terms] OR "gastric cancer"	127784
**6**	"Head and Neck Neoplasms" [MeSH Terms] OR "hnscc" [Title/Abstract] OR "hn scc" [Title/Abstract] OR "scchn" [Title/Abstract] OR "scc hn" [Title/Abstract] OR "hnsc" [Title/Abstract] OR "hnc" [Title/Abstract]	343878
**7**	"urinary bladder neoplasms" [MeSH Terms] OR "urinary bladder neoplasms" OR ((bladder [Title/Abstract] OR urothelial [Title/Abstract] OR "transitional cell" [Title/Abstract]) AND (cancer [Title/Abstract] OR cancers [Title/Abstract] OR tumor [Title/Abstract] OR tumors [Title/Abstract] OR tumour [Title/Abstract] OR tumours [Title/Abstract] OR carcinoma [Title/Abstract] OR carcinomas [Title/Abstract] OR neoplasm [Title/Abstract] OR neoplasms [Title/Abstract])) OR "carcinoma, transitional cell" [MeSH Terms] OR "bladder tumor"	102413
**8**	#1 OR #2 OR #3 OR #4 OR #5 OR #6 OR #7	998456
**9**	"early diagnosis"[MeSH Terms] OR (("early" OR "earlier" OR "earliest" OR "earl*") AND ("detect*" OR "diagnos*" OR "identif*" OR "presentation")) OR "early detection" OR "early identification" OR "early presentation" OR "early diagnos*" OR "early detection cancer" OR "late diagnos*" OR "late presentation" OR "late detection" OR ("time" AND ("diagnos*" OR "detect*")) OR ("delay" AND ("diagnos*" OR "detect*")) OR ("interval" AND ("diagnos*" OR "detect*"))	2331809
**10**	"effectiveness"[Title/Abstract] OR "mortality"[Title/Abstract] OR "survival" [Title/Abstract] OR "financ*"[Title/Abstract] OR "econom*"[Title/Abstract] OR "quality of life" [Title/Abstract] OR "economics" [Title/Abstract] OR "economic aspect" [Title/Abstract] OR "cost" [Title/Abstract] OR "health care cost" [Title/Abstract] OR "drug cost" [Title/Abstract] OR "hospital cost" [Title/Abstract] OR "socioeconomics" [Title/Abstract] OR "health economics" [Title/Abstract] OR "pharmacoeconomics" [Title/Abstract] OR "fee" [Title/Abstract] OR "budget" [Title/Abstract] OR "hospital finance" [Title/Abstract] OR "financial management" [Title/Abstract] OR "health care financing" [Title/Abstract] OR "low cost" [Title/Abstract] OR "high cost" [Title/Abstract] OR ("health care"[Title/Abstract] AND cost*[Title/Abstract]) OR ("healthcare"[Title/Abstract] AND cost*[Title/Abstract]) OR ("health-care"[Title/Abstract] AND cost*[Title/Abstract]) OR ("health care"[Title/Abstract] AND cost*[Title/Abstract]) OR fiscal[Title/Abstract] OR funding[Title/Abstract] OR financial[Title/Abstract] OR finance[Title/Abstract] OR (cost[Title/Abstract] AND estimate*[Title/Abstract]) OR "cost variable" [Title/Abstract] OR (unit[Title/Abstract] AND cost*[Title/Abstract]) OR economic*[Title/Abstract] OR pharmacoeconomic*[Title/Abstract] OR price*[Title/Abstract] OR pricing[Title/Abstract] OR (healthcare[Title/Abstract] AND (utilization[Title/Abstract] OR utilization[Title/Abstract])) OR ("health care" [Title/Abstract] AND	6661909

No.	Query	Results
	(utilisation[Title/Abstract] OR utilization[Title/Abstract])) OR (resource[Title/Abstract] AND (utilisation[Title/Abstract] OR utilization[Title/Abstract] OR use[Title/Abstract])) OR (cost*[Title/Abstract] AND (treat*[Title/Abstract] OR therap*[Title/Abstract])) OR ((direct[Title/Abstract] OR indirect[Title/Abstract]) AND cost*[Title/Abstract]) OR "health related quality of life" [Title/Abstract] OR "health-related quality of life" [Title/Abstract] OR "hrqol" [Title/Abstract] OR "hrql"[Title/Abstract] OR "qol" [Title/Abstract] OR "life quality"[Title/Abstract] OR "health-related qol"[Title/Abstract] OR "quality adjusted life"[Title/Abstract] OR "quality-adjust-life"[Title/Abstract] OR "qaly"[Title/Abstract] OR "qald"[Title/Abstract] OR "qale"[Title/Abstract] OR "qtime"[Title/Abstract] OR "quality adjusted life year"[Title/Abstract] OR "life year*"[Title/Abstract] OR "hql"[Title/Abstract] OR "hqol"[Title/Abstract] OR "h qol"[Title/Abstract] OR "hr qol"[Title/Abstract] OR ("quality"[Title/Abstract] AND "life"[Title/Abstract]) OR "qols"[Title/Abstract] OR "quality of life scale"[Title/Abstract] OR (("instrument"[Title/Abstract] OR "instruments"[Title/Abstract]) AND ("quality of life"[Title/Abstract] OR "qol"[Title/Abstract])) OR "quality of wellbeing"[Title/Abstract] OR "response rate"[Title/Abstract] OR "expense"[Title/Abstract] OR "incidence"[Title/Abstract] OR "prevalence"[Title/Abstract] OR "burden"[Title/Abstract] OR "efficacy"[Title/Abstract] OR "death"[Title/Abstract] OR "cure"[Title/Abstract] OR "impact"[Title] OR "reduce"[Title] OR "benefit"[Title] OR "positive"[Title] OR "reduction"[Title] OR "decrease"[Title] OR "increase"[Title] OR "improvement"[Title] OR "delay"[Title] OR "delayed"[Title] OR "favourable"[Title] OR "favorable"[Title] OR "advantage"[Title] OR "convenience"[Title] OR "improves"[Title]	
**11**	"cohort study"[Title/Abstract] OR "retrospective study"[Title/Abstract] OR "cohort analysis"[Title/Abstract] OR "longitudinal study"[Title/Abstract] OR "prospective study"[Title/Abstract] OR "observational study"[Title/Abstract] OR (cohort[Title/Abstract] AND stud*[Title/Abstract]) OR (cohort[Title/Abstract] AND analy*[Title/Abstract]) OR "register"[Title/Abstract] OR "registry"[Title/Abstract] OR ("database"[Title/Abstract] AND "study"[Title/Abstract]) OR ("real" [Title/Abstract] AND "world" [Title/Abstract]) OR ("healthcare"[Title/Abstract] AND "record"[Title/Abstract]) OR "pragmatic trial" [Title/Abstract] OR "real-world clinical trial"[Title/Abstract] OR "pragmatic clinical trial" [Title/Abstract] OR "real-world"[Title/Abstract] OR "real world" [Title/Abstract] OR "database"[Title/Abstract] OR "real-life"[Title/Abstract] OR "real life"[Title/Abstract] OR "database study"[Title/Abstract]	1669677
**12**	#8 AND #9 AND #10 AND #11	14436
**13**	#12 AND ‘conference review’ [Publication Type] OR editorial [Publication Type] OR letter [Publication Type] OR review [Publication Type]	4794734
**14**	#12 NOT #13	13848
**15**	#14 AND (inprocess[sb] OR pubstatusaheadofprint)	203

2 PICOTS eligibility criteria for study inclusion

PICOTS	Inclusion Criteria
**Population(s)**	• Adult (≥18 years) patients of any gender or race, with one of the following cancers at the time of diagnosis:<br>○ Melanoma<br>○ TNBC<br>○ NSCLC (Squamous and Non-squamous)<br>○ RCC<br>○ Gastric cancer<br>○ HNC<br>○ Bladder cancer (Muscle invasive and non-muscle invasive)<br>• Patients were included, regardless of presenting with early/ late-stage, low/ high-risk (i.e., eligible [or not] for chemotherapy or presenting with comorbidities) disease, or with/ without surgical resection
**Interventions**	No restriction
**Comparisons**	No restriction
**Outcomes**	**Survival outcomes by stage at diagnosis**<br>▪ Overall survival<br>▪ Mortality, premature/ avoidable cancer-related deaths<br>• **Humanistic burden by stage at diagnosis**<br>▪ General patient-reported outcomes (e.g., 0-100 scales/questionnaires)<br>▪ Physical, functional, and mobile well-being (e.g., 0-100 scales/questionnaires and qualitative reports)<br>▪ Mental, emotional, and social well-being (either qualitative or quantitative),<br>▪ Fear of cancer progression and/or death, psychological quality of life, qualitative lived experience associated with cancer (either qualitative or quantitative)<br>• **Financial impact associated with stage at diagnosis**

	▪ Direct medical costs for early vs late-stage cancer, including visit fees, tests, treatments, consultations or follow-ups for outpatients, and hospitalization costs, serious illnesses or medical issues that require substantial monitoring, management of complications, treatment administration, length of stay for inpatients<br>▪ Direct non-medical costs, including travel, accommodation, and meals<br>▪ Indirect costs for patients and caregivers (e.g., productivity loss costs including lost wages, absenteeism, early retirement, etc.)
**Time**	No timeframe restriction
**Study design**	Study designs included were:<br>• **Observational studies, including:**<br>▪ Cohort studies (prospective and retrospective)<br>▪ Case control studies (prospective and retrospective)<br>▪ Cross sectional studies<br>▪ Longitudinal studies<br>Reviews (systematic or general reviews) were retrieved for the identification of additional relevant primary studies that may have been missed through the electronic searches.
**Country**	Global
**Other (Language)**	Studies with full texts published in English language only will be included

Abbreviations: HNC: Head and neck cancer; NSCLC: Non-small cell lung cancer; PICOTS: Population, intervention, comparisons, outcomes, time, study design; RCC: Renal cell carcinoma; TNBC: Triple negative breast cancer

3 **Classification and definition of early and late stages of cancer used among the studies included in the SLR that reported their definition of early stages vs. late stages**

Study	Tumor type	Stage classification system	Definition of stage
**Bladder cancer**			
Fisher 2018 (1)	Bladder cancer	NR	Stage IV bladder cancer diagnosis defined as T4b, node-positive, or distant metastatic disease.
**HNC**			
Ho 2019 (2)	Oral cavity cancer	NR	Early stage defined as "Stage 0-I".
Singh 2021 (3)	Oral cancer	AJCC	Stage was defined by the AJCC classification. Patients were stratified according to their stage of disease at diagnosis into early (Stage I, II) and advanced (Stage III, IV) stages as per the latest.
**Melanoma**			
Song 2015 (4)	Metastatic melanoma	AJCC 6th edition	Patients diagnosed with unresectable regionally metastatic refer to stage IIIB/C and distantly metastatic refers to stage IV).
Toscano 2020 (5)	Melanoma	TNM	Patients diagnosed early or non-metastatic stage defined with a stage I or II.
Wilson 2019 (6)	Metastatic melanoma	AJCC 7th edition	Metastatic disease refers to the time at which the patient was diagnosed with metastatic melanoma, stage IV disease.
**NSCLC**			
Berglund 2012 (7)	NSCLC	NR	Early stage defined as "Stage IA-IIB" Late stage defined as "IIIA-IV".
Ehrenstein 2022	NSCLC	NR	Early stage refers to the stage "I-IIIA"

Study	Tumor type	Stage classification system	Definition of stage
(8)			
Flores 2021 (9)	NSCLC	NR	SEER historic staging was used to impute stage for these patients. Those with historic stage values of localized were coded as stage I/II, regional was coded as stage III, and distant was coded as stage IV.
Monteiro 2022 (10)	NSCLC	AJCC 7th edition	Early-stage NSCLC defined as clinical stage I-IIIA.
Snee 2021 (11)	NSCLC	UICC 6th edition (up to 31 December 2009), UICC 7th edition from 1 January 2010 and the 8th edition from 1 January 2017	Early stage refers to stage "I-IIIA", Advanced stage refers to stage "IIIB-IV"
**TNBC**			
Schwartz 2018 (12)	TNBC	AJCC	Patients with stage III and IV defined as advanced stage

Abbreviations: AJCC: American Joint Committee; HNC: Head and neck cancer; NR: Not reported; NR: Not reported; NSCLC: Non-small cell lung cancer; SEER: Surveillance, Epidemiology, and End Results Program; TNBC: Triple negative breast cancer; TNM: Tumor (T), nodes (N), and metastases (M);

4 Summary of studies included in the clinical review (n=52)

Study name	Tumor types	Objective	Study design	Publication type	Country	Data source	Study setting	Time frame	Duration of follow up
**Bladder cancer**									
Amiri 2020 (13)	Bladder cancer	Estimate the survival rate of patients with bladder cancer according to the Cox proportional hazards model based on some key relevant variables.	Retrospective population-based cohort study	Article	Iran	Cancer registry at Provincial Health Department, Cancer Registration Center and Provincial Mortality Registration Center	Provincial population based	2013 to 2018	NR
Aragon-Ching 2021 (14)	Bladder cancer	Evaluate trends between UC vs. nUC histology for upper tract cancers and compare the demographics, disease characteristics, treatment, incidence of stage and survival according to NCDB.	Retrospective observational study	Conference abstract	USA	NCDB	Nationwide population based	2004 – 2017	NR
Davies 2020 (15)	Bladder cancer	Establish a standing cohort of patients with metastatic bladder cancer.	Retrospective observational analysis	Conference abstract	England	NCRAS	Nationwide population based	January 2016-June 2017	NR

Study name	Tumor types	Objective	Study design	Publication type	Country	Data source	Study setting	Time frame	Duration of follow up
Fisher 2018 (1)	Bladder cancer	Understand how patients with stage IV bladder cancer are treated and to add to the limited data that examine effectiveness outcomes associated with that treatment.	Retrospective observational study	Article	USA	Vector Oncology Data Warehouse	Multi-center	1 January 2008- 1 June 2015	NR
Omland 2021 (16)	Metastatic urinary tract cancer	Describe treatment patterns and survival outcomes in mUTC patients treated in the real-world clinical setting.	Nationwide, retrospective, population-based study	Article	Denmark	EHRs from Danish oncology departments	Multi-center	December 2017 and mid 2018 for one Center each; mid 2019 for remaining four Centers.	Median follow-up: 11.6 months
Sorup 2021 (17)	Urothelial Cancer	Characterize treatment patterns, survival outcomes, and HRU in patients with stage IV UC in Denmark.	Population-based, retrospective cohort study	Conference abstract	Denmark	Danish Cancer Registry	Nationwide population based	January 1, 2013-December 31, 2017	Median follow-up: 10.2 months (IQR, 3.6-19.4)
**Gastric cancer**									
Dijksterhuis 2022 (18)	Gastric cancer	Explore use of palliative treatment and overall survival of gastric or GEJ cancer patients and compare overall survival of the patients with interval metastases with gastric or GEJ cancer patients who had distant metastases at initial diagnosis.	Retrospective cohort study	Article in Press	Netherlands	Netherlands Cancer Registry (NCR)	National wide population based	2010 to 2018	NR

Study name	Tumor types	Objective	Study design	Publication type	Country	Data source	Study setting	Time frame	Duration of follow up
Qiu 2018 (19)	Gastric cancer	Analyze metastatic pattern in gastric cancer to help physicians to design imagine examination, especially in making determinations regarding interventions.	Retrospective analysis	Article	USA	SEER	National wide population based	2010 to 2014	NR
**HNC**									
Amarillo 2021 (20)	Head and neck cancer	Identify an impact of delay in the first treatment in OS in head and neck cancer patients.	Retrospective observational cohort study	Conference abstract	Uruguay	Institutional database of the hospital	Single - Center	2005-2015	Median: 6.9 years
Ho 2019 (2)	Oral cavity cancer	Evaluate the effectiveness of the TOMS program in stage-shift among oral cavity cancer patients.	Retrospective cohort study	Article	Taiwan	TCR, TOMS and TDR	National wide population based	2008 to 2015	(From the TDR) or to December 31, 2016 (end of follow-up time).
Hochfelder 2020 (21)	Hypopharyngeal cancer	Examine the association between primary treatment and overall survival among patients with locoregionally advanced hypopharyngeal cancer.	Retrospective observational study	Conference abstract	USA	NCDB	Nationwide population based	2005-2015	NR

Study name	Tumor types	Objective	Study design	Publication type	Country	Data source	Study setting	Time frame	Duration of follow up
Neto 2021 (22)	Oropharynx cancer	To assess the prognostic performance of eighth and seventh AJCC staging edition in patients with p16 positive oropharynx cancer.	Institutional database of the hospital	Conference abstract	USA	Institutional database of the hospital	Nationwide population based	March 2015-December 2018	Median: 34.7 months (2.3-169.74)
Sargeran 2008 (23)	Oral cancer	Analyze 1- to 5-year survival rates for patients with lip cancer in relation to age, sex, stage of the tumor, histological type, and treatment.	Retrospective cohort study	Article	Iran	Iran Ministry of Health	Nationwide population based	1996 to 2002	The patients were followed from the date of diagnosis to late 2005.
Sargeran 2009 (24)	Lip cancer	Analyze 1- to 5-year survival rates for patients with lip cancer in relation to age, sex, stage of the tumor, histological type, and treatment modality.	Retrospective cohort study	Article	Iran	Iran Ministry of Health	Nationwide population based	1996 to 2003	Median: 57 (range 0-112 months); Mean (SD): 56.4 (28) months
**Melanoma**									
Ngo 2020 (25)	Melanoma	Evaluate survival and recurrence patterns of stage II and III cutaneous melanoma.	Retrospective cohort analysis	Conference abstract	NR	NR	NR	2000-2012	NR
Ramond 2021 (26)	Melanoma	Assess availability of data from PHE to study malignant melanoma survival in England.	Retrospective observational study	Conference abstract	England	Public Health England (PHE)	Nationwide population based	1995-2016	NR

Study name	Tumor types	Objective	Study design	Publication type	Country	Data source	Study setting	Time frame	Duration of follow up
Song 2015 (4)	Metastatic melanoma	Describe survival rates among patients diagnosed with unresectable stage IIIB/C or stage IV melanoma.	Retrospective observational study	Article	USA	SEER database	Nationwide population based	2004 to 2009	Mean (SD): 11.3 (138) months
Tjokrowidjaja 2021 (27)	Melanoma	Assess overall survival and cancer-specific survival for AJCC-7 and AJCC-8.	Retrospective study	Article	USA	SEER database	Nationwide population based	2010-2015	NR
Wilson 2019 (6)	Melanoma	Examine the impact of initial stage of melanoma diagnosis, BRAF status of primary melanoma, and receiving adjuvant therapy on post metastatic survival.	Prospective observational study	Article	USA	IMCG database	Single center	August 2002 to December 2015	Post metastasis, median: 25 (range: 0.5 to 167.8) months
Winge-Main 2020 (28)	Melanoma	Investigate the characteristics and outcomes of Norwegian cutaneous melanoma patients with stage IIB-IV.	Retrospective cohort study	Conference abstract	Norway	Cancer Registry of Norway	Nationwide population based	January 2008-December 2018	NR
**NSCLC**									
Abrão 2021 (29)	NSCLC, adenocarcinoma	Examine long-term survival and possible predictors in all patients with stage I and II lung cancer adenocarcinoma.	Hospital-based retrospective cohort study	Article	Brazil	Institutional/city database	Single center	January 2000 to December 31, 2019	Mean: 45.2 (SD 41.3 months); Median:32.8 (Range 1-223 months

Study name	Tumor types	Objective	Study design	Publication type	Country	Data source	Study setting	Time frame	Duration of follow up
Abrão 2022 (30)	NSCLC	Evaluate overall survival and prognostic factors in Stage I NSCLC patients treated only with radiotherapy and surgery in a 19-year follow-up cohort.	Retrospective hospital-based cohort study	Article in Press	Brazil	Sao Paulo's Oncocentro, FOSP	Multi-center	2000 and 2015	Mean: 51.2 (SD: 41.4) months
Azzouqa 2019 (31)	NSCLC	Determine if there is an association between time to treatment initiation and survival in patients with NSCLC.	Retrospective cohort study	Conference abstract	USA	Mayo Clinic Cancer Center registry	Multi-center	2000 to 2016	NR
Berglund 2012 (7)	NSCLC	Examine possible social variations in lung cancer survival and assess if any such gradients can be attributed to social differences in comorbidity, stage at diagnosis or treatment.	Retrospective observational study	Article	UK	Thames Cancer Registry	Nationwide population based	2006-2008	NR
Cerqueira 2022 *Linked to:* Cerquiera 2022 (32)	NSCLC	Evaluate NSCLC stage III/IV patients' journey in the Brazilian supplementary healthcare system.	Population based retrospective cohort study	Conference abstract	Brazil	Hospital-based cancer registry database	Private hospital based	2016-2018	NR
Ehrenstein 2022 (8)	NSCLC	Describe EGFR testing, patient characteristics, and overall survival among patients with early-stage NSCLC.	Retrospective observational study	Article	Denmark	Danish Lung Cancer Registry	Nationwide population based	2013 to 2018	Until 10 September 2019

Study name	Tumor types	Objective	Study design	Publication type	Country	Data source	Study setting	Time frame	Duration of follow up
Ekman 2019 (33)	NSCLC	Report initial treatment and OS for patients with NSCLC prior to the availability of immunotherapies in Sweden.	Retrospective observational study	Conference abstract	Sweden	Institutional databases of University Hospitals	Multi-center	2012 to 2015 (follow-up to December 2016)	Followed up to Dec 2016
Flores 2021 (9)	NSCLC	Investigate association of stage shift with population mortality among patients with NSCLC.	Retrospective cohort study	Article	USA	SEER	National wide population based	2006 to 2016	Median: 61 Months [IQR 21-95]
Greystoke 2021 (34)	NSCLC	Assess patient characteristics, treatment patterns, and OS in patients with NSCLC.	Retrospective observational study	Conference abstract	England	Public Health England's CAS	Nationwide population based	2013-2018	NR
Jazieh 2021 *Linked to:* Jazieh 2020 (35)	NSCLC	Determine the treatment patterns and their associated clinical outcomes in patients with stage III NSCLC, as defined by the AJCC criteria (seventh edition) in the pre-PACIFIC study era.	Retrospective noninterventio nal study	Article	19 Countries	Medical records from hospitals (from Asia, Middle East, Africa, and Latin America)	Multi-center, Multinational	January 2013-December 2017	At least 9 months of documented follow-up since index diagnosis
Kalilani 2022 (36)	NSCLC	Provide recent estimates of survival in patients with advanced NSCLC in the USA.	Retrospective observational study	Conference abstract	USA	SEER database and Flatiron Health database	Nationwide population based	1). SEER database: 2010–2016 2). Flatiron Health database: 2017–2020	NR

Study name	Tumor types	Objective	Study design	Publication type	Country	Data source	Study setting	Time frame	Duration of follow up
Klarenbeek 2022 (37)	NSCLC	Examine factors associated between time-to-treatment and overall survival in a large nationwide retrospective cohort.	Large nationwide retrospective study	Article in Press	Netherlan ds	The Netherlands Cancer Registry	National wide population based	2014 to 2019	NR
Komiya 2020 (38)	NSCLC	Study the role of T0 status in OS for unresectable stage III non-small cell lung cancer.	Retrospective study	Conference abstract	USA	National Cancer Database	Nationwide population based	2004-2016	NR
Luciano 2020 (39)	NSCLC	Assess overall survival, TTT, follow-up time, and treatment pathways in metastatic NSCLC patients treated with programmed cell death protein 1 inhibitors.	Retrospective observational analysis	Conference abstract	USA	A USA based EMR network	Nationwide population based	NR	Advanced diagnosis: 21.87 months [IQR 11.94-38.97] From inhibitor initiation: 8.71 months (3.06, 17.26)
Martin 2022 (40)	NSCLC	Report on treatment patterns and their associated clinical outcomes in patients with stage III NSCLC in the LATAM subset from the pre-immuno- oncology era.	Retrospective study	Article	Argentina, Chile, Colombia, Dominica n Republic, Mexico, Peru and Uruguay	LATAM subset from KINDLE Study	Multi-center	January 2013-December 2017	NR

Study name	Tumor types	Objective	Study design	Publication type	Country	Data source	Study setting	Time frame	Duration of follow up
Monteiro 2022 (10)	NSCLC	Evaluate impact of microvascular invasion on the 5-year OS of patients with resected NSCLC treated at the INCA.	Retrospective, observational cohort study	Article	Brazil	Institutional database of the hospital	Single center	January 2010 and December 2016	Median: 83 months
Potter 2022 (41)	NSCLC	Determine the effect of the introduction of low-dose CT screening in 2013 on lung cancer stage shift, survival, and disparities in the stage of lung cancer diagnosed in the USA.	Retrospective analysis, quasi-experimental study	Article	USA	The National Cancer Database	National wide population based	2010 and 2018	NR
Rittberg 2021 (42)	NSCLC	Evaluate real world, population-based outcomes for Stage IV NSCLC to assess impact of changing therapies on referral, treatment patterns and OS, which may help explain ongoing stigma/nihilism.	Retrospective cohort study	Conference abstract	Canada	NR	Real world, population-based study	2006-2015	NR
Snee 2021 (11)	NSCLC	Report characteristics, treatment and overall survival trends, by stage and pathology, of patients diagnosed with NSCLC.	Retrospective cohort study	Article	UK	Leeds Teaching Hospital NHS Trust	Single center	January 2007-August 2017	Date of death or end of study (April 2018)

Study name	Tumor types	Objective	Study design	Publication type	Country	Data source	Study setting	Time frame	Duration of follow up
Soares 2021 (43)	NSCLC	Evaluate treatment patterns and survival for patients with stage I–IIIA NSCLC.	Retrospective observational cohort study	Article	Portugal	IPO-Porto oncology hospital	Single center	January 2012-December 2016	Till June 2017
Suipyte 2019 (44)	NSCLC	Evaluate patient with stage IV NSCLC in a real life setting and its impact on OS over the past 10 years.	Retrospective cohort study	Conference abstract	Switzerlan d	Institutional database of the hospitals	Multi-center	2005-2007 and 2015-2016	NR
Vachani 2021 (45)	NSCLC	Quantify the association of stage at diagnosis with OS and MHE in the first year after diagnosis in patients with NSCLC.	Retrospective observational study	Conference abstract	USA	SEER Database	Nationwide population based	2006-2015	12 months
Van Dao 2022 (46)	NSCLC	Characterize treatment patterns and clinical outcomes of patients with stage III NSCLC.	Retrospective observational study	Article	Vietnam	2 hospitals in Vietnam (one each in North and South Vietnam)	Multi-center	2013-2017	Mean (SD): 17.52 (13.81) months.
**Renal cell carcinoma**									
Haas 2022 (47)	Renal cell carcinoma	Evaluate recurrence rate and overall survival outcomes by disease stage and incremental impact of time to recurrence on overall survival in localized RCC.	Retrospective observational study	Conference abstract	USA	SEER-Medicare database	Nationwide population based	2007-2016	Median: 23 months

Study name	Tumor types	Objective	Study design	Publication type	Country	Data source	Study setting	Time frame	Duration of follow up
Haas 2022 (48)	Renal cell carcinoma	Evaluate disease free survival and overall survival patterns and risk of overall survival among patients with non-metastatic RCC in the USA.	Retrospective study	Conference abstract	USA	ConcertAI Oncology Dataset	Nationwide population based	2012-2015	Median: 49.5 months
Li 2021 (49)	ccRCC	Explore baseline characteristics, pathological and survival outcomes of Asian-American patients with ccRCC and make comparisons with White patients.	Retrospective population-based analysis	Article	USA	SEER database	Nationwide population based	2010-2015	Median: 35 [IQR 19-55] months
**TNBC**									
Aly 2019 (50)	TNBC	Estimate overall survival, treatment patterns and economic burden of elderly metastatic TNBC patients.	Retrospective, observational study	Article	USA	SEER-Medicare database	National wide population based	2004 – 2011	Mean: 14.1 months
Gogate 2022 (51)	TNBC	Describe clinical parameters by receipt of systemic therapy and to assess OS, PFS after NAT and adjuvant therapy in women with early HR+/HER2− or TNBC using RWE in the USA.	Retrospective observational study	Conference abstract	USA	Flatiron Health nationwide electronic health record-derived de-identified database	Nationwide real-world setting	1 January 2011 to 31 May 2018	NR

Study name	Tumor types	Objective	Study design	Publication type	Country	Data source	Study setting	Time frame	Duration of follow up
Lehrberg 2021 (52)	TNBC	Evaluate survival rate and phenotype of patients with early stage breast cancer with negative SLN biopsy.	Retrospective analysis	Article	USA	The Henry Ford Health System	Single-center	October 1998 to February 2017	Mean (range): White American women, 95 (0, 218) months African American women,: 86 (3, 218)
Schwartz 2018 (12)	TNBC	Identify and characterize elderly patients with advanced TNBC with respect to baseline demographics and comorbidities, treatment, including chemotherapy regimens, by specific type of therapy, survival patterns, HRU and costs.	Retrospective analysis	Article	USA	SEER	National wide population based	January 2007 to January 2011	Through to December 31, 2013
Sieluk 2020 (53)	TNBC	Provide insights into patient characteristics, as well as clinical and economic outcomes for elderly patients with early stage TNBC, treated from 2010-2016 in the USA.	Retrospective	Conference abstract	USA	SEER-Medicare database	Multi-center	2010 – 2015	Median (range): 20.2 months (2.4-84.1)

Study name	Tumor types	Objective	Study design	Publication type	Country	Data source	Study setting	Time frame	Duration of follow up
Yousefi 2017 (54)	TNBC	Analyze the clinical, pathological profile and survival, recurrence of TNBC patients and find the factors effective in recurrence in breast cancer patients and compared them with patients without recurrence, and prognostic factors effective in the patients' death.	Retrospective study	Article	Iran	Institutional database	Single center	September 2002 to December 2014	Median: 41 months (range 4.2 – 208)

Abbreviations: 1R: One regimen; 2R: Two regimens; 3R+: Three or more regimens; 5-FU: Fluorouracil; AI: American Indian; AJCC: American Joint Committee on Cancer; AL: Acral lentiginous; AN: Alaska native; ASCC: Adenosquamous cell carcinoma; BC: Bronchioloalveolar carcinoma; CA: Cerebrovascular accident; CaEto: Carboplatin and etoposide; CaG: Carboplatin and gemcitabine; CAS: Cancer analysis system; CCI: Charlson Comorbidity Index; CHF: Congestive heart failure; COPD: Chronic pulmonary obstructive disease; CPD: Chronic pulmonary disease; EC: Epidermoid carcinoma; ECOG PS: Eastern Cooperative Oncology Group performance score; EGFR: Estimated glomerular filtration rate; GC: Gemcitabine and cisplatin; Gem: Gemcitabine; HER2: Human epidermal growth factor receptor 2; HTN: Hypertension; ICD: International Classification of Diseases; IDC: Invasive ductal carcinoma; IDM: Infiltrating ductal mixed; ILC: Invasive lobular carcinoma; ILM: Infiltrating lobular mixed; IQR: Interquartile range; LA: Locally advanced; LCC: Large cell carcinoma; MA: Mucinous adenocarcinoma; MI: Myocardial infarction; MVAC: Methotrexate, vinblastine, doxorubicin and cisplatin; NA: Not available; NOS: Not otherwise specified; NPCR: National Program of Cancer Registries; NR: Not reported; NSCLC: Non-small cell lung cancer; NSQ: Non-squamous; RCC: Renal cell carcinoma; RD: Renal disease; SACT: Systemic anticancer therapy; SCC: Squamous cell carcinoma; SCNCC: Small-cell neuroendocrine carcinoma; SD: Standard deviation; SEER: Surveillance, Epidemiology, and End Results Program; SQ: Squamous; SRCC: Signet ring cell carcinoma; SS: Superficial spreading; TNBC: Triple negative breast cancer; TNM: Tumor (T), nodes (N), and metastases (M); UC: Urothelial cancer; UICC: Union for International Cancer Control

Population characteristics

Study name	Patient population	Stage at diagnosis	Sample size (N)	Age (Year)	Gender, n (%)	Race/ ethnicity	Stage distribution	ECOG PS	Tumor histology	Comorbidities
**Bladder cancer**										
Amiri 2020 (13)	All stages	All stages	321	NR	Male: 264 (82.20) Female: 57 (0.177)	NR	Poorly differentiated: 217 (67.4) Well differentiated: 104 (32.4)	NR	Adenocarcinoma: 10 (3.1) SCC: 7 (2.1) UC: 304 (94.7)	Yes: 116 (34.1) No: 205 (63.8)
Aragon-Ching 2021 (14)	Urothelial carcinoma	All stages	29743	Median: 73	NR	White: Caucasian: 92% African American: 4%	Stage IV: NR (8.1)	NR	NR	NR
	Non-urothelial carcinoma	All stages	561	Median: 72	NR	White: Caucasian: 91% African American: 6%	Stage IV: NR (29)	NR	NR	NR
Davies 2020 (15)	Patients with metastatic urothelial cancer	Metastatic	2,543	Mean: 74 (SD: 11.1)	Male: 64% Female: 36%	NR	NR	NR	Transitional cell morphology: NR (73)	Hypertension: 24 Type-2 diabetes: 9
Fisher 2018 (1)	Patients with stage IV bladder cancer	Stage IV	508	Mean: 69.7(SD: 11.1) Median: 71(range : 35-92)	Male: 382 (75.2) Female: 126 (24.8)	White:402 (79.1) Black: 78 (15.4) Asian:1 (0.2) Others: Latino: 3 (0.6) Other/undocumented: 24 (4.7)	ICD: Stage I: 38 (7.5); II: 63 (12.4); III: 47 (9.3); IV: 158 (31.1); Unknown: 23 (45) Undocumented: 179(35.2)	NR	NR	Diabetes: 119 (23.4) RD: 84 (16.5) COPD: 63 (12.4) MI: 41 (8.1) CHF: 26 (5.1) CA: 24 (4.7

Study name	Patient population	Stage at diagnosis	Sample size (N)	Age (Year)	Gender, n (%)	Race/ ethnicity	Stage distribution	ECOG PS	Tumor histology	Comorbidities
	Patients with stage IV bladder cancer (<65 Years)	Stage IV	145	Mean: 55.7(SD: 6.7) Median: 57(range : 35-64)	Male: 109 (75.2) Female: 36 (24.8)	White:107 (73.8) Black: 27 (18.6) Asian:1 (0.7) Others: Latino: 2 (0.6) Other/undocumented: 8 (5.5)	ICD: Stage I: 10 (6.9); II: 13 (9); III: 16 (11); IV: 56 (38.6); Unknown: 8 (5.5) Undocumented: 42(29)	NR	NR	Diabetes: 27 (18.6) RD: 13 (9.0) COPD: 10 (6.9) MI: 6 (4.1) CHF: 3 (2.1) CA: 1 (0.7)
Omland 2021 (16)	All patients with metastatic Urinary tract Cancer who initiated first-line chemotherapy	Overall	952	Median: 69 [IQR: 63-75]	Male: 686 (72.1) Female: 266 (27.9)	NR	NR	Status 0: 341 (35.8) Status 1: 321 (33.7) Status 2: 135 (14.2) Status 3: 5 (0.5) Status unknown: 150 (15.8)	UC: 879 (92.3%) SCC: 27 (2.8%) Adenocarcinoma: 10 (1.1%) SCNCC: 29 (3.0%) Other: 5 (0.5%) Unknown: 2 (0.2%)	NR
	Patients initiated GC as first-line treatment	Overall	440	Median: 67 [IQR: 61-71]	Male: 339 (77) Female: 101 (23)	NR	NR	Status 0: 205 (46.6) Status 1: 129 (29.3) Status 2: 34 (7.7) Status 3: 1 (02) Status unknown: 71 (16.1)	UC: 414 (94.1%); SCC: 14 (3.2%) Adenocarcinoma: 7 (1.6%) SCNCC: 0 (0.0%) Other: 4 (0.9%) Unknown: 1 (0.2%)	NR

Study name	Patient population	Stage at diagnosis	Sample size (N)	Age (Year)	Gender, n (%)	Race/ ethnicity	Stage distribution	ECOG PS	Tumor histology	Comorbidities
	Patients initiated GC split course as first-line treatment	Overall	84	Median: 68 [IQR: 63-73]	Male: 64 (76.2) Female: 20 (23.8)	NR	NR	Status 0: 35 (41.7) Status 1: 34 (40.5) Status 2: 4 (4.8) Status 3: 0 (0) ECOG unknown: 11 (13.1)	UC: 79 (94.0%) SCC: 4 (4.8%) Adenocarcinoma: 1 (1.2%) SCNCC: 0 Other: 0 Unknown: 0	NR
	Patients initiated CaG as first-line treatment	Overall	201	Median: 72 [IQR: 66-76]	Male: 131 (65.2) Female: 70 (34.8)	NR	NR	Status 0: 33 (21) Status 1: 48 (30.6) Status 2: 47 (29.9) Status 3: 3 (1.9) ECOG unknown: 26 (16.6)	UC: 194 (96.5%) SCC: 5 (2.5%) Adenocarcinoma: 2 (1.0%) SCNCC: 0 Other: 0 Unknown: 0	NR
	Patients initiated Gem as first-line treatment	Overall	157	Median: 76 [IQR: 70-78]	Male: 104 (66.2) Female: 53 (33.8)	NR	NR	Status 0: 33 (21) Status 1: 48 (30.6) Status 2: 47 (29.9) Status 3: 3 (1.9) ECOG unknown: 26 (16.6)	UC: 154 (98.0%) SCC: 2 (1.3%) Adenocarcinoma: 0 SCNCC: 0 Other: 1 (0.6%) Unknown: 0	NR
	Patients initiated CaEto as first-line treatment	Overall	35	Median: 69 [IQR: 63-76]	Male: 25 (71.4) Female: 10 (28.6)	NR	NR	Status 0: 20 (57.1) Status 1: 7 (20) Status 2: 5 (14.3) Status 3: 0 (0) Unknown: 3 (8.6))	UC: 8 (22.9%) SCC: 0 Adenocarcinoma: 0 SCNCC: 26 (74.3%) Other: 0 Unknown: 1 (2.9%)	NR

Study name	Patient population	Stage at diagnosis	Sample size (N)	Age (Year)	Gender, n (%)	Race/ ethnicity	Stage distribution	ECOG PS	Tumor histology	Comorbidities
	Patients initiated other treatments (MVAC, vinflunine/gemcitabine, cisplatin/gemcitabine/lapatinib, paclitaxel/ carboplatin, paclitaxel/ gemcitabine, and docetaxel/cisplatin/5-FU) as first-line treatment	Overall	35	Median: 63 [IQR: 59-71]	Male: 23 (65.7) Female: 12 (34.3)	NR	NR	Status 0: 20 (57.1) Status 1: 7 (20) Status 2: 5 (14.3) Status 3: 0 (0) Unknown: 3 (8.6))	UC: 30 (85.7%) SCC: 2 (5.7%) Adenocarcinoma: 0 SCNCC: 3 (8.6%) Other: 0 Unknown: 0	NR
Sorup 2021 (17)	Patients with incident stage IV urothelial cancer	Stage IV	620	Median: 72.2 [IQR: 65.6-78.2]	Male: 394 (63.5) Female: 226 (36.5)	NR	Stage IV: 620 (100)	NR	NR	NR
**Gastric cancer**										
Dijksterhuis 2022 (18)	Gastric adenocarcinoma diagnosed with distant interval metastases	Overall	164	Median: 66 [IQR: 58-72]	Male: 98 (60) Female: 66 (40)	NR	TNM 7th and 8th: Stage I-II: 50 (30) Stage III: 74 (45) Stage Iva: 15 (9)	Status 0/1: 114 (69) Status 2: 8 (5) Status unknown: 42 (26)	Intestinal: 46 (28) Diffuse: 75 (46) Mixed: 7 (4) Indeterminate: 4 (2) Signet ring cell histology: 32 (20)	No. of comorbidities 0: 93 (57) No. of comorbidities 1: 45 (27) No. of comorbidities >2: 17 (10)

Study name	Patient population	Stage at diagnosis	Sample size (N)	Age (Year)	Gender, n (%)	Race/ ethnicity	Stage distribution	ECOG PS	Tumor histology	Comorbidities
										No. Of comorbidities Unknown: 9 (5)
Qiu 2018 (19)	Overall	Overall	19022	Median: 66	Male: 12208 (64.18) Female: 6814 (35.82)	NR	AJCC 7th: Stage I: NR (21.63); II: NR (14.92); III: NR (22.48); IV: NR (40.96)	NR	NR	NR
	Patients with no liver metastases	Overall	NR	Mean: 65.74 (SD: 14.15)	Male: 9703 (80.84) Female: 5753 (86.24)	White: 10743 (82.21) Asian: 2413 (87.68) African American: 1909 (80.18)	NR	NR	Adenocarcinoma: 11459 (79.74) Mucinous: 327 (86.28) SRCC: 3670 (93.53)	NR
	Patients with liver metastases	Overall	NR	Mean: 65.66 (SD: 13.20)	Male: 2300 (19.16) Female: 918 (13.76)	White: 2325 (17.79) Asian: 339 (12.32) African American: 472 (19.82)	NR	NR	Adenocarcinoma: 2912 (20.26) Mucinous: 52 (13.72) SRCC:254 (6.47)	NR
	Patients with no lung metastases	Overall	NR	Mean: 65.82 (SD: 13.95)	Male: 11148 (93.57) Female: 6276 (94.58)	White: 12128 (93.56) Asian: 2623 (95.76) African American: 2234 (93.91)	NR	NR	Adenocarcinoma: 13322 (93.5) Mucinous: 350 (93.09) SRCC: 3752 (95.57)	NR
	Patients with lung metastases	Overall	NR	Mean: 64.21 (SD: 14.52)	Male: 766 (6.43) Female: 360 (5.42)	White: 835 (6.44) Asian: 116 (4.24) African American: 145 (6.09)	NR	NR	Adenocarcinoma: 926 (6.5) Mucinous: 26 (6.91) SRCC: 174 (4.43)	NR

Study name	Patient population	Stage at diagnosis	Sample size (N)	Age (Year)	Gender, n (%)	Race/ ethnicity	Stage distribution	ECOG PS	Tumor histology	Comorbidities
	Patients with no bone metastases	Overall	NR	Mean: 65.99 (SD: 13.93)	Male: 11302 (94.59) Female: 6322 (95.18)	White: 12300 (94.53) Asian: 2614 (95.47) African American: 2265 (95.45)	NR	NR	Adenocarcinoma: 13624 (95.47) Mucinous: 362 (94.52) SRCC: 3638 (92.41)	NR
	Patients with bone metastases	Overall	NR	Mean: 60.68 (SD: 13.99)	Male: 646 (5.41) Female: 320 (4.82)	White: 712 (5.47) Asian: 124 (4.53) African American: 108 (4.55)	NR	NR	Adenocarcinoma: 646 (4.53) Mucinous: 21 (5.48) SRCC: 299 (7.59)	NR
	Patients with no brain metastases	Overall	NR	Mean: 65.75 (SD: 13.98)	Male: 11816 (99.05) Female: 6590 (99.43)	White:12868 (99.02) Asian:2717 (99.49) African American: 2356 (99.58)	NR	NR	Adenocarcinoma: 14134 (99.18) Mucinous: 376 (99.47) SRCC: 3896 (99.19)	NR
	Patients with brain metastases	Overall	NR	Mean: 61.34 (SD: 13.64)	Male: 113 (0.95) Female: 38 (0.57)	White: 127 (0.98) Asian: 14 (0.51) African American: 10 (0.42)	NR	NR	Adenocarcinoma: 117 (0.82) Mucinous: 2 (0.53) SRCC: 32 (0.81)	NR
**HNC**										
Amarillo 2021 (20)	Patients who received treatment	All stages	377	NR	NR	NR	NR	NR	NR	NR

Study name	Patient population	Stage at diagnosis	Sample size (N)	Age (Year)	Gender, n (%)	Race/ ethnicity	Stage distribution	ECOG PS	Tumor histology	Comorbidities
Ho 2019 (2)	NR	Overall	36,139	NR	NR	NR	ICD-C-O (C00-C06): Stage I: 10383 (30.2); II: 6627 (19.3); III: 3954 (11.5); IV: 13134 (38.2); Unknown: Other: 1766 (NR)	NR	NR	NR
	NR	Stage 0	275	Median: 55	Male: 239 (86.9) Female: 36 (13.1)	NR	NR	NR	NR	NR
	NR	Stage I	10,383	Median: 53	Male: 9158 (88.2) Female: 1225 (11.8)	NR	NR	NR	NR	NR
	NR	Stage II	6,627	Median: 54	Male: 6037 (91.1) Female: 590 (8.9)	NR	NR	NR	NR	NR
	NR	Stage III	3,954	Median: 53	Male: 3630 (91.8) Female: 324 (8.2)	NR	NR	NR	NR	NR
	NR	Stage IV	13,134	Median: 53	Male: 12083 (92) Female: 1051 (8)	NR	NR	NR	NR	NR
	NR	Other (no stage information)	1,766	Median: 53	Male: 1543 (87.4) Female: 223 (12.6)	NR	NR	NR	NR	NR

Study name	Patient population	Stage at diagnosis	Sample size (N)	Age (Year)	Gender, n (%)	Race/ ethnicity	Stage distribution	ECOG PS	Tumor histology	Comorbidities
Hochfelder 2020 (21)	Stage III or IV, M0, hypopharyngeal SCC patients	Stage III or IV	5,272	NR	NR	NR	NR	NR	NR	NR
	Native Hawaiian/ Pacific Islander	Overall	469	Mean: 57.8 (SD: 12.3)	Male: 340 (72.5) Female: 129 (27.5)	Native/ Hawaiian/Pacific Islander: 469 (100)	NR	NR	NR	NR
	Non-Hispanic White	Overall	71,110	Mean: 62.2 (SD: 12.1)	Male: 54274 (76.3) Female:16838 (23.7)	Non-Hispanic White: 71110 (100)	NR	NR	NR	NR
Neto 2021 (22)	AJCC 7th edition	All stages	463	NR	NR	NR	AJCC 7th: Stage I: 17 (NR); II: 29 (NR); III: 54 (NR); IVA: 319 (NR); IVB: 19 (NR); IVC: 25 (NR)	NR	NR	NR
	AJCC 8th edition	All stages	463	NR	NR	NR	AJCC 8th: Stage I: 279 (NR); II: 94 (NR); III: 65 (NR); IV: 25 (NR)	NR	NR	NR
Sargeran 2009 (24)	Overall	Overall	82	Mean: 58.6 (SD: 15.2) Median: 62 (range: 27-85)	Male: 70 (85) Female: 12 (15)	NR	Stage I: 35 (43); II: 17 (21); III: 11 (13); IV: 9 (11); Unknown: 10 (12)	NR	NR	NR

Study name	Patient population	Stage at diagnosis	Sample size (N)	Age (Year)	Gender, n (%)	Race/ ethnicity	Stage distribution	ECOG PS	Tumor histology	Comorbiditi es
Sargeran 2008 (23)	Overall	Overall	82	Mean: 61 (SD: 15)	Male: 257 (55) Female: 213 (45)	NR	ICD 10: Stage of tumor I: 92 (20) II: 73 (16) III: 70 (15) IV: 167 (35)	NR	NR	NR
**Melanoma**										
Ngo 2020 (25)	Stage II and III cutaneous melanoma	Stage II and III	169	Median: 63.2 (range: 14-93)	Male: 106 (62.7) Female: 63 (37.3)	NR	AJCC 8th: Stage II: 81(47.9), Stage III: 88 (52.1)	NR	NR	NR
	Stage II cutaneous melanoma	Stage II	81	Median: 69.3	NR	NR	NR	NR	NR	NR
	Stage III cutaneous melanoma	Stage III	88	Median: 58.1	NR	NR	NR	NR	NR	NR
Ramond 2021 (26)	Overall patients with melanoma	All stages	184,864	NR	NR	NR	NR	NR	NR	NR
Song 2015 (4)	Overall stage at diagnosis	Overall	1682	Mean: 64 (SD: 15.3)	Male: 68% Female: 32%	White: 91.7% Black: 1.7% Asian: 1.4% Hispanic: 4.8% Others: 0.3% (AI/ AN) Unknown: 0.2%	AJCC 6th: Stage IIIB/C: 74 (4.40) Stage IV: Stage M1A: 212 (126) Stage M1B: 292 (17.4) Stage M1C: 1104 (65.6)	NR	NR	NR
	Stage IIIB/C at diagnosis	Stage at diagnosis	74	Mean: 63.1 (SD: 18.9)	Male: 66.2% Female: 33.8%	White: 96% Black: 1.4% Asian: 0% Hispanic: 2.7% Others: 0% (AI/ AN): 0 Unknown: 0%	NR	NR	NR	NR

Study name	Patient population	Stage at diagnosis	Sample size (N)	Age (Year)	Gender, n (%)	Race/ ethnicity	Stage distribution	ECOG PS	Tumor histology	Comorbidities
	Stage IV M1A at diagnosis	Stage at diagnosis	212	Mean: 64.8 (SD: 16.3)	Male: 63.7% Female: 36.3%	White: 93.4% Black: 1.9% Asian: 1.9% Hispanic: 2.4% Others: 0% (AI/ AN) Unknown: 0.5%	NR	NR	NR	NR
	Stage IV M1B at diagnosis	Stage at diagnosis	292	Mean: 67.8 (SD: 13.3)	Male: 67.8% Female: 32.2%	White: 93.5% Black: 2.4% Asian: 0% Hispanic: 4.1% Others: NR	NR	NR	NR	NR
	Stage IV M1C at diagnosis	Stage at diagnosis	1104	Mean: 62.9 (SD: 15.2)	Male: 69% Female: 31%	White: 90.6% Black: 1.5% Asian: 1.7% Hispanic: 5.5% AI/ AN: 0.5% Unknown: 0.3%	NR	NR	NR	NR
Tjokrowidjaja 2021 (27)	Melanoma overall	Overall	59,989	NR	Male: 33353 (55) Female: 26636 (45)	NR	NR	NR	NR	NR

Study name	Patient population	Stage at diagnosis	Sample size (N)	Age (Year)	Gender, n (%)	Race/ ethnicity	Stage distribution	ECOG PS	Tumor histology	Comorbidities
	Melanoma overall	Overall	NR	NR	NR	NR	AJCC 7th pathological stage groups: IA: 26,944 (45); IB: 18,507 (31); IIA: 4117 (7); IIB: 2829 (5) IIC: 1441 (2) Stage III: IIIA: 1160 (2) IIIB: 1463 (2) IIIC: 925 (2); IV: 2603 (4)	NR	NR	NR
	Melanoma overall	Overall	NR	NR	NR	NR	AJCC 8th pathological stage groups: IA: 38,344 (64) IB: 7099 (12) Stage II: IIA: 4116 (7) IIB: 2833 (5) IIC: 1445 (2) Stage III: IIIA: 855 (1) IIIB: 909 (1) IIIC: 1730 (3) IIID: 54 (1); IV: 2604 (4)	NR	NR	NR

Study name	Patient population	Stage at diagnosis	Sample size (N)	Age (Year)	Gender, n (%)	Race/ ethnicity	Stage distribution	ECOG PS	Tumor histology	Comorbidities
	Melanoma overall	Overall	NR	NR	NR	NR	AJCC 7th clinical stage groups: AJCC Stage I: IA: 26,944 (45) IB: 18,429 (30) Stage II: IIA: 4117 (7) IIB: 2829 (5) IIC: 1441(2) Stage III: 4048 (7) Stage IV: 2603 (4)	NR	NR	NR
	Melanoma overall	Overall	NR	NR	NR	NR	AJCC 8th clinical stage groups: AJCC Stage I: IA: 32,143 (53) IB: 13,225 (22) Stage II: IIA: 4118 (7) IIB: 2835 (5) IIC: 1446 (2) Stage III: 4040 (7) Stage IV: 2604 (4)	NR	NR	NR
Wilson 2019 (6)	Overall	Overall	304	Median: 60 (range: 18–93)	Male: 202 (66.4) Female: 102 (33.6)	NR	AJCC 7th stage: IA: 25 (8.2); IB: 71 (23.4); IIA: 30 (9.9); IIB: 41 (13.5); IIC: 28 (9.2); IIIA: 29 (9.5); IIIB: 46 (15.1); IIIC: 34 (11.2).	ECOG status 0: 204 (77.9)	AL: 19 (8.1) Nodular: 123 (52.6) SS: 69 (29.5) Other: 23 (9.8)	NR

Study name	Patient population	Stage at diagnosis	Sample size (N)	Age (Year)	Gender, n (%)	Race/ ethnicity	Stage distribution	ECOG PS	Tumor histology	Comorbidities
	Stage I at diagnosis	Stage I	96	Median: 55 (range: 18–82)	Male: 64 (66.7) Female: 32 (33.3)	NR	AJCC 7th stage: IA: 25 (26) IB: 71 (74)	ECOG status 0: 67 (80.7)	AL: 5 (7.8) Nodular: 16 (25.0); SS: 38 (59.4); Other: 5 (7.8)	NR
	Stage II at diagnosis	Stage II	99	Median: 67 (range: 31–93)	Male: 69 (69.7) Female: 30 (30.3)	NR	AJCC 7th stage: Stage II: Stage IIA: 30 (30.3); IIB: 41 (41.4); IIC: 28 (28.3).	ECOG status 0: 72 (79.1)	AL: 4 (4.5) Nodular: 57 (64.0) SS: 13 (14.6) Other: 15 (16.9)	NR
	Stage III at diagnosis	Stage III	109	Median: 57 (range: 20–84)	Male: 69 (63.3) Female: 40 (36.7)	NR	AJCC 7th stage: IIIA: 29 (26.6); IIIB: 46 (42.2); IIIC: 34 (31.2)	ECOG status 0: 65 (73.9)	AL: 10 (12.3) Nodular: 50 (61.7) SS: 18 (22.2) Other: 3 (3.7)	NR
Winge-Main 2020 (28)	Patients with cutaneous melanoma	Overall	4,339	Median: 72 (range: 60-82)	Male: 57.9% Female: 42.1%	NR	ICD-10, AJCC8: Stage II: NR (IIB: 35.7; IIC: 17.7) (IIIA: 4; IIIB: 8.2; IIIC: 18.8; IIID: 2.7); IV: NR (13)	NR	NR	NR
**NSCLC**										
Abrão 2021 (29)	NSCLC Overall	Overall	1,278	NR	Male: 684 (53.5) Female: 594 (46.5)	NR	TNM 6th and 7th: Stage I: 853 (66.7); II: 425 (33.3)	NR	NR	NR
Abrão 2022 (30)	Overall patients treated with surgery or radiotherapy	Stage I	681	Mean: 64.5 (SD: 10.3)	Male: 353 (51.8) Female: 328 (48.2)	NR	NR	NR	NR	NR
Azzouqa 2019 (31)	Patients with newly diagnosed NSCLC	All stages	10,010	Median: 70	Male: 53% Female: 47%	NR	NR	NR	NR	NR

Study name	Patient population	Stage at diagnosis	Sample size (N)	Age (Year)	Gender, n (%)	Race/ ethnicity	Stage distribution	ECOG PS	Tumor histology	Comorbidities
Berglund 2012 (7)	Early stage NSCLC	Stages IA-IIB	1,828	NR	NR	NR	NR	NR	NR	NR
Cerqueira 2022 (32)	Stage III/IV at diagnosis	Stage III/IV	10,440	NR	Male: 58.5% Female: 41.5%	NR	Stage IV: 72.2%	NR	NR	NR
Ehrenstein 2022 (8)	NSCLC stage I-IIIA with EGFR mutation status-Negative	EGFR mutation status-Negative	3710	NR	Male: 1623 (43.7) Female: 2087 (56.3)	NR	Stage I: Stage IA: 1437 (38.73) Stage IB: 673 (18.14) Stage II: Stage IIA: 188 (5.07) Stage IIB: 591 (15.93) Stage III: 821 (22.12)	Status 0: 1896 (54.6) Status1-2: 1443 (41.6) Status >2: 133 (3.8) Missing: 238 (6.4)	Adenocarcinoma: 3174 (85.6) Other: 536 (14.4)	NR
	NSCLC stage I-IIIA with EGFR mutation status-Positive	EGFR mutation status-Positive	361	NR	Male: 107 (29.6) Female: 254 (70.4)	NR	Stage I: Stage IA: 166 (45.98) Stage IB: 81 (22.44) Stage II: Stage IIA: 9 (2.49) Stage IIB: 43 (11.91) Stage III: 62 (17.17)	Status 0: 225 (67.8) Status1-2: 104 (31.3) Missing: 29 (8)	Adenocarcinoma: 331 (91.7) Other: 30 (8.3)	NR
	Stage I-IIIA NSCLC	Stage I-IIIA NSCLC	8758	NR	Male: 4309 (49.2) Female: 4449 (50.8)	NR	NR	Status 0: 4243 (52.7) Status 1-2: 3451 (42.8) Missing: 700 (8)	Adenocarcinoma: 4671 (53.3) Other: 4087 (46.7)	NR
Ekman 2019 (33)	Patients with incident NSCLC	All stages	2,779	Median: 70 (range: 22–96)	Male: 48.5% Female: 51.5%	NR	Stage I: (NR) 19.3; II: (NR) 7.7; IIIA: (NR) 12.3; IIIB: (NR) 7.2; IV: (NR) 51.2	NR	Non-SCC: (NR) 70.9 SCC: (NR) 17.7 Other: (NR) 11.4	NR

Study name	Patient population	Stage at diagnosis	Sample size (N)	Age (Year)	Gender, n (%)	Race/ ethnicity	Stage distribution	ECOG PS	Tumor histology	Comorbidities
Flores 2021 (9)	NSCLC Overall	Overall	312,382	Median: 68 (range: 60-76)	Male: 166657 (53.4) Female: 145725 (46.7)	White:249062 (79.7) Black: 38201 (12.2) Others: 24345 (7.8)	NR	NR	Squamous cell carcinoma: 81,948 (26.2), Adenocarcinoma: 163,086 (52.2), Other NSCLC: 67,348 (21.6)	NR
Greystoke 2021 (34)	Patients with NSCLC with a record of systemic treatment in the SACT database of the Public Health England's CAS	Stage I-III	40,180	Median: 67	Male: 55% Female: 45%	White: Caucasian: 90%	Stage IB-IIIB: 5% Non-resected Stage IIIB: 15% Stage IV: 55%	NR	NR	NR
Jazieh 2021 (35)	Patients de novo locally advanced stage III NSCLC	Stage III	3151	Median: 63 (range: 21-92)	Male: 2411 (76.5) Female: 740 (23.5)	NR	AJCC 7th: Stage I: NA (NA) Stage III:3151 (100); IIIA: 1568 (55.9); IIIB: 1239 (55.9)	Status 0: 663 (30.3) Status 1: 1278 (58.4) Status ≥2: 246 (11.3)	Adenocarcinoma: 1665 (53.7) SCC/EC: 1134 (36.6) Other: 96 (3.1) Large cell carcinoma: 61 (2.0) Mixed: 34 (1.1) Bronchiole-alveolar: 14 (0.5)	NR

Study name	Patient population	Stage at diagnosis	Sample size (N)	Age (Year)	Gender, n (%)	Race/ ethnicity	Stage distribution	ECOG PS	Tumor histology	Comorbidities
	Stage III NSCLC – Africa and Middle East	Stage III	1046	Median: 61 (range: 24-89)	Male: 870 (83.2) Female: 176 (16.8)	NR	AJCC 7th: Stage I: NA (NA); III: 1046 (100); IIIA: 489 (58.9); IIIB: 341 (41.1); Unknown: NA (NA)	Status 0: 303 (33.9) Status 1: 489 (54.7) Status ≥2: 102 (11.4)	Adenocarcinoma: 480 (47.8) SCC/EC: 432 (43.0) Other: 9 (0.9) LCC: 27 (2.7) Mixed: 13 (1.3) Bronchiole-alveolar: 11 (1.1)	NR
	Stage III NSCLC – Asia	Stage III	1874	Median: 63 (range: 24-92)	Male: 1401 (74.8) Female: 473 (25.2)	NR	AJCC 7th: Stage I: NA (NA); III: 1874 (100); IIIA: 976 (54.7); IIIB: 808(45.3); Unknown: NA (NA)	Status 0: 295 (25.5) Status 1: 735 (63.4) Status ≥2: 129 (11.1)	Adenocarcinoma: 1039 (55.7) SCC/EC: 648 (34.7) Other: 76 (4.1) LCC: 24 (1.3) Mixed: 19 (1.0) Bronchiole-alveolar: 3 (0.2)	NR
	Stage III NSCLC – Latin America	Stage III	231	Median: 65 (range: 21-89)	Male: 140 (60.6) Female: 91 (39.4)	NR	AJCC 7th: Stage I: NA (NA); III: 231 (100); IIIA: 103 (53.4); IIIB: 90 (46.6)	Status 0: 65 (48.5) Status 1: 54 (40.3) Status ≥2: 15 (11.2)	Adenocarcinoma: 146 (64.0) SCC/EC: 54 (23.7) Other: 11 (4.8) LCC: 10 (4.4) Mixed: 2 (0.9) Bronchiole-alveolar: 0	NR
Kalilani 2022 **(36)**	Patients with advanced stage NSCLC identified from SEER database	Advanced stage (Stage III/IV)	182,693	NR	NR	NR	Stage III: 49,298 Stage IV: 133,395	NR	NR	NR

Study name	Patient population	Stage at diagnosis	Sample size (N)	Age (Year)	Gender, n (%)	Race/ ethnicity	Stage distribution	ECOG PS	Tumor histology	Comorbidities
	Patients with advanced stage NSCLC identified from Flatiron database	Advanced stage (Stage III/IV)	4,358	NR	NR	NR	Stage IIIB: 1,045 Stage IIIC: 130 Stage IV: 3,210	NR	NR	NR
Klarenbeek 2022 **(37)**	NSCLC Stage III/IV	Stage III/IV	10,306	NR	Male: 5878 (57) Female: 4428 (43)	NR	NR	Status 0: 2659 Status 1: 3159 ECOG >2: 192 Unknown: 3529	NR	NR
	NSCLC Stage III	Stage III	5038	Median: 68	NR	NR	NR	NR	Adenocarcinoma: 5696 SCC: 2742 LCC: 1580 Other: 288	NR
	NSCLC Stage III Chemoradiotherapy	Stage III	2,772	NR	Male: 1600 (58) Female: 1172 (42)	NR	NR	Status 0: 870 (31) Status 1: 858 (31); 2: 116 (5); >2: 16 (1) Unknown: 912 (32)	Adenocarcinoma: 1129 (41) SCC: 1100 (40) LCC: 506 (19) Other: 37 (0)	NR
	NSCLC Stage III Radiotherapy	Stage III	1,359	NR	Male: 861 (63) Female: 498 (37)	NR	NR	Status 0: 226 (17) Status 1: 338 (25) Status 2: 211 (16); >2: 91 (6) Unknown: 493 (46)	Adenocarcinoma: 434 (32) SCC: 572 (42) LCC: 191 (14) Other: 162 (12)	NR

Study name	Patient population	Stage at diagnosis	Sample size (N)	Age (Year)	Gender, n (%)	Race/ ethnicity	Stage distribution	ECOG PS	Tumor histology	Comorbidities
	NSCLC Stage III systemic therapy	Stage III	907	NR	Male: 513 (57) Female: 394 (43)	NR	NR	Status 0: 213 (23) Status 1: 272 (30); 2: 69 (8); >2: 11 (1) Unknown: 342 (38)	Adenocarcinoma: 414 (46) SCC: 313 (35) LCC:156 (16) Other: 24 (3)	NR
	NSCLC Stage IV	Stage IV	NR	Median: 67	NR	NR	NR	NR	NR	NR
	Stage IV systemic therapy	Stage IV	5268	NR	Male: 2904 (55) Female: 2364 (45)	NR	NR	Status 0: 1350(26) Status 1: 1691 (32); 2: 371 (7); >2: 74 (1) Unknown: 1782 (34)	Adenocarcinoma: 3719 (71) SCC: 757 (14) LCC: 727 (14) Other: 65 (1)	NR
Komiya 2020 **(38)**	Patients with unresectable stage III NSCLC with T0 status	Unresectable stage III	458	NR	NR	NR	NR	NR	NR	NR
	Patients with unresectable stage III NSCLC with T1-4 status	Unresectable stage III	84,263	NR	NR	NR	NR	NR	NR	NR
Luciano 2020 **(39)**	Advanced stage (stage III/IV) NSCLC patients	Stage III/IV	NR	NR	NR	NR	NR	NR	NR	NR

Study name	Patient population	Stage at diagnosis	Sample size (N)	Age (Year)	Gender, n (%)	Race/ ethnicity	Stage distribution	ECOG PS	Tumor histology	Comorbidities
Martin 2022 **(40)**	Patients with LA Stage III NSCLC classified according to 7th and 8th edition AJCC	Stage III	231	Median: 65 (range: 21-89)	Male: 140 (60.6) Female: 91 (39.4)	White: 69 (29.9) Asian: East Asian: 1 (0.4) Hispanic: 23 (10) Others: Mestizo: 58 (25.1) Mixed: 11 (4.8) Other: 1 (0.4) Unknown: 68 (29.4)	AJCC 7th and 8th: Stage III: Stage IIIA: 122 (52.81) Stage IIIB: 106 (45.89) Stage Unknown: 3 (1.29)	Status 0: 65 (48.5) Status 1: 54 (40.3)	Adenocarcinoma: 146 (64.0); SCC/ EC: 54 (23.7) Other: 11 (4.8) LCC: 10 (4.4) Mixed: 2 (0.9)	NR
	Patients with LA Stage IIIA NSCLC classified according to 7th edition AJCC	Stage III	193	NR	NR	NR	AJCC 7th: Stage III: Stage IIIA: 103 (53.4) Stage IIIB: 90 (46.6)	NR	NR	NR
	Patients with LA Stage IIIB NSCLC classified according to 8th edition AJCC	Stage III	35	NR	NR	NR	AJCC 8th: AJCC Stage III: Stage IIIA: 19 (54.29) Stage IIIB: 16 (45.71)	NR	NR	NR
Monteiro 2022 (10)	Overall	Early stage NSCLC (clinical stages I-IIIA)	91	Mean: 62 (range: 29-83)	Male: 40 (44) Female: 51 (56)	White: 57 (62.6) Others: Non-white: 34 (37.4)	AJCC 7th: AJCC Stage I: Stage IA: 23 (25.3); IB: 27 (29.7) Stage IIA: 15 (16.5); IIB: 11 (12.1); IIIA: 15 (16.5)	Status 0: 33 (36.3) Status 1: 58 (63.7)	Adenocarcinoma: 61 (67) SCC: 25 (27.5) Other: 5 (5.5)	NR

Study name	Patient population	Stage at diagnosis	Sample size (N)	Age (Year)	Gender, n (%)	Race/ ethnicity	Stage distribution	ECOG PS	Tumor histology	Comorbiditi es
Potter 2022 **(41)**	Overall	Stage I-IV	2010-2013: n=314,107 2014-2018: n=449,367	NR	Male: NR (2010-2013: 52.3; 2014-2018: 50.8) Female: NR (2010-2013: 47.7; 2014-2018: 49.2)	White:2010-2013: 247,136 (78.6); 2014-2018: 357,862 (79.6) Black: 2010-2013: 37107 (11.8); 2014-2018: 52816 (11.8) Asian:2010-2013: 7236 (2.3); 2014-2018: 12726 (2.8) Hispanic: 2010-2013: 17326 (5.5); 2014-2018: 17014 (3.8)	ICD-0-3: Stage I: 2010-2013: 86 609 (27.6) 2014-2018: 144 744 (32.2) Stage II: 2010-2013: 27,405 (8.7) 2014-2018: 38,538 (8.6) Stage III: 2010-2013: 63,004 (201) 2014-2018: 85,029 (18.9) Stage IV: 2010-2013: 137 089 (43.6) 2014-2018: 181 056 (40.3)	NR	Adenocarcinoma: 2010-2013: 153,268 (48.8); 2014-2018: 245,321 (54.6); SCC: 2010-2013: 88400 (28.1); 2014-2018: 126441 (28.1); LCC: 2010-2013: 7076 (2.3); 2014-2018: 7400 (1.6); ASCC: 2010-2013: 5071 (1.6); 2014-2018: 6402 (1.4); Carcinoid tumor: 2010-2013: 11211 (3.6); 2014-2018: 17563 (3.9); BC: 2010-2013:13558 (4.3); 2014-2018: 14755 (3.3)	NR
Rittberg 2021 **(42)**	Patients diagnosed with stage IV NSCLC	Stage IV	3,601	Mean: 62 (SD: NR)	Male: 53% Female: 47%	NR	Stage IV: 3,601 (100)	NR	NR	NR

Study name	Patient population	Stage at diagnosis	Sample size (N)	Age (Year)	Gender, n (%)	Race/ ethnicity	Stage distribution	ECOG PS	Tumor histology	Comorbidities
Snee 2021 (11)	Overall	Overall	3739	Mean: 72.3 (SD: 10.9) Median: 73 (range: 18-101) [IQR: 65-80]	Male: 1881 (50.3) Female: 1858 (49.7)	NR	UICC 6th: Stage I: 717 (19.2) Stage II: 434 (11.6) Stage IIIA: 469 (12.5) Stage IIIB: 337 (9.0) Stage IV: 1782 (47.7)	NR	Adenocarcinoma (NSQ): 1019 (27.3) Other NSQ: 93 (8.4) Squamous: 819 (21.9) NOS: 439 (11.7) LCC: 93 (2.5) Other: 88 (2.4) Unknown: 1281 (34.3)	NR
	NSQ NSCLC	NSQ	1112	Mean: 68.6 (SD: 11) Median: 69 (range: 31-101) [IQR: 62-77]	Male: 519 (46.7) Female: 593 (53.3)	NR	UICC 6th: Stage I: 223 (20.1) Stage II: 113 (10.2) Stage IIIA: 110 (9.9) Stage IIIB: 89 (8.0) Stage IV: 577 (51.9)	NR	Adenocarcinoma (NSQ): 1019 (27.3) Other NSQ: 93 (8.4)	NR
	SQ NSCLC	SQ	819	Mean: 70.8 (SD: 9.4) Median: 71 (range: 33-96) [IQR: 64-77]	Male: 505 (61.7) Female: 314 (38.3)	NR	UICC 6th: Stage I: 127 (15.5) Stage II: 132 (6.1) Stage IIIA: 164 (20) StageIIIB:117(14.3) Stage IV: 279 (34.1)	NR	819 (100.0)	NR

Study name	Patient population	Stage at diagnosis	Sample size (N)	Age (Year)	Gender, n (%)	Race/ ethnicity	Stage distribution	ECOG PS	Tumor histology	Comorbidities
	NSCLC NOS (not otherwise specified) NSCLC	NSCLC NOS	430	Mean: 68.9 (SD: 10.6) Median: 69 (range: 18-92) [IQR: 63-77]	Male: 220 (50.1) Female: 210 (49.9)	NR	UICC 6th: Stage I: 30 (6.8) Stage II: <40 (<9.1) Stage IIIA: 54 (12.3) Stage IIIB: <55 (<12.3) Stage IV: 263 (59.9)	NR	439 (100.0)	NR
	Other NSCLC	Other NSCLC	88	Mean: 70.1 (SD: 10.7) Median: 71 (range: 42-91) [IQR: 63-78]	Male: 49 (55.7) Female: 39 (44.3)	NR	UICC 6th: Stage I: 19 (21.6) Stage II: <13 (<14.8) Stage IIIA: 8 (9.1) Stage IIIB: <5 (<6.0) Stage IV: 47 (53.4)	NR	88 (100.0)	NR
	Clinically diagnosed NSCLC with unknown pathology	Clinically diagnosed NSCLC with unknown pathology	1281	Mean: 78 (SD: 9.3) Median: 79 (range: 43-99) [IQR: 72-85]	Male: 588 (45.9) Female: 693 (54.1)	NR	UICC 6th: Stage I: 318 (24.8) Stage II: 137 (10.7) Stage IIIA: 133 (10.4) Stage IIIB:77 (6) Stage IV: 616 (48.1)	NR	1281 (100.0)	NR
	Overall NSCLC during – 2007	Overall	255	NR	NR	NR	UICC 6th: Stage I: NR (9.8); II: NR (18); IIIA: NR (16.5); IIIB:NR (11.0); IV: NR (44.7)	NR	NR	NR

Study name	Patient population	Stage at diagnosis	Sample size (N)	Age (Year)	Gender, n (%)	Race/ ethnicity	Stage distribution	ECOG PS	Tumor histology	Comorbidities
	Overall NSCLC during – 2008	Overall	270	NR	NR	NR	UICC 6th: Stage I: NR (10); II: NR (15.6); IIIA: NR (15.6); IIIB: NR (13.3); IV: NR (45.6)	NR	NR	NR
	Overall NSCLC during – 2009	Overall	346	NR	NR	NR	UICC 6th: Stage I: NR (8.1); II: NR (16.8); III: NR (17.3); IIIB: NR (11.0); IV: NR (46.8)	NR	NR	NR
	Overall NSCLC during – 2010	Overall	342	NR	NR	NR	UICC 6th: Stage I: NR (16.4); II: NR (8.8); IIIA: NR (12.0); IIIB: NR (5.9); IV: NR (57)	NR	NR	NR
	Overall NSCLC during – 2011	Overall	347	NR	NR	NR	UICC 6th: Stage I: NR (18.4); II: NR (9.2); IIIA: NR (9.8); IIIB: NR (8.9); IV: NR (53.6)	NR	NR	NR
	Overall NSCLC during – 2012	Overall	385	NR	NR	NR	UICC 6th: Stage I: NR (19); II: NR (7); IIIA: NR (9.6); IIIB: NR (10.7); IV: NR (53.8)	NR	NR	NR
	Overall NSCLC during – 2013	Overall	372	NR	NR	NR	UICC 6th: Stage I: NR (21); II: NR (12.1); IIIA: NR (9.7); IIIB: NR (8.6); IV: NR (48.7)	NR	NR	NR

Study name	Patient population	Stage at diagnosis	Sample size (N)	Age (Year)	Gender, n (%)	Race/ ethnicity	Stage distribution	ECOG PS	Tumor histology	Comorbidities
	Overall NSCLC during – 2014	Overall	364	NR	NR	NR	UICC 6th: Stage I: NR (25.5); II: NR (10.7); IIIA: NR (11); IIIB: NR (7.4); IV: NR (45.3)	NR	NR	NR
	Overall NSCLC during – 2015	Overall	356	NR	NR	NR	UICC 6th: Stage I: NR (25.8); II: NR (10.7); IIIA: NR (11.8); IIIB: NR (8.7); IV: NR (43)	NR	NR	NR
	Overall NSCLC during – 2016	Overall	407	NR	NR	NR	UICC 6th: Stage I: NR (24.8); II: NR (11.1); IIIA: NR (11.8); IIIB: NR (7.4); IV: NR (45)	NR	NR	NR
	Overall NSCLC during – 2017 (Diagnosed up to 31 August 2017)	Overall	289	NR	NR	NR	UICC 6th: Stage I: NR (27.7); II: NR (10.4); IIIA: NR (14.9); IIIB: NR (8.0); IV: NR (39.1)	NR	NR	NR
Soares 2021 **(43)**	Overall (2012-2016 cohort)	Overall	495	Mean: 66.5 (SD: 10.1) Median: 67 (range: 34-88) [IQR: 59-74]	Male: 369 (74.5) Female: 126 (25.5)	NR	International Association for the Study of Lung Cancer 7th edition of TNM: Stage I: 174 (35.2); II: 86 (17.4); IIIA: 235 (47.5)	NR	NSQ: 291 (58.8) SQ: 167 (33.7) NSCLC NOS: 21 (4.2) Other: 16 (3.2)	NR

Study name	Patient population	Stage at diagnosis	Sample size (N)	Age (Year)	Gender, n (%)	Race/ ethnicity	Stage distribution	ECOG PS	Tumor histology	Comorbidities
	Overall (2015-2016 cohort); treated patients	Overall	199	Mean: 66.5 (SD: 9.9) Median: 68 (range: 34-86) [IQR: 59-74]	Male: 149 (74.9) Female: 50 (25.1)	NR	International Association for the Study of Lung Cancer 7th edition of TNM: Stage I: 77 (38.7); II: 33 (16.6); IIIA: 89 (44.7)	NR	NSQ: 119 (59.8) NSQs: 68 (34.2) NSCLC NOS: 7 (3.5) Other: 5 (2.5)	NR
	Stage I-II (2015-2016 cohort); treated patients; diagnosed at stage I–II	Stage I-II	102	Mean: 66.8 (SD: 9.1) Median: 67 (range: 34-85) [IQR: 60-73]	Male: 74 (72.5) Female: 28 (27.5)	NR	International Association for the Study of Lung Cancer 7th edition of TNM: Stage I: 72 (70.6); II: 30 (29.4)	NR	NSQ: 68 (66.7) SQ: 28 (27.4) NSCLC NOS: Masked Other: Masked	NR
	Stage IIIA (2015-2016 cohort); treated patients; diagnosed at stage IIIA	Stage IIIA	78	Mean: 64 (SD: 10.1) Median: 63 (range: 38-83) [IQR: 57-72]	Male: 59 (75.6) Female: 19 (24.4)	NR	International Association for the Study of Lung Cancer 7th edition of TNM: Stage IIIA: 78 (100)	NR	NSQ: 41 (52.6) SQ: 33 (42.3) NSCLC NOS: Masked Other: Masked	NR
Suipyte 2019 **(44)**	Elderly and young patients with NSCLC	Stage IV	499	NR	NR	NR	NR	NR	NR	NR
Vachani 2021 **(45)**	Newly diagnosed patients	All stages	125,330	Mean: 76.3	Male: 51% Female: 49%	NR	NR	NR	NR	HTN: 88% CPD: 52% Diabetes: 45%

Study name	Patient population	Stage at diagnosis	Sample size (N)	Age (Year)	Gender, n (%)	Race/ ethnicity	Stage distribution	ECOG PS	Tumor histology	Comorbidities
Van Dao 2022 **(46)**	Patients de novo locally advanced stage III NSCLC in Vietnam as per subgroup (7th Edition AJCC)	Stage III	149	Median: 60 (range: 26-82)	Male: 113 (75.3) Female: 37 (24.66)	NR	AJCC 7th: Stage III: Total stage III: 149 (100); IIIA: 65 (43.6); IIIB: 84 (56.4)	Status ≤1: 33 (75) Status ≥2: 11 (25)	Adenocarcinoma: 90 (62.5) Epidermoid or SCC: 38 (26.4) LCC: 9 (6.2) Other: 4 (2.8) Mixed: 3 (2.1)	NR
**RCC**										
Haas 2022a **(47)**	Patients with newly diagnosed, intermediate-high risk (pT2 N0 high grade, pT3 N0 any grade) or high-risk (pT4 N0 any grade, pT any N1 any grade) RCC	Overall	643	Mean: 75.5 (SD: NR)	Male: 61% Female: 39%	White: 86%	NR	NR	NR	NR
Haas 2022b **(48)**	Patients stratified into intermediate-high (pT2N0 high grade, pT3N0) or high risk (pT4N0, pTanyN1) RCC	Overall	274	Median: 63.5	Male: NR (66) Female: NR (34)	White: 78%	NR	NR	NR	NR

Study name	Patient population	Stage at diagnosis	Sample size (N)	Age (Year)	Gender, n (%)	Race/ ethnicity	Stage distribution	ECOG PS	Tumor histology	Comorbidities
Li 2021 **(49)**	Overall	Overall	25,145	Median: 60 [IQR: 52.00-69.00]	Male: 15792 (62.8) Female: 9353 (37.2)	White: 23559 (93.69) Asian: 1586 (6.31) Chinese: 274 (1.1) Japanese: 189 (0.8) South Asian: 164 (07) Other Asian: 959 (3.8)	NR	NR	NR	NR
	Overall – White	Overall	23,559	Median: 60 [IQR: 52.00-69.00]	Male: 14703 (62.41) Female: 8856 (37.59)	White: 23559 (100)	NR	NR	NR	NR
	Overall – Asian	Overall	1586	Median: 61 [IQR: 52.00-70.00]	Male: 1089 (68.66) Female: 497 (31.34)	Asian: Total: 1586 Chinese: 274 Japanese: 189 South Asian: 164 Other Asian: 959 (100)	NR	NR	NR	NR
	Asian – Chinese	Overall	274	Median: 64 [IQR: 54.00-72.75]	Male: 193 (70.44) Female: 81 (29.56)	NR	NR	NR	NR	NR
	Asian – Japanese	Overall	189	Median: 65 [IQR: 55.00-74.00]	Male: 135 (71.43) Female: 54 (28.57)	NR	NR	NR	NR	NR
	Asian – South Asian	Overall	164	Median: 56 [IQR: 46.00-65.00]	Male: 120 (73.17) Female: 44 (26.83)	NR	NR	NR	NR	NR

Study name	Patient population	Stage at diagnosis	Sample size (N)	Age (Year)	Gender, n (%)	Race/ ethnicity	Stage distribution	ECOG PS	Tumor histology	Comorbidities
	Asian – Other Asian	Overall	959	Median: 61 [IQR: 52.00-69.00]	Male: 641 (66.84) Female: 318 (33.16)	NR	NR	NR	NR	NR
**TNBC**										
Aly 2019a **(50)**	Metastatic TNBC – all patients	Metastatic TNBC	625	Mean: 76.8 (SD: 7.3)	Male: 0 (0) Female: 625 (100)	White: Non-Hispanic white: 454 (72) Black: Non-Hispanic black: 119 (19) Hispanic:38 (6) Others: 14 (2)	NR	NR	Tumor size, mean (SD) 115.5 (243)	NR
	Metastatic TNBC – No chemotherapy	Metastatic TNBC	308	Mean: 79 (SD: 7.7)	Male: 0 (0) Female: 308 (100)	White: Non-Hispanic white: 221 (71) Black: Non-Hispanic black: 65 (21) Hispanic:11 (3) Others: 11 (3)	NR	NR	Tumor size, mean (SD) 93.8 (203)	NR
	Metastatic TNBC – 1R only (Patients who received only one regimen)	Metastatic TNBC	161	Mean: 75.7 (SD: 6.6)	Male: 0 (0) Female: 161 (100)	White: Non-Hispanic white: 115 (71) Black: Non-Hispanic black: 30 (18)	NR	NR	Tumor size, mean (SD) 131.5 (268)	NR
	Metastatic TNBC – 2R only (Patients who received only two regimens)	Metastatic TNBC	88	Mean: 73.5 (SD: 5)	Male: 0 (0) Female: 88 (100)	White: Non-Hispanic white: 69 (78) Black: Non-Hispanic black: 11 (12)	NR	NR	Tumor size, mean (SD) 118.5 (257)	NR

Study name	Patient population	Stage at diagnosis	Sample size (N)	Age (Year)	Gender, n (%)	Race/ ethnicity	Stage distribution	ECOG PS	Tumor histology	Comorbidities
	Metastatic TNBC – 3R+ (Patients who received three or more regimens)	Metastatic TNBC	68	Mean: 73.7 (SD: 5.8)	Male: 0 (0) Female: 68 (100)	White: Non-Hispanic white: 49 (72) Black: Non-Hispanic black: 13 (19)	NR	NR	Tumor size, mean (SD) 170.5 (314)	NR
Gogate 2022 **(51)**	Early stage at diagnosis of TNBC	Early stage	707	NR	NR	NR	NR	NR	NR	NR
	Systemically treated patients with early stage TNBC	Early stage	462	Median: 59	NR	White:283 (61.3) Asian:9 (1.9) Hispanic: Hispanic or Latino: 1 (0.2) Others: Black or African American: 83 (18.0)	Tumor grade at initial diagnosis: Grade 1: 5 (1.1) Grade 2: 88 (19.0) Grade 3: 365 (79.0) Unknown: 4 (0.9)	NR	IDC: 423 (91.6) ILC: 7 (1.5) IDM and ILM: 3 (0.6) MA: 0 (0.0) Other: 26 (5.6) Unknown: 3 (0.6)	NR
Lehrberg 2021 **(52)**	Breast cancer White American	Overall	1,391	Mean: 61.6 (range: 23-91)	NR	White: 1391 (100)	NR	NR	Non-TNBC, Her2-: 905 (65.1) Non-TNBC, Her2+: 213 (15.3) TNBC: 145 (10.4) Missing: 128 (9.2)	NR

Study name	Patient population	Stage at diagnosis	Sample size (N)	Age (Year)	Gender, n (%)	Race/ ethnicity	Stage distribution	ECOG PS	Tumor histology	Comorbidities
	Breast cancer African American	Overall	907	Mean: 60.1 (range: 21-92)	NR	African American: 907 (100)	NR	NR	Non-TNBC, Her2-: 494 (54.5) Non-TNBC, Her2+: 152 (16.8) TNBC: 141 (15.5) Missing: 120 (13.2)	NR
	Breast cancer White American	Sentinel node negative	526	Mean: 61.8 (range: 31-91)	NR	White: 526 (100)	NR	NR	Non-TNBC, Her2-: 905 (65.1) Non-TNBC, Her2+: 213 (15.3) TNBC: 145 (10.4) Missing: 128 (9.2)	NR
	Breast cancer African American	Sentinel node negative	918	Mean: 60.4 (range: 27-92)	NR	African American: 918 (100)	NR	NR	Non-TNBC, Her2-: 494 (54.5) Non-TNBC, Her2+: 152 (16.8) TNBC: 141 (15.5) Missing: 120 (13.2)	NR
Schwartz 2018 (12)	AJCC Stage III	Stage III	828	NR	NR	White: 634 (76.6) Black: 117 (14.1) Hispanic: 43 (5.2) Others: 34 (4.1)	NR	NR	Ductal: 629 (76.0%) Lobular: 27 (3.3%) Mixed: 49 (5.9%) Inflammatory: 29 (3.5%) NOS: 30 (3.6%) Other: 64 (7.7%)	CCI: 0: 383 (46.3) 1: 216 (26.1) 2: 105 (12.7) >3: 124 (15)

Study name	Patient population	Stage at diagnosis	Sample size (N)	Age (Year)	Gender, n (%)	Race/ ethnicity	Stage distribution	ECOG PS	Tumor histology	Comorbiditi es
	AJCC Stage IV	Stage IV	416	NR	NR	White: 297 (71.4) Black: 86 (20.7) Hispanic: 22 (5.3) Others: 11 (2.6)	NR	NR	Ductal: 286 (68.8%) Lobular: 19 (4.6%) Mixed: 15 (3.6%) Inflammatory: 13 (3.1%) NOS: 54 (13.0%) Other: 29 (7.0%)	CCI: 0: 209 (50.2) 1: 102 (24.5) 2: 54 (13) >3: 51 (12.3)
Sieluk 2020 **(53)**	All stages (II and III)	Stages (II and III)	1569	NR	Male: <1% Female: >99%	NR	NR	NR	NR	NR
	All stages (II and III– - adjuvant cohort	Stages (II and III)	1162	NR	NR	NR	Stage II: 928 (80)	NR	NR	NR
	All stages (II and III– - neoadjuvant cohort	Stages (II and III)	94	NR	NR	NR	Stage II: 48 (51)	NR	NR	NR
	All stages (II and III– - neoadjuvant+ adjuvant cohort	Stages (II and III)	313	NR	NR	NR	Stage II: 165 (53)	NR	NR	NR
Yousefi 2017 **(54)**	TNBC overall	Overall	180	Median: 48 (range: 23-85)	Male: 0 (0) Female: 180 (100)	NR	NR	NR	NR	NR

Abbreviations: 1R: One regimen; 2R: Two regimens; 3R+: Three or more regimens; 5-FU: Fluorouracil; AI: American Indian; AJCC: American Joint Committee on Cancer; AL: Acral lentiginous; AN: Alaska native; ASCC: Adenosquamous cell carcinoma; BC: Bronchioloalveolar carcinoma; CA: Cerebrovascular accident; CaEto: Carboplatin and etoposide; CaG: Carboplatin and gemcitabine; CAS: Cancer analysis system; CCI: Charlson Comorbidity Index; CHF: Congestive heart failure; COPD: Chronic pulmonary obstructive disease; CPD: Chronic pulmonary disease; EC: Epidermoid carcinoma; ECOG PS: Eastern Cooperative Oncology Group performance score; EGFR: Estimated glomerular filtration rate; GC: Gemcitabine and cisplatin; Gem: Gemcitabine; HER2: Human epidermal growth factor receptor 2; HTN: Hypertension; ICD: International Classification of Diseases; IDC: Invasive ductal carcinoma; IDM: Infiltrating ductal mixed; ILC: Invasive lobular carcinoma; ILM: Infiltrating lobular mixed; IQR: Interquartile range; LA: Locally advanced; LCC: Large cell carcinoma; MA: Mucinous adenocarcinoma; MI: Myocardial infarction; MVAC: Methotrexate, vinblastine, doxorubicin and cisplatin; NA: Not available; NOS: Not otherwise specified; NPCR: National Program of Cancer Registries; NR: Not reported; NSCLC: Non-small cell lung cancer; NSQ: Non-squamous; RCC: Renal cell carcinoma; RD: Renal disease; SACT: Systemic anticancer therapy; SCC: Squamous cell carcinoma; SCNCC: Small-cell neuroendocrine carcinoma; SD: Standard deviation; SEER: Surveillance, Epidemiology, and End Results Program; SQ: Squamous; SRCC: Signet ring cell carcinoma; SS: Superficial spreading; TNBC: Triple negative breast cancer; TNM: Tumor (T), nodes (N), and metastases (M); UC: Urothelial cancer; UICC: Union for International Cancer Control

5 Summary of studies identified for humanistic burden review

Humanistic burden study summaries

Study name	Objective	Study design	Publication type	Country	Data source	Study setting	Time frame	QoL scale
**Melanoma**								
**Toscano 2020 (5)**	Assess the association of LOC and coping changes, with change in HRQoL in newly diagnosed breast cancer and melanoma patients at 1-, 6-, 12-, and 24-month post-diagnosis	Prospective longitudinal study	Article	France	Department of Onco-Dermatology and the Cancerology Institute	Hospital-based	November 2010 to December 2012	EORTC QLQ-C30, Brief cope questionnaire
**NSCLC**								
**Tjong 2021 Linked to: Michael 2021 (55)**	Examine moderate-to-severe symptom burden in the 12 months following diagnosis of stage IV NSCLC	Population-based study	Conference abstract	Canada	Administrative database	Database	January 2007-September 2018	ESAS questionnaire
**Bladder cancer**								
**Yu 2019 (56)**	Quantify HRQoL of patients with bladder cancer around the time of diagnosis	Semi-structured face-to-face interview	Article	UK	Participants in Bladder Cancer Prognoses Program	Multi-Center cohort study	19 December 2005 to 21 April 2011	EORTC QLQ-C30 questionnaire

Abbreviations EORTC QLQ-C30: European Organization for the Research and Treatment of Cancer Core Quality of Life Questionnaire; ESAS: Edmonton symptom assessment system ; HRQoL: Health-related quality of life; LOC: Locus-of-control; NSCLC: Non-small cell lung cancer; QoL: Quality of life; UK: United Kingdom

6 Summary of studies identified for economic burden review

Study name	Tumor Type	Objective	Study design	Publication type	Country	Data source	Study setting	Time frame	Duration of follow up	Study perspective	Cost year
**Bladder cancer**											
**Aly 2019 (57)**	Bladder (urothelial) cancer	To assess the lifetime costs by stage that will help understand the economic burden of Urothelial Carcinoma	Retrospective cohort study	Conference abstract	USA	SEER database	Nationwide population based	2004-2013	Median [IQR], months: Stage 0: 44 [23-71] Stage I: 33 [15-62] Stage II: 17 [7-39] Stage III: 17 [7-42] Stage IV: 8 [3-18]	NR	NR
**Sorup 2021 (17)**	Urothelial Cancer	To characterize treatment patterns, survival outcomes, and healthcare resource use in patients with stage IV UC in Denmark.	Population-based, retrospective cohort study	Conference abstract	Denmark	Danish Cancer Registry	Nationwide population based	January 1, 2013-December 31, 2017	Median: 10.2 months (IQR, 3.6-19.4)	NR	NR
**Head and neck cancer**											
**Singh 2021 (3)**	Oral cancer	To determine the direct healthcare costs of oral cancer at a single major tertiary provider in India	Cost of illness (prospective)	Article	India	Institutional database of the hospital	Single center	October 2019 to March 2020	NR	Healthcare provider's perspective	2018-2019

Study name	Tumor Type	Objective	Study design	Publication type	Country	Data source	Study setting	Time frame	Duration of follow up	Study perspective	Cost year
**NSCLC**											
**Buck 2015 (58)**	NSCLC	Describe patterns of systemic treatment, clinical characteristics of patients by stage, DFS, treatment patterns by starting date of adjuvant treatment from diagnosis and health care resource utilization and cost by disease stage	Retrospective observational study	Article	USA	Vector Oncology Data Warehouse	Nationwide population based	Jan 2007 to Jan2014	NR	NR	2013
**Gildea 2017 (59)**	NSCLC	Assess the wait time to diagnose NSCLC and the health care utilization and the costs of diagnosing and treating the disease based on the stage of diagnosis	Retrospective cohort study	Article	USA	Optum Research Database	Nationwide population based	Jan 2007 to Sep 2011	Mean (SD):1.1 (0.9) Years	NR	NR
**Vachani 2021 (45)**	NSCLC	Quantify the association of stage at diagnosis with OS and monthly healthcare	Retrospective observational study	Conference abstract	USA	SEER Database	Nationwide population based	2006-2015	12 months	NR	2020

Study name	Tumor Type	Objective	Study design	Publication type	Country	Data source	Study setting	Time frame	Duration of follow up	Study perspective	Cost year
		expenditures in the first year after diagnosis in patients with NSCLC.									
**TNBC**											
**Aly 2019a (50)**	TNBC	Estimate the overall survival, treatment patterns and economic burden of elderly metastatic TNBC patients.	Retrospective, observational study	Article	USA	SEER-Medicare database	National wide population based	2004 - 2011	Mean: 14.1 months	NR	2017
**Haiderali 2021 (60)**	TNBC	Examined real-world HCRU and costs in patients diagnosed with early stage (II–IIIB) TNBC	Retrospective, observational study	Article	USA	Concert AI Oncology Dataset	Nationwide population based	1 March 2008 to 31 March 2016	Median: 46.1 months (From the date of diagnosis)	Healthcare provider's perspective	NR
**Schwartz 2018 (12)**	TNBC	Identify and characterize elderly patients with advanced TNBC with respect to baseline demographics and comorbidities, treatment, including chemotherapy	Retrospective analysis	Article	USA	SEER	National wide population based	Jan 2007 to Jan 2011	Through to December 31, 2013	NR	2013

Study name	Tumor Type	Objective	Study design	Publication type	Country	Data source	Study setting	Time frame	Duration of follow up	Study perspective	Cost year
		regimens, by specific type of therapy, survival patterns, HRU and costs.									
**Sieluk 2020 (53)**	TNBC	To provide insights into patient characteristics, as well as clinical and economic outcomes for elderly patients with early stage TNBC, treated from 2010-2016 in the USA	Retrospective	Conference abstract	USA	SEER-Medicare database	Multicenter	2010 - 2015	Overall, median (range): 20.2 months (2.4-84.1)	NR	NR

Abbreviations: HRU: Healthcare resource utilization; IQR: Inter quartile range; NR: Not reported; NSCLC: Non-small cell lung cancer; OS: Overall survival; SEER: Surveillance, Epidemiology, and End Results; TNBC: Triple negative breast cancer; UC: Urothelial cancer; USA: United States of America

7 Geographic distribution of included studies

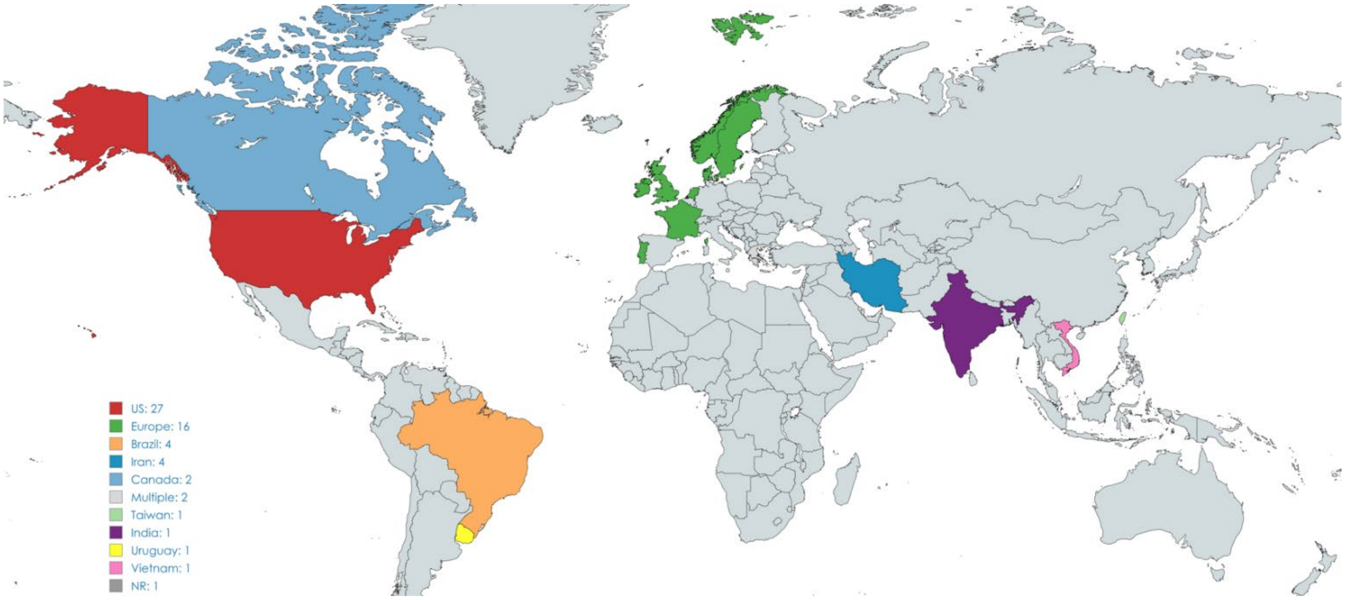

*Studies in Europe comprised of UK: 6 studies; Denmark: 4 studies; Norway: 1 study; Netherlands: 2 studies; Sweden: 1 study; Switzerland: 1 study; Portugal: 1 study; France: 1 study; Abbreviations: UK; United Kingdom; US: United States

8 Summary for median OS outcomes by tumor type and disease stage

Study name	Patient population	Stage at diagnosis	Sample size (N)	Time point	Results, months (95% CI)	p-value
**Bladder cancer**						
**Aragon-Ching 2021 (14)**	Urothelial carcinoma	Stage 0 and I	29,743	NR	Median: 80.53 (78.13–83.25)	NR
	Non-urothelial carcinoma	Stage 0 and I	561	NR	Median: 28.98 (20.3–46.78)	0.001 vs. UC
	Urothelial carcinoma	Stage II and III	29,743	NR	Median: 35.65 (33.38–38.05)	NR
	Non-urothelial carcinoma	Stage II and III	561	NR	Median: 15.75 (11.17–26.87)	Significant
	Urothelial carcinoma	Stage IV	29,743	NR	Median: 8.57 (8.05–9.10)	NR
	Non-urothelial carcinoma	Stage IV	561	NR	Median: 7 (5.62–9.26)	0.283 vs. UC
**Davies 2020 (15)**	Patients with metastatic urothelial cancer	Metastatic	2,543	NR	Median: 5.8 (5.4–6.3)	NR
**Fisher 2018 (1)**	Overall Population (including untreated patients)	Stage IV	502	From start of 1L	Median: 8.4 (7.5–9.6)	NR
		Stage IV	265	From start of 2L	Median: 4.4 (3.6–5.0)	NR
	Overall – Treated population	Stage IV	321	From start of 1L	Median: 11 (9.7–12.2)	NR
		Stage IV	147	From start of 2L	Median: 5.3 (4.5–6.5)	NR
	Overall – From diagnosis	Stage IV	508	From stage IV diagnosis	Median: 9.4 (8.3–10.7)	NR
**Omland 2021 (16)**	All patients with metastatic Urinary tract Cancer who initiated first-line chemotherapy	Metastatic	952	NR	Median: 11.7 (10.8–12.5)	NR
**Sorup 2021 (17)**	Patients with incident stage IV UC	Stage IV	620	Median: 10.2 (IQR, 3.6-19.4) months	Median: 9.4 (8.3–10.6)	NR

Study name	Patient population	Stage at diagnosis	Sample size (N)	Time point	Results, months (95% CI)	p-value
**Gastric cancer**						
**Dijksterhuis 2022 (18)**	Patients diagnosed with distant interval metastases	Metastatic	164	NR	Median: 5.5 (2.3-10.2*)	NR
**Qiu 2018 (19)**	Overall	Overall	19,022	NR	Median: 14	NR
	No metastasis	No metastasis	11,230	NR	Median: 37	NR
	With liver metastases	Metastatic	3,218	NR	Median: 4	NR
	With lung metastases		1,126	NR	Median: 3	NR
	With bone metastases		966	NR	Median: 4	NR
	With brain metastases		151	NR	Median: 3	NR
	Metastases on one site: Only liver		2,247	NR	Median: 5	NR
	Metastases on one site: Only lung		396	NR	Median: 5	NR
	Metastases on one site: Only bone		487	NR	Median: 4	NR
	Metastases on one site: Only brain		52	NR	Median: 3	NR
	Metastases on two sites: Lung and liver		428	NR	Median: 3	NR
	Metastases on two sites: Lung and bone		92	NR	Median: 4	NR
	Metastases on two sites: Lung and brain		12	NR	Median: 3	NR
	Metastases on two sites: Liver and bone		172	NR	Median: 4	NR
	Metastases on two sites: Liver and brain		18	NR	Median: 1	NR
	Metastases on two sites: Bone and brain		15	NR	Median: 2	NR
	Metastases on three sites: Lung, liver and bone		102	NR	Median: 3	NR

Study name	Patient population	Stage at diagnosis	Sample size (N)	Time point	Results, months (95% CI)	p-value
	Metastases on three sites: Lung, liver and brain		17	NR	Median: 2	NR
	Metastases on three sites: Liver, bone, brain		7	NR	Median: 3	NR
	Metastases on three sites: Lung and bone and brain		6	NR	Median: 1	NR
	Metastases on four sites: Liver, lung, bone and brain		11	NR	Median: 2	NR
	Metastases on four sites: Metastasis to other sites		3,060	NR	Median: 7	NR
	Metastases on four sites: Metastasis to unknown sites		670	NR	Median: 4	NR
**Head and Neck Cancer**						
**Amarillo 2021 (20)**	Patients who received treatment	All stages	377	6.9 years	Median: 29.16 (23–35)	NR
	Patients with localized disease who received treatment	Stage I-II	97	6.9 years	Median: 84.1	p<0.0001 vs localized
	Patients with localized disease who received treatment: TDT <40.5 days	Stage I-II	NR	6.9 years	Median: 116.3	NR
	Patients with localized disease who received treatment: TDT ≥40.5 days	Stage I-II	NR	6.9 years	Median: 55	NR
	Patients with advanced disease who received treatment	Stage III-IV	357	6.9 years	Median: 24.1	p<0.0001 vs localized

Study name	Patient population	Stage at diagnosis	Sample size (N)	Time point	Results, months (95% CI)	p-value
	Patients with advanced disease who received treatment: TDT <40.5 days	Stage III-IV	NR	6.9 years	Median: 27.6	p=0.009 vs. TDT <40.5 days
	Patients with advanced disease who received treatment: TDT ≥40.5 days	Stage III-IV	NR	6.9 years	Median: 21.1	p=0.009 vs. TDT <40.5 days
**Hochfelder 2020 (21)**	Hypopharyngeal squamous cell carcinoma patients	Stage III or IV, M0	5,272	NR	Median: 22.6 (11.1–47.3*)	NR
**Melanoma**						
**Ngo 2020 (25)**	Stage II cutaneous melanoma	Stage II	81	After relapse	NR	0.57 vs stage III
	Having multiple lesions (≥ 10) on relapse	Stage II and Stage III	NR		Median: 7.2***	NR
	Patients having just 1 lesion on relapse	Stage II and Stage III	NR		Median: 8.4***	p<0.001 vs multiple lesions on relapse
	Patients in whom >1 organ systems were involved	Stage II and Stage III	NR		Median: 8.4***	NR
	Patients in whom single organ system was involved	Stage II and Stage III	NR		Median: 21.6***	p=0.009 vs >1 organ involved
**Song 2015 (4)**	Stage IIIB/C at diagnosis	Stage IIIB/C	74	Mean: 11.3 months (SD 13.8)	Median: 24.3	NR
	Stage IV M1A at diagnosis	Stage IV M1A	212		Median: 22.3	NR
	Stage IV M1B at diagnosis	Stage IV M1B	292		Median: 11.2	NR
	Stage IV M1C at diagnosis	Stage IV M1C	1104		Median: 5.1	NR
**Tjokrowidjaja 2021 (27)**		Stage IIC	1,441	NR	Median: 46	NR
		Stage IIIC	925	NR	Median: 36	NR

Study name	Patient population	Stage at diagnosis	Sample size (N)	Time point	Results, months (95% CI)	p-value
	Survivor function of patients with melanoma to the skin who are pathologically staged; AJCC 7th edition	Stage IV	2,603	NR	Median: 9	NR
	Survivor function of patients with melanoma to the skin who are pathologically staged; AJCC 8th edition	Stage IIC	1,445	NR	Median: 46	NR
		Stage IIIC	1,730	NR	Median: 46	NR
		Stage IIID	54	NR	Median: 22	NR
		Stage IV	2,604	NR	Median: 9	NR
	Survivor function of patients with melanoma to the skin who are clinically staged; AJCC 7th edition	Stage IIC	1,441	Median: 27 months	Median: 46	NR
		Stage IV	2,603		Median: 9	NR
	Survivor function of patients with melanoma to the skin who are clinically staged; AJCC 8th edition	Stage IIC	1,446		Median: 46	NR
		Stage IV	2,604		Median: 9	NR
**NSCLC**						
**Azzouqa 2019 (31)**	Patients with newly diagnosed NSCLC having TTI ≤20 days	Stage I	NR	NR	Median: 103.4	NR
	Patients with newly diagnosed NSCLC having TTI >20 days	Stage I	NR	NR	Median: 63.9	p<0.0001 vs TTI ≤20 days
	Patients with newly diagnosed NSCLC having TTI ≤20 days	Stage II	NR	NR	Median: 72.3	NR

Study name	Patient population	Stage at diagnosis	Sample size (N)	Time point	Results, months (95% CI)	p-value
	Patients with newly diagnosed NSCLC having TTI >20 days	Stage II	NR	NR	Median: 46.8	p=0.0014 vs TTI ≤20 days
	Patients with newly diagnosed NSCLC having TTI ≤20 days	Stage III	NR	NR	Median: 30.6	NR
	Patients with newly diagnosed NSCLC having TTI >20 days	Stage III	NR	NR	Median: 28.5	p=0.118 vs TTI ≤20 days
	Patients with newly diagnosed NSCLC having TTI ≤20 days	Stage IV	NR	NR	Median: 8.3	NR
	Patients with newly diagnosed NSCLC having TTI >20 days	Stage IV	NR	NR	Median: 12.8	p<0.0001 vs TTI ≤20 days
**Cerqueira 2022 (32)**	Patients with stages III/IV NSCLC	Stages III/IV	10,440	1-Year	Median: 20.7	NR
**Ekman 2019 (33)**	Patients with incident stage I non-squamous NSCLC	Stage I	263	2012-2016	Median: Not reached	NR
	Patients with incident stage I squamous NSCLC	Stage I	65	2012-2016	Median: 52.8	NR
	Patients with incident stage II non-squamous NSCLC	Stage II	97	2012-2016	Median: 43.2	NR
	Patients with incident stage II squamous NSCLC	Stage II	35	2012-2016	Median: 23.6	NR
	Patients with incident stage IIIA non-squamous NSCLC	Stage IIIA	136	2012-2016	Median: 26.7	NR

Study name	Patient population	Stage at diagnosis	Sample size (N)	Time point	Results, months (95% CI)	p-value
	Patients with incident stage IIIA squamous NSCLC	Stage IIIA	60	2012-2016	Median: 20.4	NR
	Patients with incident stage IIIB non-squamous NSCLC	Stage IIIB	67	2012-2016	Median: 12.5	NR
	Patients with incident stage IIIB squamous NSCLC	Stage IIIB	44	2012-2016	Median: 12.9	NR
	Patients with incident stage IV non-squamous NSCLC	Stage IV	736	2012-2016	Median: 7.6	NR
	Patients with incident stage IV non-squamous NSCLC	Stage IV	123	2012-2016	Median: 6.1	NR
**Flores 2021 (9)**	Stage I/II	Stage I/II	88,179	10 years	Median: 57 (18–NR*)	NR
	Stage III	Stage III	76,422		Median: 12 (4 –34*)	NR
	Stage IV	Stage IV	140,615		Median: 5 (1 –13*)	NR
	Missing	Missing	7,166		Median: 10 (2–28*)	NR
**Greystoke 2021 (34)**	Newly diagnosed patients with NSCLC patients: Overall	Adjuvant IB-IIIA and resected IIIB	2,670	NR	Median: 75.89 (63.54–NA)	NR
		Advanced non-resected IIIB	4,970	NR	Median: 12.48 (11.99–12.94)	NR
		Advanced IV	20,470	NR	Median: 8.34 (8.21–8.48)	NR
**Jazieh 2021 (61)**	Patients de novo locally advanced stage III NSCLC	Stage III	2,619	NR	Median: 34.9 (32–38.01)	NR
**Klarenbeek 2022 (37)**	Stage III	Stage III	5038	NR	Median: 22	NR
	Stage IV	Stage IV	5268	NR	Median: 10	NR
**Luciano 2020 (39)**	Advanced stage (stage III/IV) NSCLC patients	Stage III/IV	NR	NR	Median: 6.98 (3.57–13.94*)***	NR

Study name	Patient population	Stage at diagnosis	Sample size (N)	Time point	Results, months (95% CI)	p-value
**Martin 2022 (40)**	Overall stage of disease is as per AJCC 7th edition	Stage III	168	Median follow-up: 660 days (range 7–2404)	Median: 48.6 (34.7–NC)	NR
	Stage lllA of disease is as per AJCC 7th edition	Stage lllA	89		Median: 52.5 (41.8–70.9)	NR
	Stage lllB of disease is as per AJCC 7th edition	Stage lllB	79		Median: 23.1 (16.8–52.1)	NR
**Potter 2021 (41)**	Stage I-III NSCLC patients with recorded EGFR test	Stage I	1,034	NR	Median: 72 (56.4-NC)	NR
		Stage II	560	NR	Median: 55.5 (45.9-NC)	NR
		Stage III	1,527	NR	Median: 29.5 (27.4-33.6)	NR
**Rittberg 2021 (42)**	Patients diagnosed with stage IV NSCLC with treatment era for 2006-2009	Stage IV	3,601	NR	Median: 3	NR
	Patients diagnosed with stage IV NSCLC with treatment era for 2010-2013	Stage IV	3,601	NR	Median: 2.9	NR
	Patients diagnosed with stage IV NSCLC with treatment era for 2014-2015	Stage IV	3,601	NR	Median: 2.8	p=0.082
**Snee 2021 (11)**	Patients diagnosed with NSCLC in 2007-2012	Stage I non-squamous	64	NR	Median: 55.27 (24.8–98.5*)	NR
	Patients diagnosed with NSCLC in 2013-2017		159	NR	Median: Not reached (34.2–NR)	NR

Study name	Patient population	Stage at diagnosis	Sample size (N)	Time point	Results, months (95% CI)	p-value
	Patients diagnosed with NSCLC in 2007-2012	Stage I squamous	58	NR	Median: 37.28 (18.5–66.8*)	NR
	Patients diagnosed with NSCLC in 2013-2017		69	NR	Median: 51.13 (32.6–NA*)	NR
	Patients diagnosed with NSCLC in 2007-2012	Stage I NSCLC clinically diagnosed with unknown pathology	128	NR	Median: 16.72 (5.8–33.1*)	NR
	Patients diagnosed with NSCLC in 2013-2017		189	NR	Median: 20.9 (8.0–40.3*)	NR
	Patients diagnosed with NSCLC in 2007-2012	Stage II non-squamous	61	NR	Median: 34.27 (10.6–80.0*)	NR
	Patients diagnosed with NSCLC in 2013-2017		51	NR	Median: 26.43 (10.2–58.0*)	NR
	Patients diagnosed with NSCLC in 2007-2012	Stage II squamous	58	NR	Median: 17.2 (8.6–58.2*)	NR
	Patients diagnosed with NSCLC in 2013-2017		74	NR	Median: 19.87 (7.2–53.9*)	NR
	Patients diagnosed with NSCLC in 2007-2012	Stage II NSCLC clinically diagnosed with unknown pathology	81	NR	Median: 8.93 (2.9–16.8*)	NR
	Patients diagnosed with NSCLC in 2013-2017		55	NR	Median: 11.33 (5.4–26.9*)	NR
	Stage IIIA, non-squamous (patients diagnosed with NSCLC in 2007-2012)	Stage IIIA, non-squamous	51	NR	Median: 9.93 (6.5–38.6*)	NR

Study name	Patient population	Stage at diagnosis	Sample size (N)	Time point	Results, months (95% CI)	p-value
	Stage IIIA, non-squamous Stage II NSCLC without pathological diagnosis (patients diagnosed with NSCLC in 2013-2017)		57	NR	Median: 23.97 (10.6–NA*)	NR
	Patients diagnosed with NSCLC in 2007-2012	Stage IIIA, squamous	91	NR	Median: 10.73 (4.4–21.1*)	NR
	Patients diagnosed with NSCLC in 2013-2017		72	NR	Median: 14.5 (8.4–36.0*)	NR
	Stage III NSCLC without pathological diagnosis (patients diagnosed with NSCLC in 2007-2012)	Stage III NSCLC	77	NR	Median: 5.77 (1.5–11.0*)	NR
	Stage III NSCLC clinically diagnosed with unknown pathology (patients diagnosed with NSCLC in 2013-2017)		57	NR	Median: 5 (1.9–7.5*)	NR
	Patients diagnosed with NSCLC in 2007-2012	Stage IIIB-IV, non-squamous	345	NR	Median: 4.07 (1.3–10.05*)	NR
	Patients diagnosed with NSCLC in 2013-2017		321	NR	Median: 5 (1.7–12.9*)	NR
	Patients diagnosed with NSCLC in 2007-2012	Stage IIIB-IV, squamous	229	NR	Median: 5.33 (2.2–12.0*)	NR

Study name	Patient population	Stage at diagnosis	Sample size (N)	Time point	Results, months (95% CI)	p-value
	Patients diagnosed with NSCLC in 2013-2017		167	NR	Median: 4.8 (2.4–11.9*)	NR
	Stage IIIB-IV NSCLC without pathological diagnosis (patients diagnosed with NSCLC in 2007-2012)	Stage IIIB-IV NSCLC clinically diagnosed with unknown pathology	370	NR	Median: 1.23 (0.4–3.2*)	NR
	Stage IIIB-IV NSCLC (patients diagnosed with NSCLC in 2013-2017)		323	NR	Median: 1.23 (0.4–3.4*)	NR
**Soares 2021 (43)**	Stage I (diagnosed with NSCLC between 2012-2016)	Stage I	174	NA	Median: Not reached (29.3 – Not reached*)	NR
	Stage II (diagnosed with NSCLC between 2012-2016)	Stage II	86	NA	Median: 23 (10.2–Not reached*)	NR
	Stage III A (diagnosed with NSCLC between 2012-2016)	Stage III A	235	NA	Median: 21.7 (9.5–52.7*)	NR
**Suipyte 2019 (44)**	Elderly patients (>70 years) diagnosed between 2005-2007	Stage IV	NR	NR	Median: 3.7	NR
	Elderly patients (>70 years) diagnosed between 2015-2016	Stage IV	NR	NR	Median: 5.3	NR
	Young patients (<70 years) diagnosed between 2005-2007	Stage IV	NR	NR	Median: 7.8	NR
	Young patients (<70 years) diagnosed between 2015-2016	Stage IV	NR	NR	Median: 12.1	NR

Study name	Patient population	Stage at diagnosis	Sample size (N)	Time point	Results, months (95% CI)	p-value
**Van Dao 2022 (46)**	Patients de novo locally advanced stage III NSCLC	Stage III	139	NR	Median: 25.7 (19.98–42.61)	NR
	Patients de novo locally advanced stage IIIA NSCLC	Stage IIIA	59	NR	Median: 28.2 (24.15–NC)	NR
	Patients de novo locally advanced stage IIIB NSCLC	Stage IIIB	80	NR	Median: 20 (13.01–42.61)	NR
**RCC**						
**Haas 2022b (48)**	Patients with intermediate-high risk RCC	Localized	NR	Median follow-up: 49.5 months	Median: 83.4	NR
	Patients with high risk RCC		NR		Median: 78.4	NR
	Patients without recurrence among intermediate-high risk and high risk RCC		NR		Median: 93.8	NR
	Patients with recurrence among intermediate-high risk and high risk RCC		NR		Median: 69.6	NR
**Haas 2022a (47)**	Newly diagnosed, recurrent patients with intermediate-high risk RCC	Localized	259	Median follow-up: 23 months	Median: 55.68	NR
	Newly diagnosed, recurrent patients with T3G1-G2 RCC (intermediate-high risk RCC subgroup): after recurrence		101		Median: 77.64	NR

Study name	Patient population	Stage at diagnosis	Sample size (N)	Time point	Results, months (95% CI)	p-value
	Newly diagnosed, recurrent patients with T3G3 RCC (intermediate-high risk RCC subgroup): after recurrence		111		Median: 55.68	NR
	Newly diagnosed, recurrent patients with T3G4 RCC (intermediate-high risk RCC subgroup): after recurrence		41		Median: 23.4	NR
**TNBC**						
**Schwartz 2018 (12)**	All AJCC stage III elderly patients	Stage III	828	Survival from diagnosis	Median: 30 (27–34)	NR
	Overall (Stage III/IV) elderly patients	Stage III and IV	1244	Survival from diagnosis	Median: 18 (16–20)	NR
	All AJCC stage IV elderly patients	Stage IV	416	Survival from diagnosis	Median: 5 (4–7)	NR
**Sieluk 2020 (53)**	Patients who initiated systemic neoadjuvant and adjuvant (including chemotherapy and radiation) therapy	Stage II/III	313	NR	Median: 77.6 (55.6–NR)	NR
		Stage II	165		Median: 77.6 (77.6–NR)	NR
		Stage III	148		Median: 37.8 (27.3–56.1)	NR
**Aly 2019a (50)**	Metastatic TNBC - all patients	Metastatic	625	Mean: 17.1 months	Median: 7 (6.2–8.1)	NR

Abbreviations: 1L: First line; 2L: Second line; AJCC: American Joint Committee on Cancer; CI: Confidence interval; IQR: Inter quartile range; NA: Not available; NC: Not calculable; NR: Not reported; NSCLC: Non-small cell lung cancer; RCC: Renal cell carcinoma; SD: Standard deviation; TDT: Time between diagnosis and treatment; TNBC: Triple negative breast cancer; TTI: Time to treatment initiation; UC: Urothelial cancer

9 Summary of reported OS rates

Overall survival rates in patients included in studies

Study name	Patient population	Stage at diagnosis	Sample size (N)	Time point / Variable	Survival rate results	p-value
**Bladder cancer**						
**Amiri 2020 (13)**	Bladder cancer	TNM stage I	210	5-years	67%	<0.001
		TNM stage II	74		45%	
		TNM stage III	29		15%	
		Stage I (TNM)	210	NR	Mean rate per month: 58.5%; 95% CI: 55–62	NR
		Stage II (TNM)	74		Mean rate per month: 44.5%; 95% CI: 37.2–51.7	
		Stage III (TNM)	29		Mean rate per month: 21.7%; 95% CI: 12.9–30.4	
		Stage IV (TNM)	8		Mean rate per month: 21.3%; 95% CI: 8.9–33.6	
**Fisher 2018 (1)**	Overall (The time origin was the start of therapy within 1L or 2L for those who did not receive treatment)	Stage IV	502	From start of 1L	n (%): 425 (84.7); 95% CI: 7.5–9.6	NR
		Stage IV	265	From start of 2L	n (%): 236 (89.1); 95% CI: 3.6–5.0	NR
	Overall – Treated (The time origin was the start of therapy within 1L or 2L)	Stage IV	321	From start of 1L	n (%): 263 (81.9); 95% CI: 9.7–12.2	NR
		Stage IV	147	From start of 2L	n (%): 130 (88.4); 95% CI: 4.5–6.5	NR
**Sorup 2021 (17)**	Patients with incident urothelial cancer	Stage IV	620	1-year	40.4%; 95% CI: 36.4–44.4	NR
		Stage IV	620	2-year	23.6%; 95% CI: 20.1–27.4	NR
**Head and neck cancer**						
**Ho 2019 (2)**	Oral cavity cancer	Stage 0	275	3-year	90.8%	NR
		Stage I	10,383		88%	NR
		Stage II	6627		79.8%	NR
		Stage III	3954		79.8%	NR
		Stage IV	13,134		45.4%	NR
		Stage 0	275	5-year	83.9%	NR
		Stage I	10,383		82.1%	NR

Study name	Patient population	Stage at diagnosis	Sample size (N)	Time point / Variable	Survival rate results	p-value
		Stage II	6627		72.7%	NR
		Stage III	3954		72.7%	NR
		Stage IV	13,134		38%	NR
		Stage 0	275		97.1%	NR
		Stage I	10,383		92.3%	NR
		Stage II	6627		84.9%	NR
		Stage III	3954		74.6%	NR
		Stage IV	13,134		52.1%	NR
		Stage 0	275	3-year	94.5%	NR
		Stage I	10,383		89.0%	NR
		Stage II	6627		80.4%	NR
		Stage III	3954		68.3%	NR
		Stage IV	13,134		45.9%	NR
		Stage 0	275	5-year	90.8%	NR
		Stage I	10,383		88%	NR
		Stage II	6627		79.8%	NR
		Stage III	3954		79.8%	NR
		Stage IV	13,134		45.4%	NR
**Neto 2021 (22)**	Oropharynx cancer	Stage I	17	1-year	100.0	NR
		Stage II	29	1-year	89.1	NR
		Stage III	54	1-year	94.3	NR
		Stage IV A	319	1-year	92.0	NR
		Stage IV B	19	1-year	94.1	NR
		Stage IV C	25	1-year	59.4	NR
		Stage I	17	3-year	93.3	NR
		Stage II	29	3-year	85.4	NR
		Stage III	54	3-year	90.2	NR
		Stage IV A	319	3-year	81.6	NR
		Stage IV B	19	3-year	60.1	NR
		Stage IV C	25	3-year	50.9	p <0.001 vs. stage I
		Stage I	279	1-year	93.4	NR
		Stage II	94	1-year	91.4	NR

Study name	Patient population	Stage at diagnosis	Sample size (N)	Time point / Variable	Survival rate results	p-value
		Stage III	65	1-year	90.2	NR
		Stage IV	25	1-year	59.4	NR
		Stage I	279	3-year	85.7	NR
		Stage II	94	3-year	80.5	NR
		Stage III	65	3-year	71.5	NR
		Stage IV	25	3-year	50.9	p <0.001 vs. stage I
**Sargeran 2009 (24)**	Overall Lip cancer	Overall	82	1-Year	91%	NR
			82	2-Year	86%	NR
			82	5-year	62%	NR
	Early-stage Lip cancer	Stage I at diagnosis	35	1-Year	97%	p<0.001 vs. stage IV
			35	2-Year	91%	
			35	5-year	81%	
		Stage II at diagnosis	17	1-Year	93%	
			17	2-Year	87%	
			17	5-year	75%	
	Advanced stage Lip cancer	Stage III at diagnosis	11	1-Year	81%	
			11	2-Year	73%	
			11	5-year	45%	
		Stage IV at diagnosis	9	1-Year	67%	
**Sargeran 2008 (23)**	Oral cancer	Overall	470	The mean (SD) follow-up: 32 months (26), range 0–116.	n (%): 80 (17)	NR
		Overall	470	1-Year	77%	NR
		Overall	470	2-Year	57%	NR
		Overall	470	5-year	30%	NR
		Stage I	92	1-Year	93%	NR
		Stage I	92	2-Year	80%	NR
		Stage I	92	5-year	51%	NR
		Stage II	73	1-Year	91%	NR
		Stage II	73	2-Year	80%	NR

Study name	Patient population	Stage at diagnosis	Sample size (N)	Time point / Variable	Survival rate results	p-value
		Stage II	73	5-year	44%	NR
		Stage III	70	1-Year	77%	NR
		Stage III	70	2-Year	50%	NR
		Stage III	70	5-year	13%	NR
		Stage IV	167	1-Year	45%	NR
		Stage IV	167	2-Year	22%	NR
		Stage IV	167	5-year	12%	NR
**Melanoma**						
**Ramond 2021 (26)**	All patients with melanoma	Stage I, II, III and IV	184,864	1 year	94%	NR
	Patients with stage I melanoma	Stage I	NR	1 year	99%	NR
	Patients with stage II melanoma	Stage II	NR	1 year	94%	NR
	Patients with stage III melanoma	Stage III	NR	1 year	90%	NR
	Patients with stage IV melanoma	Stage IV	NR	1 year	48%	NR
	All patients with melanoma (Relative survival) [a]	Stage I, II, III and IV	184,864	1 year	97%	NR
**Song 2015 (4)**	Stage IIIB/C at diagnosis	Stage IIIB/C	74	1-year	67.20%	NR
	Stage IV M1A at diagnosis	Stage M1A	212	1-year	64.50%	NR
	Stage IV M1B at diagnosis	Stage M1B	292	1-year	43.80%	NR
	Stage IV M1C at diagnosis	Stage M1C	1104	1-year	22.30%	NR
	Stage IIIB/C at diagnosis	Stage IIIB/C	74	2-year	42.90%	NR
	Stage IV M1A at diagnosis	Stage M1A	212	2-year	40.40%	NR
	Stage IV M1B at diagnosis	Stage M1B	292	2-year	23.40%	NR
	Stage IV M1C at diagnosis	Stage M1C	1104	2-year	8.90%	NR
	Stage IIIB/C at diagnosis	Stage IIIB/C	74	3-year	32.10%	NR
	Stage IV M1A at diagnosis	Stage M1A	212	3-year	26.40%	NR
	Stage IV M1B at diagnosis	Stage M1B	292	3-year	13.80%	NR
	Stage IV M1C at diagnosis	Stage M1C	1104	3-year	4.70%	NR
**Tjokrowidjaja 2021 (27)**	Survivor function for AJCC 7th edition among pathologically staged patients	Stage IA	26,944	3-Year	n (%): 10627 (97); 95% CI: 96–97	NR
		Stage IA	26,944	5-Year	n (%): 2806 (94); 95% CI: 94–95	NR
		Stage IB	18,507	3-Year	n (%): 7715 (95); 95% CI: 94–95	NR
		Stage IB	18,507	5-Year	n (%): 2116 (90); 95% CI: 90–91	NR
		Stage IIA	4117	3-Year	n (%): 1600 (87); 95% CI: 86–88	NR
		Stage IIA	4117	5-Year	n (%): 432 (78); 95% CI: 75–80	NR

Study name	Patient population	Stage at diagnosis	Sample size (N)	Time point / Variable	Survival rate results	p-value
		Stage IIB	2829	3-Year	n (%): 937 (76); 95% CI: 74–78	NR
		Stage IIB	2829	5-Year	n (%): 242 (64); 95% CI: 61–66	NR
		Stage IIC	1441	3-Year	n (%): 335 (57); 95% CI: 54–60	NR
		Stage IIC	1441	5-Year	n (%): 74 (39); 95% CI: 35–44	NR
		Stage IIIA	1160	3-Year	n (%): 438 (86); 95% CI: 83–89	NR
		Stage IIIA	1160	5-Year	n (%): 140 (79); 95% CI: 75–82	NR
		Stage IIIB	1463	3-Year	n (%): 446 (69); 95% CI: 66–72	NR
		Stage IIIB	1463	5-Year	n (%): 98 (57); 95% CI: 52–61	NR
		Stage IIIC	925	3-Year	n (%): 194 (50); 95% CI: 46–54	NR
		Stage IIIC	925	5-Year	n (%): 35 (38); 95% CI: 33–43	NR
		Stage IV	2603	3-Year	n (%): 247 (24); 95% CI: 22–26	NR
		Stage IV	2603	5-Year	n (%): 61 (20); 95% CI: 18–22	NR
	Survivor function for AJCC 8th edition among pathologically staged patients	Stage IA	38,344	3-Year	n (%): 15333 (96); 95% CI: 96–96	NR
		Stage IA	38,344	5-Year	n (%): 4074 (93); 95% CI: 93–94	NR
		Stage IB	7099	3-Year	n (%): 3008 (94); 95% CI: 93–95	NR
		Stage IB	7099	5-Year	n (%): 848 (89); 95% CI: 88–90	NR
		Stage IIA	4116	3-Year	n (%): 1600 (87); 95% CI: 86–88	NR
		Stage IIA	4116	5-Year	n (%): 432 (78); 95% CI: 75–80	NR
		Stage IIB	2833	3-Year	n (%): 937 (76); 95% CI: 74–78	NR
		Stage IIB	2833	5-Year	n (%): 242 (64); 95% CI: 61–66	NR
		Stage IIC	1445	3-Year	n (%): 335 (57); 95% CI: 54–60	NR
		Stage IIC	1445	5-Year	n (%): 74 (39); 95% CI: 34–44	NR
		Stage IIIA	855	3-Year	n (%): 333 (87); 95% CI: 84–90	NR
		Stage IIIA	855	5-Year	n (%): 100 (82); 95% CI: 78–85	NR
		Stage IIIB	909	3-Year	n (%): 306 (81); 95% CI: 78–84	NR
		Stage IIIB	909	5-Year	n (%): 79 (69); 95% CI: 64–74	NR
		Stage IIIC	1730	3-Year	n (%): 430 (56); 95% CI: 53–59	NR
		Stage IIIC	1730	5-Year	n (%): 94 (44); 95% CI: 40–48	NR
		Stage IIID	54	3-Year	n (%): 10 (30); 95% CI: 17–45	NR
		Stage IIID	54	5-Year	n (%): 0 (0)	NR
		Stage IV	2604	3-Year	n (%): 247 (24); 95% CI: 22–26	NR
		Stage IV	2604	5-Year	n (%): 61 (20); 95% CI: 18–22	NR
**Wilson 2019 (6)**	Overall	NA	304	Median (post metastasis): 25	n (%): 75 (24.7)	NR

Study name	Patient population	Stage at diagnosis	Sample size (N)	Time point / Variable	Survival rate results	p-value
				(range: 0.5 to 167.8) months		
	Stage I at diagnosis (clinically stage AJCC 7)	Stage I	96	Median (post metastasis): 22 (range: 5.1–155.8) months	n (%): 29 (30.2)	NR
	Stage II at diagnosis (clinically stage AJCC 7)	Stage II	99	Median (post metastasis): 37.5 (range: 5.5–141.3) months	n (%): 23 (23.2)	NR
	Stage III at diagnosis (clinically stage AJCC 7)	Stage III	109	Median (post metastasis): 27.7 (range: 0.5–167.8) months	n (%): 23 (23.2)	NR
**Winge-Main 2020 (28)**	Patients with cutaneous melanoma	Stage IIB	4,339	3-year	74.50%	NR
		Stage IIC	4,339	3-year	48.70%	NR
		Stage IIIA	4,339	3-year	91.50%	NR
		Stage IIIB	4,339	3-year	76.70%	NR
		Stage IIIC	4,339	3-year	63.10%	NR
		Stage IIID	4,339	3-year	55.40%	NR
		Stage IV	4,339	3-year	63.70%	NR
**NSCLC**						
**Abrão 2021 (29)**	Stage I	Stage I	853	5-year	n (%): 755 (45.6)	NR
	Stage II	Stage II	425	5-year	n (%): 387 (27.5)	<0.001
**Abrão 2022 (30)**	Stage I/II	Early stage	681	NR	3-years: 68.8% 5-years: 58.1%	NR
**Berglund 2012[b] (7)**	Socioeconomic quintile-Q1 (affluent)	Early stage (Stages IA-IIB at diagnosis)	1828	3-year (cumulative data)	50%	NR
	Socioeconomic quintile-Q5 (deprived)				39%	NR
**Cerqueira 2022 (32)**	Patients with stages III/IV NSCLC	Stages III/IV	10,440	1-Year	57.1%	NR
**Ehrenstein 2022 (8)**	EGFR mutation status negative	Stage IIA	188	6-Year	n (%): <5 (NR)	NR
	EGFR mutation status-Positive	Stage IB	81	6-Year	n (%): <5 (NR)	NR
		Stage IIA	9	4-Year	n (%): <5 (NR)	NR
		Stage IIA	9	5-Year	n (%): <5 (NR)	NR
		Stage IIB	43	5-Year	n (%): <5 (NR)	NR

Study name	Patient population	Stage at diagnosis	Sample size (N)	Time point / Variable	Survival rate results	p-value
		Stage IIB	43	6-Year	n (%): <5 (NR)	NR
		Stage IIIA	62	5-Year	n (%): <5 (NR)	NR
		Stage IIIA	62	6-Year	n (%): <5 (NR)	NR
**Kalilani 2022 (36)**	Patients identified from SEER database	Stage III	49,298	1 year	55.1%	NR
			49,298	3 years	26.3%	NR
			49,298	5 years	17.5%	NR
		Stage IV	133,395	1 year	25.8%	NR
			133,395	3 years	7.4%	NR
			133,395	5 years	4%	NR
	Patients identified from Flatiron database	Stage III	1,175	1 year	72.5%	NR
			1,175	3 years	36.4%	NR
		Stage IV	3,210	1 year	65.9%	NR
			3,210	3 years	24.6%	NR
**Klarenbeek 2022 (37)**	Stage III	Stage III	NR	1-year, 3-year, 5-year	n (%): NR (1-year: 71%, 3-year: 37%, 5-year: 25%);	NR
	Stage IV	Stage III	5268		n (%): NR (1-year: 44%, 3-year: 16%, 5-year: 9%);	NR
**Komiya 2020 (38)**	Patients with unresectable stage III NSCLC with T0 status	Unresectable stage III	458	5-year	30.5%	<0.0001
	Patients with unresectable stage III NSCLC with T1-4 status		84,263	5-year	12.7%	NR
	Patients with unresectable stage III NSCLC with T0 status		458	5-year	35.3%	NR
	Patients with unresectable stage III NSCLC with T1-4 status		84,263	5-year	13.5%	NR
**Monteiro 2022 (10)**	Stage I	Stage I	50	5 years OS	68.9%; 95% CI: 53.5–80.0	0.001
	Stage II	Stage II	26	5 years OS	61%; 95% CI: 39.6–76.9	NR
	Stage IIIA	Stage IIIA	15	5 years OS	25%; 95% CI: 6.9–48.8	NR
**Potter 2022[c] (41)**	Patients with stage I NSCLC	Stage I	NR	5-year	59.5%; 95% CI: 59.3% to 59.7%	NR
	Patients with unknown stage NSCLC	Unknown	NR	5-year	34.1%; 95% CI: 33.8% to 34.5%	<0.001 vs stage I
	Patients in low screening state	Stage I	NR	2010 to 2017	Annual percent change: 5.9%	NR
	Patients in high screening state	Stage I	NR	2010 to 2014	Annual percent change: 2.2%; 95% CI: −4.7% to 9.7%;	0.39

Study name	Patient population	Stage at diagnosis	Sample size (N)	Time point / Variable	Survival rate results	p-value
	Patients in high screening state	Stage I	NR	2014 to 2017	Annual percent change: 13.4%; 95% CI: 1.4% to 26.9%	0.04
Snee 2021 (11)	Patients diagnosed with NSCLC in 2007-2012	Stage I non-squamous	58	1-year	91%; 95% CI: 84–98	NR
	Patients diagnosed with NSCLC in 2013-2017		123	1-year	90%; 95% CI: 86–95	NR
	Patients diagnosed with NSCLC in 2007-2012		37	3-year	58%; 95% CI: 47–71	NR
	Patients diagnosed with NSCLC in 2013-2017		44	3-year	72%; 95% CI: 64–81	NR
	Patients diagnosed with NSCLC in 2007-2012		25	5-year	43%; 95% CI: 32–57	NR
	Patients diagnosed with NSCLC in 2013-2017		<6	5-year	51%; 95% CI: 37–72	NR
	Patients diagnosed with NSCLC in 2007-2012	Stage I squamous	46	1-year	79%; 95% CI: 70–90	NR
	Patients diagnosed with NSCLC in 2013-2017		55	1-year	85%; 95% CI: 77–94	NR
	Patients diagnosed with NSCLC in 2007-2012		29	3-year	5%; 95% CI: 39–65	NR
	Patients diagnosed with NSCLC in 2013-2017		24	3-year	73%; 95% CI: 61–86	NR
	Patients diagnosed with NSCLC in 2007-2012		16	5-year	28%; 95% CI: 18–42	NR
	Patients diagnosed with NSCLC in 2013-2017		<6	5-year	32%; 95% CI: 13–81	NR
	Patients diagnosed with NSCLC in 2007-2012	Stage I NSCLC clinically diagnosed with unknown pathology	76	1-year	59%; 95% CI: 51–69	NR
	Patients diagnosed with NSCLC in 2013-2017		97	1-year	66%; 95% CI: 60–74	NR
	Patients diagnosed with NSCLC in 2007-2012		25	3-year	20%; 95% CI: 14–28	NR
	Patients diagnosed with NSCLC in 2013-2017		17	3-year	27%; 95% CI: 19–38	NR
	Patients diagnosed with NSCLC in 2007-2012		11	5-year	9%; 95% CI: 05–16	NR

Study name	Patient population	Stage at diagnosis	Sample size (N)	Time point / Variable	Survival rate results	p-value
	Patients diagnosed with NSCLC in 2013-2017		<6	5-year	10%; 95% CI: 04–28	NR
	Patients diagnosed with NSCLC in 2007-2012	Stage II non-squamous	41	1-year	68%; 95% CI: 58–81	NR
	Patients diagnosed with NSCLC in 2013-2017		30	1-year	72%; 95% CI: 60–85	NR
	Patients diagnosed with NSCLC in 2007-2012		27	3-year	45%; 95% CI: 34–60	NR
	Patients diagnosed with NSCLC in 2013-2017		12	3-year	42%; 95% CI: 30–61	NR
	Patients diagnosed with NSCLC in 2007-2012		18	5-year	31%; 95% CI: 21–46	NR
	Patients diagnosed with NSCLC in 2013-2017		0	5-year	0%; 95% CI: NA	NR
	Patients diagnosed with NSCLC in 2007-2012	Stage II squamous	36	1-year	62%; 95% CI: 51–76	NR
	Patients diagnosed with NSCLC in 2013-2017		45	1-year	66%; 95% CI: 55–.77	NR
	Patients diagnosed with NSCLC in 2007-2012		18	3-year	31%; 95% CI: 21–46	NR
	Patients diagnosed with NSCLC in 2013-2017		15	3-year	42%; 95% CI: 31–57	NR
	Patients diagnosed with NSCLC in 2007-2012		14	5-year	24%; 95% CI: 15–38	NR
	Patients diagnosed with NSCLC in 2013-2017		0	5-year	0%; 95% CI: NA	NR
	Patients diagnosed with NSCLC in 2007-2012	Stage II NSCLC clinically diagnosed with unknown pathology	27	1-year	33%; 95% CI: 24–.45	NR
	Patients diagnosed with NSCLC in 2013-2017		25	1-year	50%; 95% CI: 38–65	NR
	Patients diagnosed with NSCLC in 2007-2012		11	3-year	14%; 95% CI: 08–24	NR
	Patients diagnosed with NSCLC in 2013-2017		<6	3-year	9%; 95% CI: 03–31	NR
	Patients diagnosed with NSCLC in 2007-2012		7	5-year	9%; 95% CI: 04–18	NR

Study name	Patient population	Stage at diagnosis	Sample size (N)	Time point / Variable	Survival rate results	p-value
	Patients diagnosed with NSCLC in 2013-2017		0	5-year	0%; 95% CI: NA	NR
	Patients diagnosed with NSCLC in 2007-2012	Stage IIIA, non-squamous Stage II NSCLC without pathological diagnosis	23	1-year	45%; 95% CI: 33–61	NR
	Patients diagnosed with NSCLC in 2013-2017		35	1-year	74%; 95% CI: 63–87	NR
	Patients diagnosed with NSCLC in 2007-2012		14	3-year	27%; 95% CI: 18–43	NR
	Patients diagnosed with NSCLC in 2013-2017		<6	3-year	35%; 95% CI: 22–58	NR
	Patients diagnosed with NSCLC in 2007-2012		9	5-year	18%; 95% CI: 10–32	NR
	Patients diagnosed with NSCLC in 2013-2017		0	5-year	0%; 95% CI: NA	NR
	Patients diagnosed with NSCLC in 2007-2012	Stage IIIA, squamous	41	1-year	45%; 95% CI: 36–57	NR
	Patients diagnosed with NSCLC in 2013-2017		29	1-year	54%; 95% CI: 43–68	NR
	Patients diagnosed with NSCLC in 2007-2012		12	3-year	13%; 95% CI: 08–22	NR
	Patients diagnosed with NSCLC in 2013-2017		<6	3-year	21%; 95% CI: 10–42	NR
	Patients diagnosed with NSCLC in 2007-2012		10	5-year	11%; 95% CI: 06–20	NR
	Patients diagnosed with NSCLC in 2013-2017		0	5-year	0%; 95% CI: NA	NR
	Patients diagnosed with NSCLC in 2007-2012	Stage III NSCLC clinically diagnosed with unknown pathology	16	1-year	21%; 95% CI: 13–32	NR
	Patients diagnosed with NSCLC in 2013-2017		7	1-year	20%; 95% CI: 12–36	NR
	Patients diagnosed with NSCLC in 2007-2012		<6	3-year	05%; 95% CI: 02–13	NR
	Patients diagnosed with NSCLC in 2013-2017		0	3-year	0%; 95% CI: NA	NR
	Patients diagnosed with NSCLC in 2007-2012		0	5-year	0%; 95% CI: NA	NR

Study name	Patient population	Stage at diagnosis	Sample size (N)	Time point / Variable	Survival rate results	p-value
	Patients diagnosed with NSCLC in 2013-2017		0	5-year	0%; 95% CI: NA	NR
	Patients diagnosed with NSCLC in 2007-2012	Stage IIIB-IV, non-squamous	68	1-year	20%; 95% CI: 16–25	NR
	Patients diagnosed with NSCLC in 2013-2017		72	1-year	26%; 95% CI: 21–31	NR
	Patients diagnosed with NSCLC in 2007-2012		19	3-year	06%; 95% CI: 04–09	NR
	Patients diagnosed with NSCLC in 2013-2017		8	3-year	06%; 95% CI: 04–11	NR
	Patients diagnosed with NSCLC in 2007-2012		11	5-year	03%; 95% CI: 02–07	NR
	Patients diagnosed with NSCLC in 2013-2017		0	5-year	0%; 95% CI: NA	NR
	Patients diagnosed with NSCLC in 2007-2012	Stage IIIB-IV, squamous	56	1-year	24%; 95% CI: 19–31	NR
	Patients diagnosed with NSCLC in 2013-2017		37	1-year	25%; 95% CI: 19–32	NR
	Patients diagnosed with NSCLC in 2007-2012		12	3-year	05%; 95% CI: 03–09	NR
	Patients diagnosed with NSCLC in 2013-2017		6	3-year	08%; 95% CI: 04–15	NR
	Patients diagnosed with NSCLC in 2007-2012		8	5-year	04%; 95% CI: 02–07	NR
	Patients diagnosed with NSCLC in 2013-2017		0	5-year	0%; 95% CI: NA	NR
	Patients diagnosed with NSCLC in 2007-2012	Stage IIIB-IV NSCLC clinically diagnosed with unknown pathology	30	1-year	08%; 95% CI: 06–11	NR
	Patients diagnosed with NSCLC in 2013-2017		14	1-year	06%; 95% CI: 04–09	NR
	Patients diagnosed with NSCLC in 2007-2012		8	3-year	02%; 95% CI: 1–5	NR
	Patients diagnosed with NSCLC in 2013-2017		<6	3-year	01%; 95% CI: 00–04	NR
	Patients diagnosed with NSCLC in 2007-2012		<6	5-year	01%; 95% CI: 00–02	NR

Study name	Patient population	Stage at diagnosis	Sample size (N)	Time point / Variable	Survival rate results	p-value
	Patients diagnosed with NSCLC in 2013-2017		0	5-year	0%; 95% CI: NA	NR
Soares 2021 (43)	Diagnosed with NSCLC between 2012-2016	Stage I	174	1-year	n (%): 143 (92); 95% CI: 88–96	NR
		Stage I	174	2-Year	n (%): 91 (80); 95% CI: 74–87	NR
		Stage II	86	1-year	n (%): 54 (71); 95% CI: 62–82	NR
		Stage II	86	2-Year	n (%): 31 (50); 95% CI: 40–63	NR
		Stage III A	235	1-year	n (%): 147 (69); 95% CI: 63–75	NR
		Stage III A	235	2-Year	n (%): 81 (46); 95% CI: 40–53	NR
	Patients diagnosed in 2015-2016-NSQ carcinoma	Stage I or II	68	1-year	89%; 95% CI: 81–97	NR
	Patients diagnosed in 2015-2016-SQ carcinoma	Stage I or II	28	1-year	76%; 95% CI: 60–95	NR
	Patients diagnosed in 2015-2016-NSQ carcinoma	Stage IIIA	41	1-year	86%; 95% CI: 75–98	NR
	Patients diagnosed in 2015-2016-SQ carcinoma	Stage IIIA	33	1-year	49%; 95% CI: 34–70	NR
Suipyte 2019 (44)	Elderly patients (>70 years) diagnosed between 2005-2007	Stage IV	NR	1-year	18.0% (95% CI: 11%–25%)	0.29 vs. elderly
	Elderly patients (>70 years) diagnosed between 2015-2016		NR	1-year	31.0% (95% CI: 22%–39%)	
	Young patients (<70 years) diagnosed between 2005-2007		NR	1-year	34.0% (95% CI: 27%–42%)	0.049 vs young
	Young patients (<70 years) diagnosed between 2015-2016		NR	1-year	52.0% (955 CI: 43%– 61%)	
Vachani 2021 (45)	Newly diagnosed patients with stage Ia	Stage Ia	NR	1-year	86.3%	NR
	Newly diagnosed patients with stage Ib	Stage Ib	NR	1-year	73.1%	NR
	Newly diagnosed patients with stage IIa	Stage IIa	NR	1-year	77.5%	NR
	Newly diagnosed patients with stage IIb	Stage IIb	NR	1-year	64%	NR
	Newly diagnosed patients with stage IIIa	Stage IIIa	NR	1-year	53.8%	NR
	Newly diagnosed patients with stage IIIb	Stage IIIb	NR	1-year	38.3%	NR

Study name	Patient population	Stage at diagnosis	Sample size (N)	Time point / Variable	Survival rate results	p-value
	Newly diagnosed patients with stage IV	Stage IV	NR	1-year	22.6%	NR
**RCC**						
**Haas 2022 (47)**	Patients with newly diagnosed localized RCC: Intermediate-high risk	Localized	629	5-year	69%	NR
	T3G1-G2 (intermediate-high risk subgroup)		297	5-year	77%	NR
	T3G3 (intermediate-high risk subgroup)		250	5-year	65%	NR
	T3G4 (intermediate-high risk subgroup)		64	5-year	37%	NR
	High risk		14	5-year	36%	NR
**Haas 2022b (48)**	Intermediate-high risk		NR	5-year	69%	NR
	High risk		NR	5-year	58%	NR
	Patients without recurrence among intermediate-high risk and high risk RCC		NR	5-year	85%	NR
	Patients with recurrence among intermediate-high risk and high risk RCC		NR	5-year	57%	NR
	Patients with recurrence among intermediate-high risk RCC		NR	5-year	58%	NR
	Patients with recurrence among high risk RCC		NR	5-year	57%	NR
**Li 2021 (49)**	ccRCC: White (after PSM)	Overall	23559	5-year	80.3%; 95% CI:78.9, 81.7	NR
		Localized	16,878	5-year	90.3%; 95% CI:89.0, 91.5	NR
		Regional	4,351	5-year	70%; 95% CI:65.8, 74.6	NR
		Distant	2330	5-year	34.1%; 95% CI:26.7, 43.5	NR
	ccRCC: Asian (after PSM)	Overall	1586	5-year	81.2%; 95% CI:78.8, 83.7	NR
		Localized	1164	5-year	91.9%; 95% CI:89.8, 94.1	NR
		Regional	261	5-year	71.5%; 95% CI:64.7, 79.0	NR
		Distant	161	5-year	20.3%; 95% CI:13.1, 31.4	NR
**TNBC**						
**Schwartz 2018 (12)**	Percentage alive at 1 year	Stage III and IV	1244	1-year	60% (95% CI: 57%–63%)	NR
	Percentage alive at 3 years	Stage III and IV	1244	3-years	33% (95% CI: 30%–36%)	NR
	All AJCC stage III cases	Stage III	828	1-year	76% (95% CI: 73%–78%)	NR

Study name	Patient population	Stage at diagnosis	Sample size (N)	Time point / Variable	Survival rate results	p-value
	All AJCC stage III cases	Stage III	828	3-years	44% (95% CI: 41%–48%)	NR
	All AJCC stage IV cases	Stage IV	416	1-year	29% (95% CI: 25%–34%)	NR
	All AJCC stage IV cases	Stage IV	416	3-years	11% (95% CI: 8%–14%)	NR
**Yousefi 2017 (54)**	Overall	Overall	180	5-year	56%	NR
	Stage I	Stage I	14	5-year	92.3%	NR
	Stage II	Stage II	88	5-year	86.5%	NR
	Stage III	Stage III	57	5-year	57.8%	NR
	Stage IV	Stage IV	8	5-year	9%	NR
**Gogate 2022 (51)**	All systemically treated patients diagnosed at early stage	Early stage	462	36 months	85.7% (SE: 1.8)	NR
**Lehrberg 2021 (52)**	Overall African American and White-American patients	Early stage	286	5-year	82% (95% CI: 77%–87%)	NR

[a] Defined as “survival relative to the total population of England.

[b] Cumulative OS

[c] Median all-cause survival

Abbreviations: 1L: First line; 2L: Second line; AJCC: American Joint Committee on Cancer; CI: Confidence interval; EGFR: Estimated glomerular filtration rate; NA: Not available; NR: Not reported; NSCLC: Non-small cell lung cancer; NSQ: Non-squamous; OS: Overall survival; RCC: Renal cell carcinoma; SD: Standard deviation; SE: Standard error; SEER: Surveillance, Epidemiology, and End Results; SQ: Squamous; TNBC: Triple negative breast cancer; TNM: Tumor (T), nodes (N), and metastases (M)

10 Risk of bias results

Quality assessment of included studies – Newcastle-Ottawa Quality Assessment Scale (cohort studies)

Study name	Selection				Comparability	Outcome			Total score	Interpretation
	Question 1: Representativeness of the exposed cohort1	Question 2: Selection of the non-exposed cohort2	Question 3: Ascertainment of exposure 3	Question 4: Demonstration that outcome of interest was not present at start of study4	Question 1: Comparability of cohorts on the basis of the design or analysis5	Question 1: Assessment of outcome6	Question 2: Was follow-up long enough for outcomes to occur7	Question 3: Adequacy of follow up of cohorts8		
**Bladder cancer**										
Aly 2019 (57)	a) *	a) *	a) *	b)	c)	b) *	a) *	d)	*****	Medium quality
Amiri 2020 (13)	a) *	c)	a) *	b)	c)	b) *	b)	a) *	****	Medium quality
Aragon-Ching 2021 (14)	a) *	c)	a) *	b)	c)	b) *	a) *	d)	****	Medium quality
Davies 2020 (15)	a) *	c)	a) *	b)	c)	b) *	a) *	d)	****	Medium quality
Fisher 2018 (1)	a) *	a) *	a) *	b)	a) **	b) *	a) *	d)	******	Medium quality
Omland 2021 (16)	a) *	a) *	a) *	b)	c)	b) *	a) *	b) *	******	Medium quality
Sorup 2021 (17)	a) *	c)	a) *	b)	c)	b) *	a)	d)	***	Low quality
Yu 2019 (56)	a) *	c)	b) *	b)	c)	d)	b)	d)	**	Low quality
**Gastric cancer**										
Dijksterhuis 2022 (18)	b) *	c)	a) *	b)	c)	b) *	b)	d)	***	Low quality
Qiu 2018 (19)	a) *	a) *	a) *	a) *	c)	b) *	b)	d)	*****	Medium quality

Study name	Selection				Comparability	Outcome			Total score	Interpretation
	Question 1: Representativeness of the exposed cohort1	Question 2: Selection of the non-exposed cohort2	Question 3: Ascertainment of exposure 3	Question 4: Demonstration that outcome of interest was not present at start of study4	Question 1: Comparability of cohorts on the basis of the design or analysis5	Question 1: Assessment of outcome6	Question 2: Was follow-up long enough for outcomes to occur7	Question 3: Adequacy of follow up of cohorts8		
**Head and neck cancer**										
Amarillo 2021 (20)	b) *	c)	d)	b)	c)	d)	a) *	d)	**	Low quality
Ho 2019 (2)	a) *	c)	a) *	a) *	c)	b) *	a) *	d)	*****	Medium quality
Hochfelder 2020 (21)	a) *	a) *	a) *	b)	c)	b) *	a) *	d)	*****	Medium quality
Neto 2021 (22)	a) *	c)	a) *	b)	c)	b) *	a) *	d)	****	Medium quality
Sargeran 2009 (24)	b) *	c)	a) *	b)	c)	b) *	a) *	b) *	*****	Medium quality
Sargeran 2008 (23)	b) *	c)	a) *	b)	c)	b) *	a) *	b) *	*****	Medium quality
**Melanoma**										
Ngo 2020 (25)	d)	c)	d)	b)	c)	d)	a) *	d)	*	Low quality
Ramond 2021 (26)	a) *	c)	a) *	b)	c)	b) *	a) *	d)	****	Medium quality
Song 2015 (4)	a) *	c)	a) *	b)	c)	b) *	a) *	d)	****	Medium quality
Tjokrowidjaja 2021 (27)	a) *	c)	a) *	b)	c)	b) *	a) *	d)	****	Medium quality
Wilson 2019 (6)	a) *	c)	a) *	b)	c)	b) *	a) *	d)	****	Medium quality
Winge-Main 2020 (28)	a) *	c)	a) *	b)	c)	b) *	a)	d)	***	Low quality
Toscano 2020 (5)	b) *	c)	b) *	b)	c)	c)	b)	d)	**	Low quality

Study name	Selection				Comparability	Outcome			Total score	Interpretation
	Question 1: Representativeness of the exposed cohort1	Question 2: Selection of the non-exposed cohort2	Question 3: Ascertainment of exposure 3	Question 4: Demonstration that outcome of interest was not present at start of study4	Question 1: Comparability of cohorts on the basis of the design or analysis5	Question 1: Assessment of outcome6	Question 2: Was follow-up long enough for outcomes to occur7	Question 3: Adequacy of follow up of cohorts8		
**NSCLC**										
Abrão 2021 (29)	a) *	c)	a) *	a) *	c)	b) *	a) *	a) *	******	Medium quality
Abrão 2022 (30)	a) *	c)	a) *	b)	c)	b) *	a) *	a) *	*****	Medium quality
Azzouqa 2019 (31)	a) *	a) *	a) *	b)	c)	b) *	a) *	d)	*****	Medium quality
Berglund 2012 (7)	a) *	c)	a) *	a) *	c)	b) *	a) *	d)	****	Medium quality
Buck 2015 (58)	a) *	c)	a) *	b)	c)	b) *	b)	d)	***	Low quality
Cerqueira 2022 (32)	a) *	c)	a) *	b)	c)	b) *	a) *	d)	****	Medium quality
Ehrenstein 2022 (8)	a) *	a) *	a) *	b)	c)	b) *	a) *	d)	*****	Medium quality
Ekman 2019 (33)	a) *	a) *	a) *	b)	c)	b) *	a) *	d)	*****	Medium quality
Flores 2021 (9)	a) *	c)	a) *	a) *	c)	b) *	a) *	d)	*****	Medium quality
Gildea 2017 (59)	a) *	c)	a) *	b)	c)	b) *	a) *	d)	****	Medium quality
Greystoke 2021 (34)	a) *	c)	a) *	b)	c)	b) *	a) *	d)	****	Medium quality
Jazieh 2021 (35)	a) *	a) *	a) *	b)	c)	b) *	b)	c)	****	Medium quality
Kalilani 2022 (36)	a) *	c)	a) *	b)	c)	b) *	a) *	d)	****	Medium quality

Study name	Selection				Comparability	Outcome			Total score	Interpretation
	Question 1: Representativeness of the exposed cohort1	Question 2: Selection of the non-exposed cohort2	Question 3: Ascertainment of exposure 3	Question 4: Demonstration that outcome of interest was not present at start of study4	Question 1: Comparability of cohorts on the basis of the design or analysis5	Question 1: Assessment of outcome6	Question 2: Was follow-up long enough for outcomes to occur7	Question 3: Adequacy of follow up of cohorts8		
Klarenbeek 2022 (37)	a) *	c)	a) *	a) *	c)	b) *	a) *	a) *	******	Medium quality
Komiya 2020 (38)	a) *	a) *	a) *	b)	c)	b) *	a) *	d)	*****	Medium quality
Luciano 2020 (39)	a) *	a) *	a) *	b)	c)	b) *	b)	d)	****	Medium quality
Martin 2022 (40)	b) *	c)	a) *	a) *	c)	b) *	a) *	d)	*****	Medium quality
Monteiro 2022 (10)	b) *	a) *	a) *	b)	c)	b) *	a) *	d)	*****	Medium quality
Potter 2022 (41)	a) *	c)	a) *	a) *	b) *	b) *	b)	d)	******	Medium quality
Ritberg 2021 (42)	d)	c)	d)	b)	c)	d)	a) *	d)	*	Low quality
Snee 2021 (11)	a) *	a) *	a) *	b)	c)	b) *	a) *	d)	*****	Medium quality
Soares 2021 (43)	a) *	a) *	a) *	b)	c)	b) *	a) *	c)	*****	Medium quality
Suipyte 2019 (44)	b) *	a) *	d)	b)	c)	d)	a) *	d)	***	Low quality
Vachani 2021 (45)	a) *	a) *	a) *	b)	c)	b) *	a) *	d)	*****	Medium quality
Van Dao 2022 (46)	a) *	a) *	a) *	b)	c)	b) *	a) *	b) *	******	Medium quality
Tjong 2021 (55)	a) *	c)	a) *	b)	c)	b) *	a) *	d)	****	Medium quality
**RCC**										
Haas 2022a (47)	a) *	c)	a) *	b)	c)	b) *	a)	d)	***	Low quality
Haas 2022b (47)	a) *	c)	a) *	b)	c)	b) *	a) *	d)	****	Medium quality

Study name	Selection				Comparability	Outcome			Total score	Interpretation
	Question 1: Representativeness of the exposed cohort1	Question 2: Selection of the non-exposed cohort2	Question 3: Ascertainment of exposure 3	Question 4: Demonstration that outcome of interest was not present at start of study4	Question 1: Comparability of cohorts on the basis of the design or analysis5	Question 1: Assessment of outcome6	Question 2: Was follow-up long enough for outcomes to occur7	Question 3: Adequacy of follow up of cohorts8		
Li 2021 (49)	a) *	c)	a) *	b)	c)	b) *	a) *	a) *	*****	Medium quality
**TNBC**										
Aly 2019a (50)	a) *	a) *	a) *	b)	c)	b) *	a) *	d)	*****	Medium quality
Gogate 2022 (51)	a) *	c)	a) *	b)	c)	b) *	a) *	b) *	*****	Medium quality
Haiderali 2021 **(60)**	a) *	a) *	a) *	b)	c)	b) *	a) *	d)	*****	Medium quality
Lehrberg 2021 (52)	b) *	c)	a) *	a) *	c)	b) *	a) *	a) *	******	Medium quality
Schwartz 2018 (12)	a) *	c)	a) *	b)	c)	b) *	a) *	d)	****	Medium quality
Sieluk 2020 (53)	a) *	a) *	a) *	b)	a) **	b) *	b)	d)	*****	Medium quality
Yousefi 2017 (54)	a) *	c)	a) *	b)	c)	b) *	a) *	a) *	*****	Medium quality

[1] a) truly representative of the average ____ (describe) in the community (*) ;b) somewhat representative of the average ___ in the community (*); c) selected group of users e.g., nurses, volunteers; d) no description of the derivation of the cohort
[2] a) drawn from the same community as the exposed cohort (*); b) drawn from a different source; c) no description of the derivation of the non-exposed cohort
[3] a) secure record (*); b) structured interview (*); c) written self-report; d) no description
[4] a) yes (*); b) no
[5] a) study controls for ___ (select the most important factor) (**); b) study controls for any additional factor (*); c) Cohort are not comparable on the basis of design or analysis controlled for confounders
[6] a) independent blind assessment (*); b) record linkage (*); c) self-report; d) no description
[7] a) yes (*); b) no
[8] a) complete follow up - all subjects accounted for (*); b) subjects lost to follow up unlikely to introduce bias - small number lost - > ____ % follow up, or description provided of those lost) (*) c) follow up rate <____% and no description of those lost; d) no statement

Abbreviations: NSCLC: Non-small cell lung cancer; RCC: Renal cell carcinoma; TNBC: Triple negative breast cancer

Quality assessment of included studies – Cost of Illness study checklist

Study name	Cost-of-Illness Study checklist		Study name: Singh 2021
**(1) Analytical framework: what costs should have been measured?**	(a) What was the motivation and perspective of the study?		This study was performed from a health care provider's perspective
	b) Was the appropriate epidemiologic approach taken?		Yes
	(c) Was the study question well specified?	(i) Were all relevant, non-trivial cost components and their stakeholders identified?	Yes
		(ii) Were necessary timeframes specified?	Yes
		(iii) Was a case of disease or risk factor adequately and appropriately defined?	Yes
		(iv) Was the counterfactual population occurrence plausible and meaningful?	Unclear
**(2) Methodology and data: how well were resource use and productivity losses measured?**	(a) Was an appropriate method(s) of quantification used, such that	(i) additional, or excess, costs were measured?	Yes
		(ii) only costs specific to (caused by) the health problem were included (confounders controlled)?	Yes
		(iii) all important effects were captured?	Yes
		(iv) important differences across subpopulations were accounted for?	Yes
		(v) the required level of detail could be provided?	Yes
	(b) Was the resource quantification method(s) well executed?	(i) For population-based studies, were cost allocation methods, data and assumptions valid?	NR
		(ii) For person-based studies, were appropriate statistical tests performed and reported?	Yes
		(iii) Were data representative of the study population?	Yes
		(iv) Were there any other relevant resource quantification issues?	Unclear
	c) Were healthcare resources valued appropriately?		No
	(d) Was the approach for valuing production losses justified, and assumptions valid?		Unclear
	(e) Was the inclusion of intangible costs appropriate:	(i) Was double counting of mortality-related production losses avoided?	NR
		(ii) Were losses valued appropriately, given the study's perspective?	NR
**(3) Analysis and reporting**	(a) Did the analysis address the study question?		Yes
	(b) Was a range of estimates presented?		Yes
	(c) Were the main uncertainties identified?		Yes
	d) Was a sensitivity analysis performed on:	(i) important (uncertain) parameter estimates?	Yes
		(ii) key assumptions? (including the counterfactual)	Unclear
		(iii) point estimates? (based on confidence or credible intervals)	Unclear
	e) Was adequate documentation and justification given for cost components, data and sources, assumptions and methods?		Yes
	f) Was uncertainty around the estimates and its implications adequately discussed?		Yes
	(g) Were important limitations discussed regarding the cost components, data, assumptions and methods?		Yes
	(h) Were the results presented at the appropriate level of detail to answer the study question (cost components; disease subtypes, severity, stage; subpopulation groups, cost bearers)?		Yes

11 References

1. Fisher MD, Shenolikar R, Miller PJ, Fenton M, Walker MS. Treatment Patterns and Outcomes in Stage IV Bladder Cancer in a Community Oncology Setting: 2008-2015. Clin Genitourin Cancer. 2018;16(6):e1171-e9; doi:10.1016/j.clgc.2018.07.025.

2. Ho PS, Wang WC, Huang YT, Yang YH. Finding an oral potentially malignant disorder in screening program is related to early diagnosis of oral cavity cancer - Experience from real world evidence. Oral Oncol. 2019;89:107-14; doi:10.1016/j.oraloncology.2018.12.007.

3. Singh AG, Chaukar D, Gupta S, Pramesh CS, Sullivan R, Chaturvedi P, et al. A prospective study to determine the cost of illness for oral cancer in India. Ecancermedicalscience. 2021;15:1252; doi:10.3332/ecancer.2021.1252.

4. Song X, Zhao Z, Barber B, Farr AM, Ivanov B, Novich M. Overall survival in patients with metastatic melanoma. Curr Med Res Opin. 2015;31(5):987-91; doi:10.1185/03007995.2015.1021904.

5. Toscano A, Blanchin M, Bourdon M, Bonnaud Antignac A, Sebille V. Longitudinal associations between coping strategies, locus of control and health-related quality of life in patients with breast cancer or melanoma. Qual Life Res. 2020;29(5):1271-9; doi:10.1007/s11136-019-02401-8.

6. Wilson MA, Zhong J, Rosenbaum BE, Utter K, Moran U, Darvishian F, et al. Impact of initial stage on metastatic melanoma survival. Melanoma Res. 2019;29(3):281-8; doi:10.1097/CMR.0000000000000526.

7. Berglund A, Lambe M, Luchtenborg M, Linklater K, Peake MD, Holmberg L, et al. Social differences in lung cancer management and survival in South East England: a cohort study. BMJ Open. 2012;2(3); doi:10.1136/bmjopen-2012-001048.

8. Ehrenstein V, Eriksen K, Taylor A, Servidio L, Jakobsen E. Characteristics and overall survival of patients with early-stage non-small cell lung cancer: A cohort study in Denmark. Cancer Med. 2022; doi:10.1002/cam4.4946.

9. Flores R, Patel P, Alpert N, Pyenson B, Taioli E. Association of Stage Shift and Population Mortality Among Patients With Non-Small Cell Lung Cancer. JAMA Netw Open. 2021;4(12):e2137508; doi:10.1001/jamanetworkopen.2021.37508.

10. Monteiro AS, Araujo SRC, Araujo LH, Souza MC. Impact of microvascular invasion on 5-year overall survival of resected non-small cell lung cancer. J Bras Pneumol. 2022;48(3):e20210283; doi:10.36416/1806-3756/e20210283.

11. Snee M, Cheeseman S, Thompson M, Riaz M, Sopwith W, Lacoin L, et al. Treatment patterns and survival outcomes for patients with non-small cell lung cancer in the UK in the preimmunology era: a REAL-Oncology database analysis from the I-O Optimise initiative. BMJ Open. 2021;11(9):e046396; doi:10.1136/bmjopen-2020-046396.

12. Schwartz KL, Simon MS, Bylsma LC, Ruterbusch JJ, Beebe-Dimmer JL, Schultz NM, et al. Clinical and economic burden associated with stage III to IV triple-negative breast cancer: A SEER-Medicare historical cohort study in elderly women in the United States. Cancer. 2018;124(10):2104-14; doi:10.1002/cncr.31299.

13. Amiri M, Heshmatollah S, Esmaeilnasab N, Khoubi J, Ghaderi E, Roshani D. Survival rate of patients with bladder cancer and its related factors in Kurdistan Province (2013-2018): a population-based study. BMC Urol. 2020;20(1):195; doi:10.1186/s12894-020-00769-1.

14. Aragon-Ching JB, Wang H. Comparative analyses of survival differences in patients with urothelial versus non-urothelial upper tract carcinomas: Results from the National Cancer Database (NCDB). Journal of Clinical Oncology. 2021;39(15_suppl):e16582-e; doi:10.1200/JCO.2021.39.15_suppl.e16582.

15. Davies FJ, Knott C, Kerr C, Adedokun L, Lockhat DM. Utilising Public Health England Datasets to Establish a Standing Cohort of Patients with Metastatic Bladder Cancer: Initial Results and Algorithm Defining Disease Progression. Value Health. 2020;23(Suppl. 2):S480-S1.

16. Omland LH, Lindberg H, Carus A, Als AB, Jensen NV, Taarnhøj GA, et al. Real-world Treatment Patterns and Overall Survival in Locally Advanced and Metastatic Urothelial Tract Cancer Patients Treated with Chemotherapy in Denmark in the Preimmunotherapy Era: A Nationwide, Population-based Study. Eur Urol Open Sci. 2021;24:1-8; doi:10.1016/j.euros.2020.12.002.

17. Sørup S, Darvalics B, Kearney M, Mahmoudpour SH, Oksen D, Kolitsopoulos F, et al. Treatment Patterns, Clinical Outcomes, And Healthcare Resource Use Among Patients With Stage IV Urothelial Cancer: Findings From A Nationwide Cohort Study In Denmark. Value in Health. 2021;24(S65); doi:10.1016/j.jval.2021.04.332.

18. Dijksterhuis WPM, Kroese TE, Verhoeven RHA, van Rossum PSN, Mook S, Haj Mohammad N, et al. A population-based study on treatment and outcomes in patients with gastric adenocarcinoma diagnosed with distant interval metastases. Eur J Surg Oncol. 2022;48(9):1964-71; doi:10.1016/j.ejso.2022.03.003.

19. Qiu MZ, Shi SM, Chen ZH, Yu HE, Sheng H, Jin Y, et al. Frequency and clinicopathological features of metastasis to liver, lung, bone, and brain from gastric cancer: A SEER-based study. Cancer Med. 2018;7(8):3662-72; doi:10.1002/cam4.1661.

20. Amarillo D, Herrera G, Lara G, Muñóz E, Sanchez S, Sommer P, et al. P-216 Impact of delay in treatment initiation in Overall Survival in Head and Neck Cancer. Oral Oncology. 2021;118:3; doi:10.1016/S1368-8375(21)00499-1.

21. Hochfelder GC, McGinn AP, Mehta V, Castellucci E, Kabarriti R, Julian Ow T. First-Line Surgery Is Associated with Longer Overall Survival in Locoregionally Advanced Hypopharyngeal Cancer in the National Cancer Database. Journal of the American College of Surgeons. 2020;231(4):S190; doi:10.1016/j.jamcollsurg.2020.07.313.

22. Neto FL, Sousa LG, Johnson FM, Lee JJ, Frank SJ, Moreno AC, et al. Comparative assessment of the eighth and seventh AJCC staging edition prognostic performance of patients with p16 positive oropharynx cancer. Journal of Clinical Oncology. 2021;39(15_suppl):6067-; doi:10.1200/JCO.2021.39.15_suppl.6067.

23. Sargeran K, Murtomaa H, Safavi SMR, Vehkalahti MM, Teronen O. Survival after diagnosis of cancer of the oral cavity. Br J Oral Maxillofac Surg. 2008;46(3):187-91; doi:10.1016/j.bjoms.2007.11.004.

24. Sargeran K, Murtomaa H, Safavi SMR, Vehkalahti MM. Survival After Lip Cancer Diagnosis. The Journal of Craniofacial Surgery. 2009;20(1); doi:10.1097/SCS.0b013e31818431bd.

25. Ngo P, Ko J, Sussman TA, Li H, Hobbs B, Gastman B, et al. Survival and recurrence patterns of stage II and III cutaneous melanoma by AJCC 8th edition staging. Pigment Cell and Melanoma Research. 2020;33(1):213.

26. Ramond A, Carroll R, Vekeria S, Nordstrom B. Investigating Secular Trends in the Survival of Melanoma Patients in England. The International Society for Pharmacoeconomics and Outcomes Research (ISPOR). 2021;24(1); doi:10.1016/j.jval.2021.04.206.

27. Tjokrowidjaja A, Browne L, Soudy H. External validation of the American Joint Committee on Cancer melanoma staging system eighth edition using the surveillance, epidemiology, and end results program. Asia Pac J Clin Oncol. 2022;18(5):e280-e8; doi:10.1111/ajco.13689.

28. Winge-Main A, Robsahm TE, Nyakas MS, Festervoll G, Torkilseng E, Thybo S, et al. Long-term outcomes of stage IIB-IV melanoma patients: Nationwide data from Norway. Annals of Oncology. 2020;31(Suppl. 4):S766-S7; doi:10.1016/j.annonc.2020.08.1273.

29. Abrao FC, Peres SV, de Abreu I, Younes RN. Prognostic factors and patients' profile in treated stage I and II lung adenocarcinoma: a Hospital's Cancer Registry-based analysis. J Thorac Dis. 2021;13(11):6294-303; doi:10.21037/jtd-21-1071.

30. Abrao FC, de Abreu I, Silva VG, Rosamilia GA, Peres SV, Hanriot RM, et al. Overall survival and prognostic factors in Stage I lung adenocarcinoma treated with curative intent: A real-life 19-year cohort study. J Surg Oncol. 2022;126(6):1114-22; doi:10.1002/jso.27015.

31. Azzouqa A-G, Chen R, Lou Y, Ailawadhi S, Manochakian R. Impact of time to treatment initiation (TTI) on survival of patients with newly diagnosed non-small cell lung cancer (NSCLC). Journal of Clinical Oncology. 2019;37(15_suppl):9058-; doi:10.1200/JCO.2019.37.15_suppl.9058.

32. Cerqueira ER, Padula AC, Alencar Junior FO, Rego MADC, Almeida M, de Mendonça Batista P, et al. Characterization of Clinical Features and Treatment of Non-Small Cell Lung Cancer (NSCLC) STAGE III and IV in Brazil- a Retrospective DATA-Base Study. Value in Health. 2022;25(7):S443-S4; doi:10.1016/j.jval.2022.04.803.

33. Ekman S, Sørensen JB, Brustugun OT, Horvat P, Patel D, Rosenlund M, et al. Treatment (Tx) patterns and overall survival (OS) in patients (pts) with NSCLC in Sweden : A SCAN-LEAF study analysis from the I-O Optimise initiative. Annals of Oncology. 2019;30(Suppl 2):II17; doi:10.1093/annonc/mdz070.005.

34. Greystoke A, Nawaz A, Ferguson S, Pabla M, Sanjeevi P, Bar-Ziv O. P09.23 Real-World Treatment Patterns and Outcomes in Patients with NSCLC in England (ROSANNE): A Retrospective Study. Journal of Thoracic Oncology. 2021;16:S299-S300; doi:10.1016/j.jtho.2021.01.451.

35. Jazieh AR, Onal HC, Tan DSW, Soo RA, Prabhash K, Kumar A, et al. Real-World Treatment Patterns and Clinical Outcomes in Patients With Stage III NSCLC: Results of KINDLE, a Multicountry Observational Study. J Thorac Oncol. 2021;16(10):1733-44; doi:10.1016/j.jtho.2021.05.003.

36. Kalilani L, Chao J, Hogea C, Stojadinovic A, Giove TJ, Sun X, et al. Recent Estimates of Survival in Patients with Advanced Non-Small Cell Lung Cancer (NSCLC) in the US (2010-2020). The International Society for Pharmacoeconomics and Outcomes Research (ISPOR). 2022;25(Suppl 4); doi:10.1016/j.jval.2021.11.015.

37. Klarenbeek SE, Aarts MJ, van den Heuvel MM, Prokop M, Tummers M, Schuurbiers OCJ. Impact of time-to-treatment on survival for advanced non-small cell lung cancer patients in the Netherlands: a nationwide observational cohort study. Thorax. 2022;78(5):467-75; doi:10.1136/thoraxjnl-2021-218059.

38. Komiya T, Powell E, Vu C, Guddati AK. Role of T0 status in overall survival for unresectable stage III non-small cell lung cancer. Journal of Clinical Oncology. 2020;38(15_suppl):9026-; doi:10.1200/JCO.2020.38.15_suppl.9026.

39. Luciano S, Haudenschild C, Kuranz S. Using real world data to examine outcomes in immunotherapy-treated patients with metastatic non-small cell lung cancer. Journal of Clinical Oncology. 2020;38(15_suppl):e21715-e; doi:10.1200/JCO.2020.38.15_suppl.e21715.

40. Martin CM, Puello-Guerrero A, Mas-Lopez LA, Campos-Gomez S, Orlando-Orlandi FJ, Tejado Gallegos LF, et al. Real-world KINDLE-Latin America subset data on treatment patterns and clinical outcomes in patients with stage III non-small-cell lung cancer. Cancer Med. 2022;12(2):1247-59; doi:10.1002/cam4.4990.

41. Potter AL, Rosenstein AL, Kiang MV, Shah SA, Gaissert HA, Chang DC, et al. Association of computed tomography screening with lung cancer stage shift and survival in the United States: quasi-experimental study. BMJ. 2022;376:e069008; doi:10.1136/bmj-2021-069008.

42. Rittberg R, Bucher O, Xue L, Aurangzeb Z, Banerji SO, Dawe D. Population-based impacts of new therapies on outcomes for stage IV non-small cell lung cancer. Journal of Clinical Oncology. 2021;39(15_suppl):9120-; doi:10.1200/JCO.2021.39.15_suppl.9120.

43. Soares M, Antunes L, Redondo P, Borges M, Hermans R, Patel D, et al. Treatment and outcomes for early non-small-cell lung cancer: a retrospective analysis of a Portuguese hospital database. Lung Cancer Manag. 2021;10(2):LMT46; doi:10.2217/lmt-2020-0028.

44. Suipyte J, Schmidt S, Herrmann C, Mousavi M, Hitz F, Fruh M. Does progress achieved in the treatment of patients with metastatic non-small cell lung cancer (NSCLC) reach the elderly population? Annals of Oncology. 2019;30(Suppl 5):v650; doi:10.1093/annonc/mdz260.100.

45. Vachani A, Johnson B, Johnston S, Johnson W, Chandran U, Sengupta N, et al. ASSOCIATION OF STAGE AT DIAGNOSIS OF NON-SMALL CELL LUNG CANCER WITH OVERALL SURVIVAL AND ONE-YEAR HEALTHCARE EXPENDITURES: AN ANALYSIS OF THE SEER-MEDICARE DATABASE. Chest. 2021;160(4):A1608-A9; doi:10.1016/j.chest.2021.07.1468.

46. Van Dao T, Diep TB, Le Phuong T, Huggenberger R, Kumar A. Real-World Treatment Patterns and Clinical Outcomes in Patients With Stage III Non-Small-Cell Lung Cancer: Results of KINDLE-Vietnam Cohort. Front Oncol. 2022;12:842296; doi:10.3389/fonc.2022.842296.

47. Haas NB, Bhattacharya R, Song Y, Rogerio J, Zhang S, Carley C, et al. Variation in recurrence rate and overall survival (OS) outcomes by disease stage and incremental impact of time to recurrence on OS in localized renal cell carcinoma (RCC). Journal of Clinical Oncology. 2022;40(16_suppl):4543-; doi:10.1200/JCO.2022.40.16_suppl.4543.

48. Haas NB, Bhattacharya R, Ogbomo AS, Imai K, Gautam S, Rogerio J, et al. Disease-free and overall survival outcomes for localized RCC patients by disease stage. Journal of Clinical Oncology. 2022;40(16_suppl):e16526-e; doi:10.1200/JCO.2022.40.16_suppl.e16526.

49. Li X, Xu Z, Xu T, Qi F, Song N. Basic Characteristics and Survival Outcomes of Asian-American Patients with Clear Cell Renal Cell Carcinoma and Comparisons with White Patients: A Population-Based Analysis. Int J Gen Med. 2021;14:7869-83; doi:10.2147/IJGM.S340284.

50. Aly A, Shah R, Hill K, Botteman MF. Overall survival, costs and healthcare resource use by number of regimens received in elderly patients with newly diagnosed metastatic triple-negative breast cancer. Future Oncol. 2019;15(9):1007-20; doi:10.2217/fon-2018-0407.

51. Gogate A, Crosbie A, Le TK, Zhang Y, Das R, Davis C. Clinical characteristics, treatment patterns, and survival outcomes in women with early triple-negative (TN) or hormone receptor-positive/human epidermal growth factor receptor-2 negative (HR(+)/HER2-) breast cancer (BC) in the real-world (RW) setting. Cancer Res. 2022;82(4_Supplement):P3-12-5; doi:10.1158/1538-7445.SABCS21-P3-12-15.

52. Lehrberg A, Davis MB, Baidoun F, Petersen L, Susick L, Jenkins B, et al. Outcome of African-American compared to White-American patients with early-stage breast cancer, stratified by phenotype. Breast J. 2021;27(7):573-80; doi:10.1111/tbj.14225.

53. Sieluk J, Haiderali A, Huang M, Yang L, Tryfonidis K, Hirshfield KM. SEER-Medicare study of early-stage triple-negative breast cancer: Real-world treatment patterns, survival, and expenditures 2010 to 2016. Journal of Clinical Oncology. 2020;38(15); doi:10.1200/JCO.2020.38.15_suppl.e1251.

54. Yousefi Kashi AS, Yazdanfar S, Akbari M-E, Rakhsha A. Triple Negative Breast Cancer in Iranian Women: Clinical Profile and Survival Study. International Journal of Cancer Management. 2017;10(8):e10471; doi:10.5812/ijcm.10471.

55. Tjong MC, Doherty M, Tan H, Chan WC, Zhao H, Hallet J, et al. Analysis of Patient Reported Outcomes for Stage IV Non-Small Cell Lung Cancer Patients in Ontario. International Journal of Radiation Oncology*Biology*Physics. 2021;111(3, Supplement):e164-e5; doi:10.1016/j.ijrobp.2021.07.640.

56. Yu EY, Nekeman D, Billingham LJ, James ND, Cheng KK, Bryan RT, et al. Health-related quality of life around the time of diagnosis in patients with bladder cancer. BJU Int. 2019;124(6):984-91; doi:10.1111/bju.14804.

57. Aly A, Johnson C, Doleh Y, Shenolikar R, Hussain A, editors. Lifetime costs of urothelial carcinoma by stage at diagnosis. Journal of Managed Care and Specialty Pharmacy; 2019.

58. Buck PO, Saverno KR, Miller PJ, Arondekar B, Walker MS. Treatment Patterns and Health Resource Utilization Among Patients Diagnosed With Early Stage Resected Non-Small Cell Lung Cancer at US Community Oncology Practices. Clin Lung Cancer. 2015;16(6):486-95; doi:10.1016/j.cllc.2014.12.010.

59. Gildea TR, DaCosta Byfield S, Hogarth DK, Wilson DS, Quinn CC. A retrospective analysis of delays in the diagnosis of lung cancer and associated costs. Clinicoecon Outcomes Res. 2017;9:261-9; doi:10.2147/CEOR.S132259.

60. Haiderali A, Rhodes WC, Gautam S, Huang M, Sieluk J, Skinner KE, et al. Real-world treatment patterns and effectiveness outcomes in patients with early-stage triple-negative breast cancer. Future Oncol. 2021;17(29):3819-31; doi:10.2217/fon-2021-0530.

61. Jazieh AR, Onal HC, Tan DS-W, Soo RA, Prabhash K, Kumar A, et al. Contemporary management and associated outcomes of 3,151 patients with stage III non-small cell lung cancer (NSCLC) in a real-world setting: Results of KINDLE, a multicountry observational study. Journal of Clinical Oncology. 2020;38(15_suppl):9043-; doi:10.1200/JCO.2020.38.15_suppl.9043.
